# Design, Synthesis, and Testing of 1,2,3-Triazolo-Quinobenzothiazine Hybrids for Cytotoxic and Immunomodulatory Activity

**DOI:** 10.3390/ijms26146920

**Published:** 2025-07-18

**Authors:** Klaudia Giercuszkiewicz-Haśnik, Magdalena Skonieczna, Beata Morak-Młodawska, Małgorzata Jeleń

**Affiliations:** 1 Department of Systems Biology and Engineering, The Silesian University of Technology, Akademicka Street 16, 44-100 Gliwice, Poland; klaudia.giercuszkiewicz@polsl.pl (K.G.-H.); magdalena.skonieczna@polsl.pl (M.S.); 2 Centre of Biotechnology, Silesian University of Technology, Krzywoustego Street 8, 44-100 Gliwice, Poland; 3 Department of Organic Chemistry, Faculty of Pharmaceutical Sciences in Sosnowiec, Medical University of Silesia in Katowice, Jagiellońska Street 4, 41-200 Sosnowiec, Poland; bmlodawska@sum.edu.pl

**Keywords:** quinobenzothiazine, inflammatory response, apoptosis, RT-qPCR analysis, BEAS-2B, BCL2, MDM2, AIFM2, IL6, IL8

## Abstract

Phenothiazines, mainly known for their antipsychotic activity, have recently attracted attention as potential compounds with anticancer and immunomodulatory activity In this study, 20 new quinobenzothiazines (**MJ1**–**MJ20**) were synthesized and their effects on normal cell lines (BEAS-2B, NHDF) and cancer cell lines (HCT116, MCF7, A549, SH-SY5Y, U2OS) were investigated. The studies included cytotoxicity assessment, analysis of the expression of genes (BCL2, AIFM2, MDM2) and pro-inflammatory cytokines (IL6, IL8) using the RT-qPCR method, and prediction of biological activity using the PASS platform. The results indicate that the compounds **MJ19** and **MJ20** have the greatest effect on the induction of pro-inflammatory (IL6, IL8) and antiapoptotic (BCL2, MDM2) genes, suggesting their potential use in therapies for inflammatory and autoimmune diseases. Gene expression analysis showed that compound **MJ2** in BEAS-2B cells significantly induced the expression of AIFM2, a protein responsible for protecting against ferroptosis, while moderately increasing the expression of BCL2 and MDM2, suggesting a potential role for **MJ2** in the modulation of protective mechanisms of healthy cells, e.g., avoiding apoptosis death. These results emphasize the potential of quinobenzothiazines as multifunctional bioactive compounds, which require further studies to determine their mechanisms of action and specificity.

## 1. Introduction

The phenothiazine derivatives are the oldest synthetic group of tricyclic neuroleptic drugs and have been known for over 100 years and are one of the most versatile compounds from the point of view of biological activity. In recent years, hundreds of articles have been published on the synthesis and biological activity of these compounds. This research has made it possible to create many derivatives, some of which are used in therapy. Currently, these compounds are used in psychiatry, particularly in the treatment of schizophrenia, manic states, and various psychoses [1,2,3].

Classical phenothiazines, substituted in position 10 with dialkylaminoalkyl groups and additionally in position 2 with small groups (-H, -Cl, -CF_3_, SCH_3_), exhibit significant activities such as neuroleptic, antiemetic, antihistaminic, antipuritic, analgesic, and antihelmintic. For some time there have also been reports of promising anticancer, antibacterial, antiplasmid, multidrug resistance (MDR) reversal activities and potential treatment in Alzheimer’s and Creutzfeldt-Jakob diseases of classical phenothiazines [4,5,6,7,8,9,10,11,12,13].

Modifications of phenothiazine is mostly carried out mainly by changing the substituent in the thiazine nitrogen atom, introduction of substituents into the benzene ring, oxidation of the sulfur atom of the thiazine ring to the sulfoxide or sulfone system and replacing one or two benzene rings by the heteroaromatic system (pyridine, pyridazine, pyrimidine, pyrazine, 1,2,4-triazine, quinoline, quinoxaline) or bicyclic homoaromatic system (naphthalene) [14,15,16,17,18,19].

The techniques of modifying the neuroleptic phenothiazine skeleton lead to modified phenothiazine systems that show a significant spectrum of biological activities, including: anticancer, antiplasmid, antiviral, anti-inflammatory, and antibacterial activities, reversal of multidrug resistance, potential treatment in Alzheimer’s and Creutzfeldt-Jakob diseases, butyrylcholinesterase inhibition, while they also exhibit antioxidant and antihyperlipidemic activity [1,20,21,22].

Triazoles are heterocyclic organic compounds containing five member rings with three nitrogen atoms and two carbon atoms. There were two isomeric forms of triazoles, namely 1,2,3-triazole and 1,2,4-triazole [23,24,25].

The 1,2,3-triazoles, due to their unique chemical and structural properties, have received a great deal of attention over the past several decades and have been well recognized for their wide range of pharmacological properties. The 1,2,3-triazole derivatives have been well exploited for the generation of many medicinal scaffolds that exhibit anti-HIV, anticancer, and antibacterial activities. The 1,2,3-triazole ring when coupled with another heterocyclic ring has manifested wide therapeutic potential such as: antitumor, anti-inflammatory, analgesic, anti-HIV, antifungal, antimicrobial, anticonvulsant, antioxidant, antitubercular, antiparasitic, antithrombotic, and antidepressant. 1,2,3-Triazole derivatives have also been used as enzyme inhibitors such as histone deacetylase, PDE4 (phosphodiesterase 4), alkaline phosphatase, cysteine protease and acetylcholinesterase. Several triazoles (for example, cefatrizine, tazobactam) were used in medicine as β-lactam antibiotics. Furthermore, 1,2,3-triazoles can form hydrogen bonds, which play an important role in bioavailability and solubility. The triazole ring is a biological linker and exhibits bioisosteric effects on various heteroaromatic and aromatic rings [26,27,28,29,30,31,32,33].

**Scheme 1:**
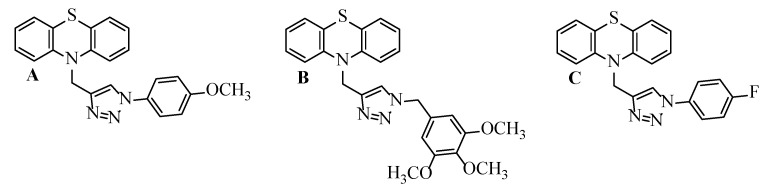
Anticancer 1,2,3-triazole-phenothiazine hybrids.

The structural modifications of the classical phenothiazine system are carried out by replacing the substituent at the nitrogen, among others, by incorporating the 1,2,3-triazole ring. Liu et al. [34] investigated the anticancer activity of the new phenothiazine-1,2,3-triazole hybrids. Their antiproliferative activity against three gastric cancer cell lines (MKN28, MGC-803, and MKN45) was evaluated. One of the described compounds showed high inhibitory activity against MGC-803 gastric cancer cells with an IC_50_ value of 1.2 μM (compound **A**, Scheme 1). It was found that this derivative could inhibit migration by regulating the expression level of N-cadherin, E-cadherin, vimentin and active d-MMP2. Furthermore, it could regulate the wnt/β-catenin signaling pathway in MGC-803 cells in a concentration-dependent manner by reducing the expression level of Wnt5α, β-catenin and TCF4. This hybrid was also a novel inhibitor of tubulin polymerization. In an oral study, it was demonstrated that this compound could effectively inhibit the growth of xenografted MGC-803 tumor in vivo without apparent side effects [34]. Zhang et al. [35] investigated how introducing a 1,2,3-triazole substituent into the phenothiazine system affects anticancer activity. The researchers designed a series of novel phenothiazine-1,2,3-triazole hybrids and evaluated their antiproliferative activity against three cancer cell lines: MDA-MB-231, MDA-MB-468, and MCF-7. When the 1,2,3-triazole group was removed, inhibitory activity against all cancer cell lines was decreased (IC_50_ > 100 μM). The most active compound showed IC_50_ values ranging from 0.8 μM to 1.7 μM (compound **B**, Scheme 1). It was found to induce apoptosis of MCF-7 cells through regulation of apoptosis-related proteins (Bcl-2, Bax, Bad, Parp, and DR5) [35]. 1,2,3-Triazole-phenothiazines have also been reported to exhibit in vitro activity against human gastric cancer (MGC-803), human esophageal cancer (EC-109), human prostate cancer (PC-3), human breast cancer (MCF-7), and hepatocellular carcinoma (HepG-2). The IC_50_ values for these hybrids ranged from 0.5 to 27.3 μM. The introduction of a fluorine atom into the benzene ring of the substituent was judged to lead to an increase in activity (compound **C**, Scheme 1) [36].

**Scheme 2:**
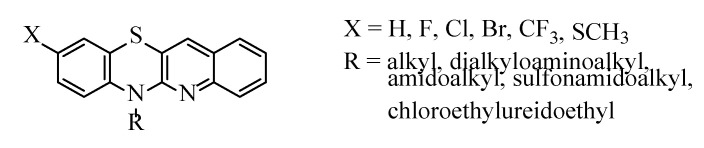
Quinobenzothiazines with immunosuppressive and antitumor activity.

The aim of our studies was to obtain new phenothiazine hybrids by replacing one of the benzene rings with a quinoline system and introducing a 1,2,3-triazole ring-containing substituent to the thiazine nitrogen atom. Some of the previously obtained quinobenzothiazines with different substituents on the thiazine nitrogen atom showed promising immunosuppressive and antitumor activity against human cancer cell lines derived from the colon, breast, kidney, lung, ovary, prostate, central nervous system, melanoma and leukemia. Scheme 2 shows the overall structures of the quinobenzothiazines that have been synthesized and tested for various biological activities [37,38,39].

Recent studies indicate that phenothiazines may have potential anticancer effects by influencing various molecular mechanisms in cells [40,41,42]. Their actions include inhibiting cell proliferation, modulating signaling pathways such as PDK1/Akt and MAPK/ERK1/2, inducing apoptosis by inhibiting the Akt/mTOR pathway, and blocking angiogenesis by inhibiting VEGF (vascular endothelial growth factor) production and VEGF-dependent signaling. This study focused on evaluating the effect of guinobenzothiazines on cell survival, morphology, and expression of genes related to apoptosis, ferroptosis, and the inflammatory response, which may contribute to a better understanding of their potential therapeutic properties.

The selected research genes—BCL2 (B-cell CLL/lymphoma 2), MDM2 (mouse double minute 2 homolog) and AIFM2 (Apoptosis-Inducing Factor, Mitochondrion-Associated, 2)—play key roles in cell survival processes. The BCL2 gene encodes an antiapoptotic protein that protects mitochondria from damage by inhibiting the apoptosis process. BCL2 overexpression is often observed in cancer cells and is associated with resistance to cytotoxic therapy [38]. MDM2 is a negative regulator of the p53 protein, which controls the cell cycle and apoptosis in response to cellular stress [43]. AIFM2, also known as Ferroptosis Suppressor Protein 1 (FSP1), plays a key role in protecting cells from ferroptosis, a regulated form of cell death induced by oxidative stress and lipid peroxidation [44]. High expression of AIFM2 may support cell survival under environmental stress.

The study used the normal BEAS-2B cell line, representing human bronchial epithelial cells, NHDF (Normal Human Dermal Fibroblast cells) and various cancer cell lines, including HCT116 (colon cancer), A549 (lung cancer), MCF7 (breast cancer), SH-SY5Y (neuroblastoma) and U2OS (osteosarcoma). These lines were selected due to their biological diversity, which allows for the assessment of the activity of the tested compounds in different molecular and tissue contexts.

The aim of this study was to evaluate the effect of quinobenzothiazines (**MJ1**–**MJ20**) on cell survival, changes in their morphology, and the expression of genes related to the mechanisms of cell survival and death. These studies are not only to identify potential mechanisms of action of quinobenzothiazines but also to assess their usefulness in the context of anticancer therapies and modulation of the inflammatory response.

## 2. Results and Discussion

### 2.1. Synthesis of 1,2,3-Triazolo-Quinobenzothiazine Hybrids

One of the modern drug design strategies is hybrid drug design (molecular hybridization), which involves the rational combination of structural fragments derived from two (or more) known drugs or ligands that exhibit biological activity against a specific molecular target. The goal of this method is to obtain a new chemical compound that combines the favorable pharmacodynamic properties of the two starting structures, and at the same time may have a better pharmacokinetic profile, reduced toxicity or the ability to overcome drug resistance [45,46,47]. The hybridization strategy has found applications in the design of enzyme inhibitors, anticancer, anti-infective or neuroprotective drugs, among others. An example is the synthesis of hybrid acetylcholinesterase and β-amyloid inhibitors for the treatment of Alzheimer’s disease, in which fragments of donepezil and other inhibitors were combined in a single molecule to achieve a multi-targeted effect [48]. Hybrid drug design is considered a promising approach in the context of polypharmacology, enabling the creation of compounds that act on several biological targets simultaneously, which is particularly important in the treatment of complex diseases such as cancer, neurodegenerative diseases or infectious diseases [49]. As we demonstrated in the introduction, both phenothiazines and 1,2,3-triazoles are valuable chemical motifs in drug design due to their broad spectrum of biological activities. Therefore, the purpose of our study was to synthesize and evaluate for immunomodulatory and cytotoxic activity against selected cancer cell lines hybrids of quinobenzothiazines with 1,2,3-triazoles. Hybridization of phenothiazines with 1,2,3-triazoles involves the incorporation of a triazole ring to a thiazine nitrogen atom. The synthesis of such hybrids often relies on the Huisgen cycloaddition reaction, known as “click chemistry,” which allows efficient and selective formation of a triazole bond between the corresponding azide and alkyne groups [50].

As we described in the introduction, the phenothiazine-triazole hybrid compounds obtained so far have shown promising biological properties. In our approach to further modify these structures, we changed the phenothiazine ring to a quinobenzothiazine ring.

**Scheme 3:**
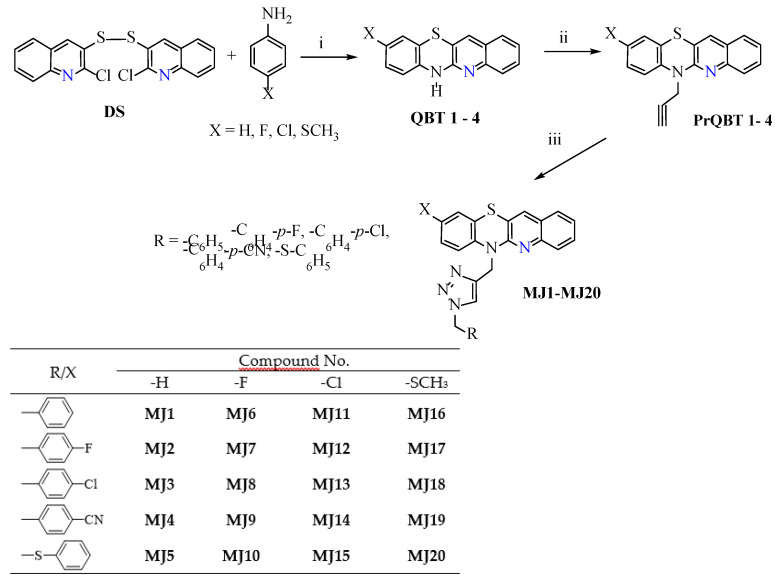
Scheme synthesis of 1,2,3-triazolo-quinobenzothiazine hybrids **MJ1**–**MJ20**. Reagents and reaction conditions: (i) MEDG, reflux, 3 h [51]; (ii) DMF, *t*-BuOK, rt. 1 h, 3-bromo-1-propyne, 24 h [52,53]; (iii) toluene, copper iodide (I), organic azide, 70 °C, 48 h.

Accordingly, we began our research by developing the synthesis of structural analogs of quinobenzothiazines (quino [3,2-b]benzo [1,4]thiazine, 9-chloroquino [3,2-b]benzo [1,4]thiazine, 9-fluoroquino [3,2-b]benzo [1,4]thiazine and 9-methylthiochino [3,2-b]benzo [1,4]thiazine) containing different 1,2,3-triazolyl substituents and to investigate their anticancer activity (Scheme 3).

**Scheme 4:**
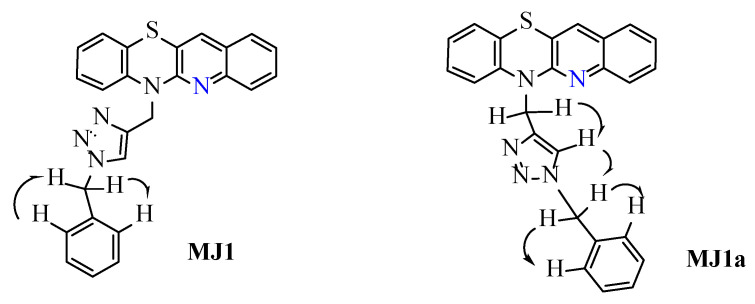
The ROESY experiment for 1,2,3-triazolo-quinobenzothiazine hybrids.

In the next step, we synthesized these 20 derivatives initially evaluated in silico for anticancer activity. Syntheses were started by obtaining appropriate quinobenzothiazine analogs—N*H*-quinobenzothiazines **QBT 1**–**4** according to the literature procedures, which were the starting material for further steps [51]. The N*H*-quinobenzothiazines **QBT 1**–**4** were then transformed in the next step by reaction with 3-bromo-1-propyne into 6-propynyl derivatives **PrQBT 1**–**4** according to the methodology previously developed and described [52,53]. Substituted triazole derivatives of quinobenzothiazine were synthesized using copper-catalyzed azide-alkyne cycloaddition (CuAAC, with selected azides, in the presence of copper catalyst, in toluene). The identification of the product structure was based on ^1^H, ^13^C NMR spectra, 2D NMR experiments: COSY and ROESY and HR MS mass spectrometry. The crude reaction products were separated by column chromatography to obtain pure final derivatives **MJ1**–**MJ20** in good yield. As is known from previous studies, the reaction of 2-propynyl derivatives with organic azides can lead to 1,4- or 1,5- regioisomers [23,27]. To confirm this dependence, the structure of the obtained products was unequivocally confirmed using the 2D NMR ROESY experiment for quinobenzothiazine **MJ1** (Scheme 4). Analyzing the results of this experiment, it can be seen that the protons of the benzyl group were correlated with the aromatic proton of the 1,2,3-triazole ring and the ortho-protons of the benzene ring. However, if the reaction resulted in the formation of the 1,5-regioisomer of the substitution quinobenzothiazine **MJ1a**, a correlation between the CH_2_ group on the nitrogen atom of the thiazine and the CH_2_ group on the triazole ring should be visible. No such correlations were observed in the obtained spectra of quinobenzothiazine **MJ1**.

### 2.2. Prediction of Biological Activity Using the PASS Web Platform

In the next step, compounds were tested in silico. For in silico research using the PASS platform [54] we chose 1,2,3-triazole-quinobenzothiazine hybrids containing benzyl, 4-fluorobenzyl, 4-chlorobenzyl, 4-cyanobenzyl and methylthiophenyl units.

Based on the data obtained from the predictive analysis for the tested substances **MJ1**–**MJ20** (Table 1), conclusions can be drawn about their potential biological properties. Most of the tested compounds show potential activity as anaphylatoxin receptor antagonists, while quinobenzothiazines **MJ1**, **MJ3**, **MJ5**, **MJ6**, **MJ11**, **MJ13**, and **MJ15** show the highest Pa (probability of activity) values in this range. Anaphylatoxins C3a and C5a are polypeptides that are released during complement activation at the site of activation of the inflammatory reaction [55]. This causes the activation of granulocytes, macrophages, and mast cells and their chemotaxis, as well as increased vascular permeability [56]. In addition to immunomodulatory properties, anaphylatoxins regulate tissue regeneration [57]. This property suggests the possibility of modulating inflammatory responses, which may be useful in the treatment of autoimmune diseases or inflammatory conditions. Compounds such as **MJ2**, **MJ16**, **MJ17**, and **MJ19** are highly likely to have a positive effect on the therapy of autoimmune diseases.

Most of the compounds studied (e.g., **MJ1**, **MJ2**, **MJ3**, **MJ6**, **MJ11**, **MJ15**, **MJ20**) have relatively high Pa values as therapeutic agents for atherosclerosis, indicating their possible influence on lipid metabolism or inflammatory processes associated with this disease [58].

Compounds such as **MJ3**, **MJ5**, **MJ6**, **MJ11**, **MJ13**, and **MJ20** have high predictions for glycosylphosphatidylinositol-related phospholipase D inhibitor properties. This feature may be important in the regulation of signaling pathways related to cell membranes and immune responses [59]. The quinobenzothiazines **MJ5**, **MJ6**, **MJ10** and **MJ20** may have properties of the interleukin 2 agonist, suggesting the potential to stimulate the immune response. Such activity may be important in supportive therapy in oncology or infectious diseases [60].

Some compounds (e.g., **MJ4**, **MJ19**) can potentially exhibit neurotransmitter uptake inhibitor properties, suggesting their use in the treatment of neurological disorders such as depression or neurodegenerative diseases [61].

### 2.3. Microscopic Observations

**Figure 1:**
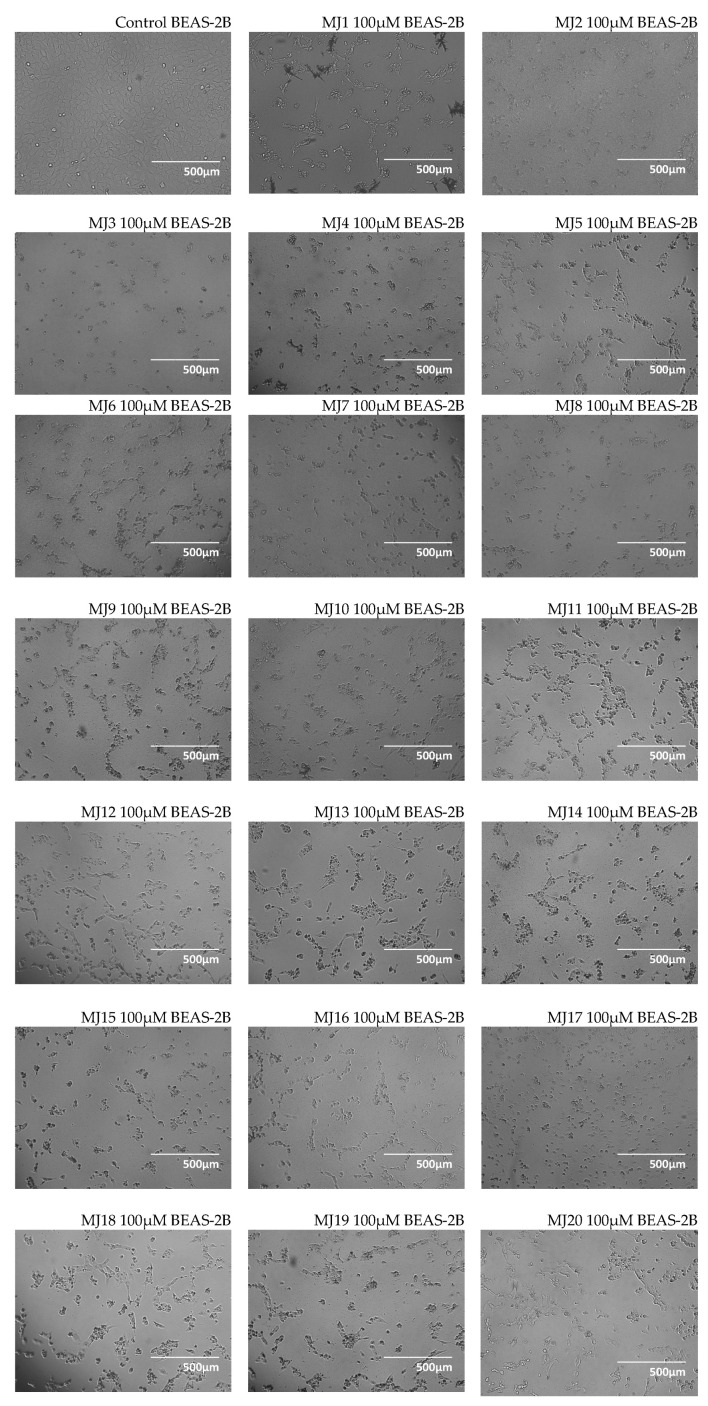
BEAS-2B cells treated with **MJ1**–**MJ20** at 100 μM after 24 h incubation.

**Figure 2:**
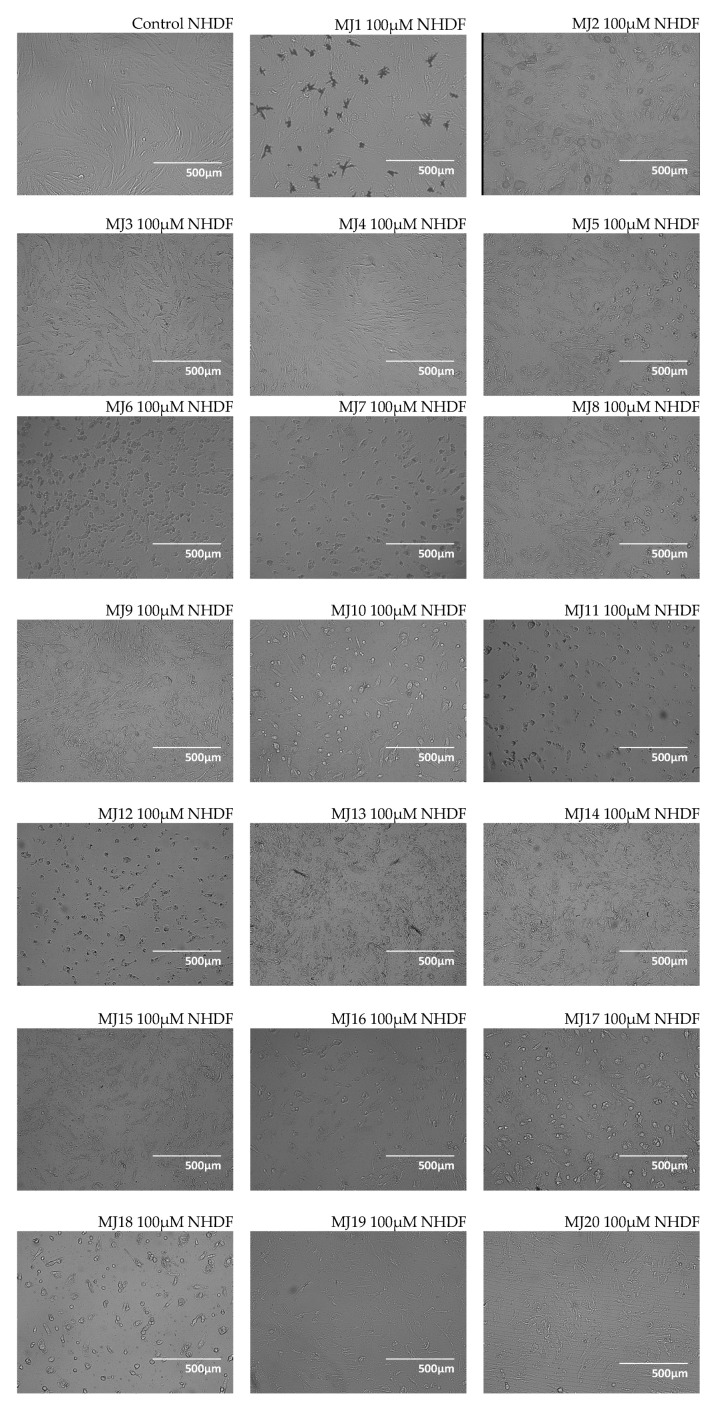
NHDF cells treated with **MJ1**–**MJ20** at 100 μM after 24 h incubation.

**Figure 3:**
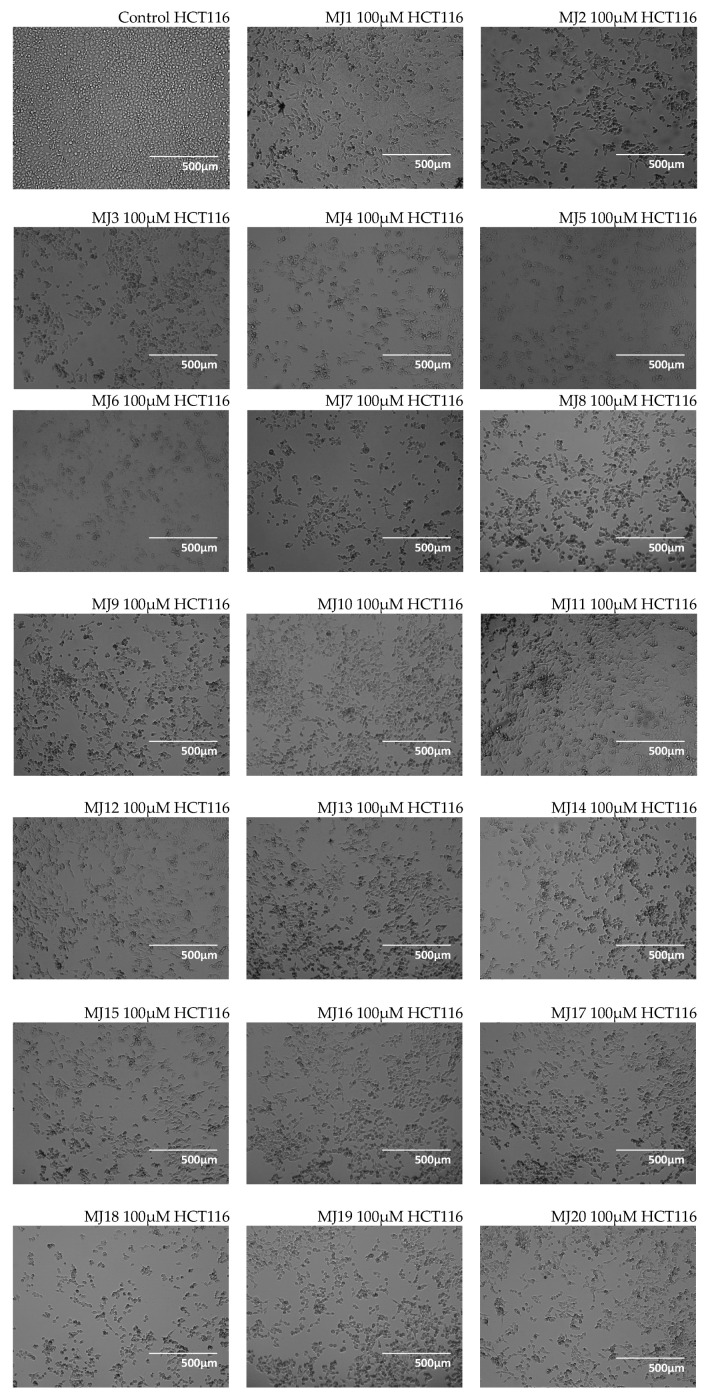
HCT116 cells treated with **MJ1**–**MJ20** at 100 μM after 24 h incubation.

**Figure 4:**
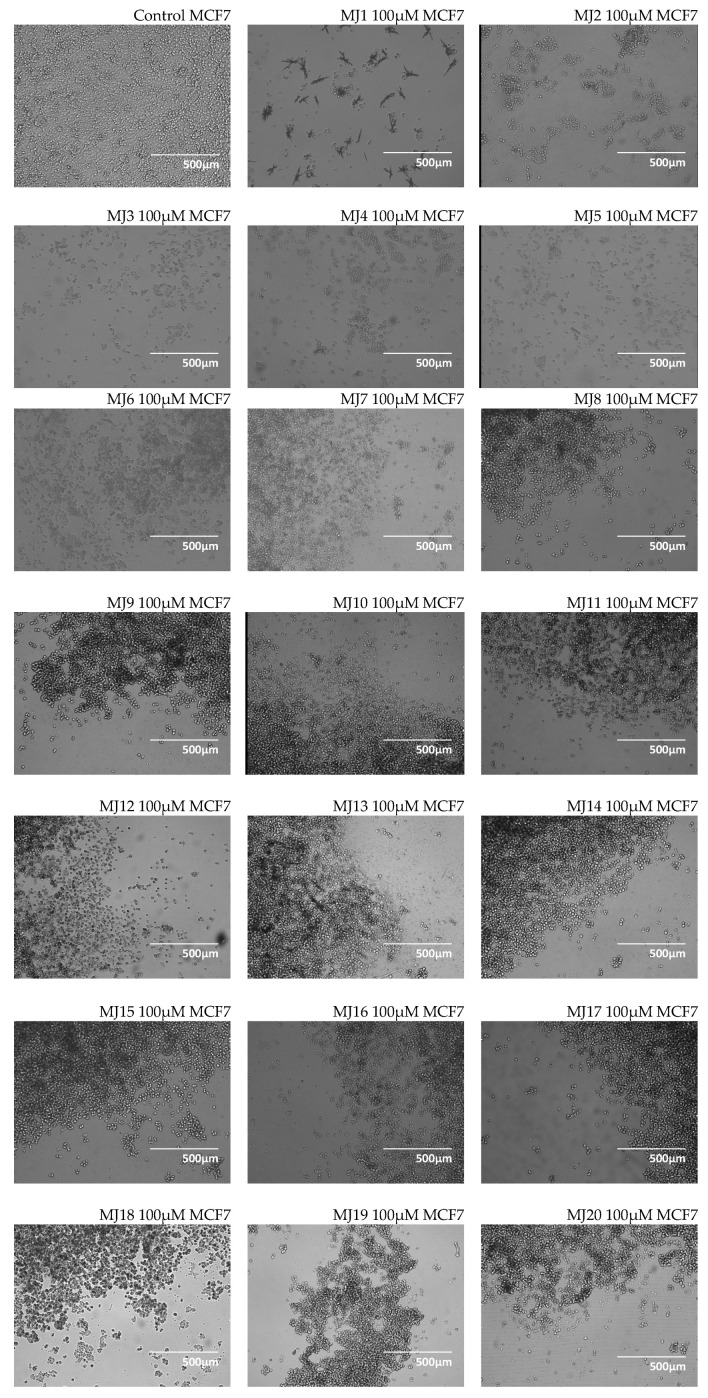
MCF7 cells treated with **MJ1**–**MJ20** at 100 μM after 24 h incubation.

**Figure 5:**
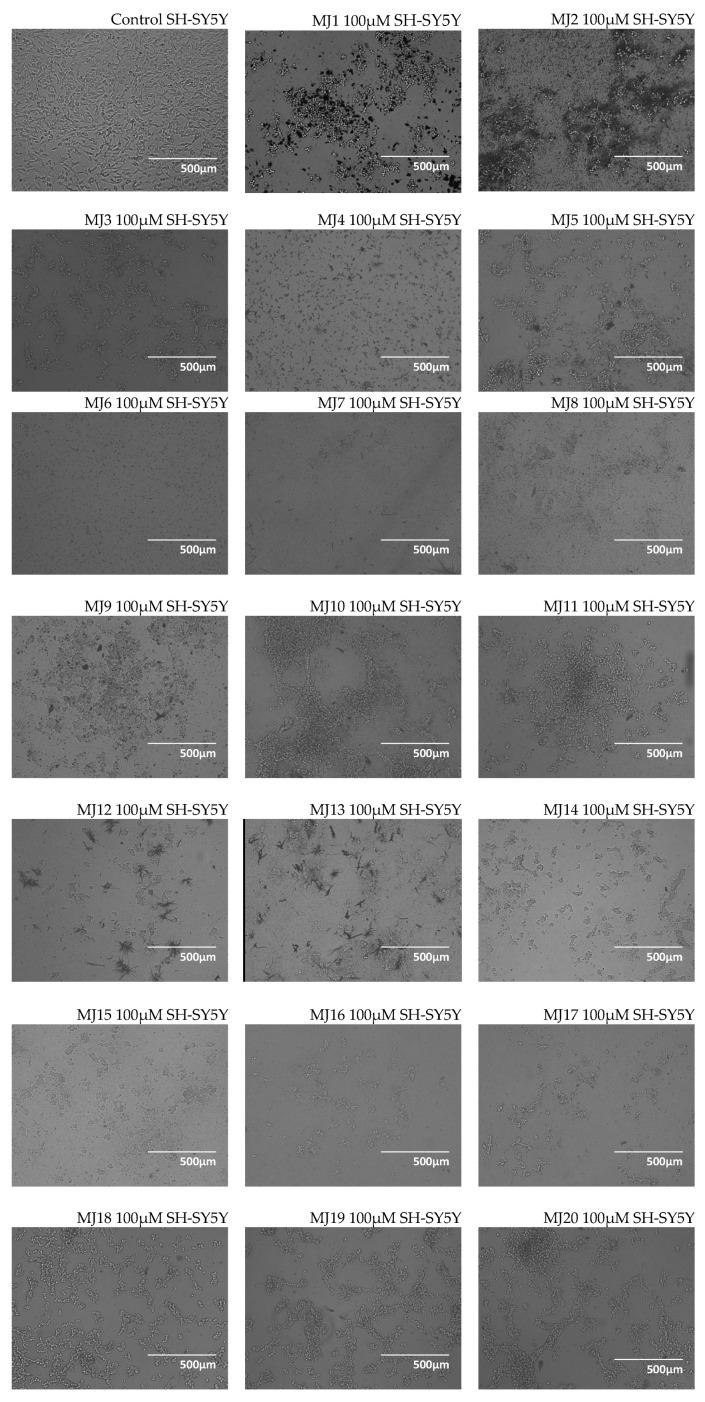
SH-SY5Y cells treated with **MJ1**–**MJ20** at 100 μM after 24 h incubation.

**Figure 6:**
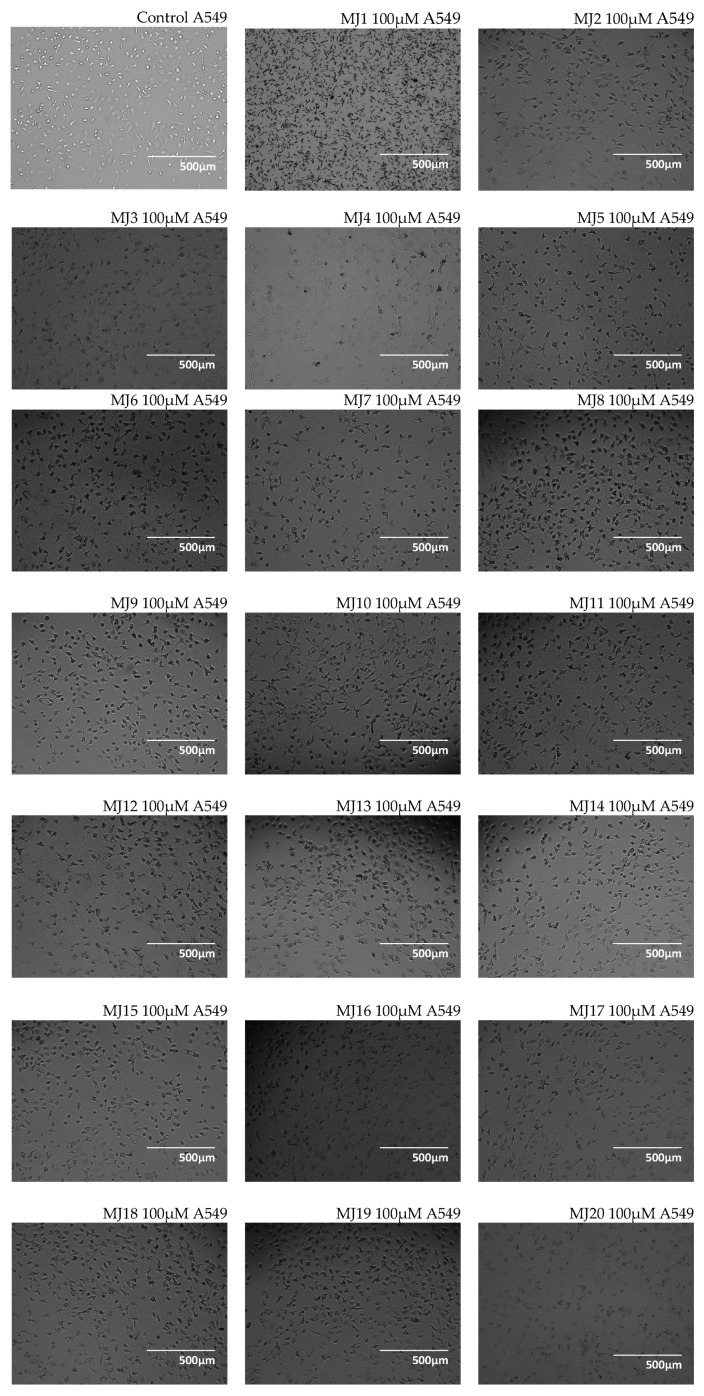
A549 cells treated with **MJ1**–**MJ20** at 100 μM after 24 h incubation.

Microscopic studies were performed using BEAS-2B, NHDF, HCT116, MCF7, SH-SY5Y, A549 cell lines. Examples of BEAS-2B, NHDF, HCT116, MCF7, SH-SY5Y, and A549 cells images after 24 h incubation with **MJ1**–**MJ20** compounds at a concentration of 100 µM are shown in Figure 1, Figure 2, Figure 3, Figure 4, Figure 5 and Figure 6. Additional images showing the cell morphology of these lines in response to compounds **MJ1**–**MJ20** at concentrations of 6.25–100 µM are provided in Supplementary Materials File S2.

Microscopic observations showed that the quinobenzothiazines **MJ1**–**MJ20** affected cell morphology in a concentration-dependent manner. At lower concentrations (6.25–25 µM), the changes were minimal or invisible, and the cells maintained their typical shape and even distribution on the substrate, indicating their moderate effect under these conditions. High concentrations (50–100 µM) caused significant morphological changes, such as detachment of cells from the substrate and their adoption of a spherical shape, which may suggest the initial stages of cell death processes such as apoptosis or necrosis. Cancer lines such as HCT116, MCF7, SH-SY5Y, and A549 showed a greater susceptibility to morphological changes in response to high concentrations of quinobenzothiazines compared to normal cells such as BEAS-2B or NHDF, which may indicate differences in the mechanisms of cell death between cancer and healthy cells. Although images at 100 µM concentration are included in the article, detailed images showing cell morphology in response to lower concentrations of compounds (6.25–50 µM) are provided in the Supplementary Materials (the full range of concentrations the BEAS-2B and HCT116 lines, the 100 µM concentration the NHDF, MCF7, SH-SY5Y, and A549 lines) and provide additional information on morphological changes, allowing for a more complete interpretation of the effects of the compounds tested. These results indicate that the quinobenzothiazines **MJ1**–**MJ20** can affect cell survival and behavior in a concentration-dependent manner, making them interesting candidates for further investigation of their therapeutic potential.

### 2.4. Alamar Blue Test

**Figure 7:**
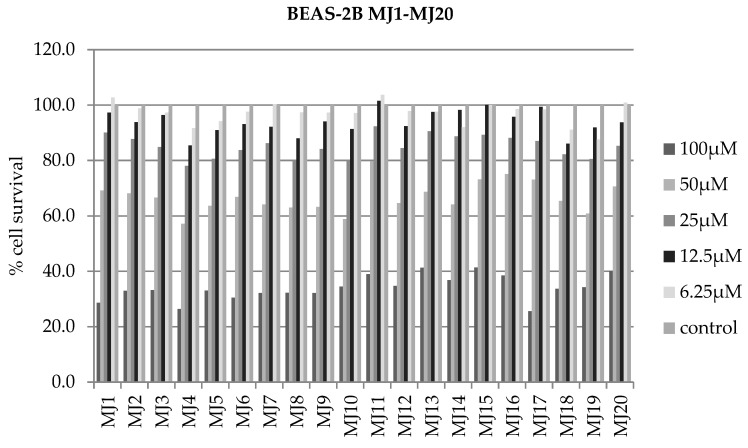
Percentage of BEAS-2B cell survival after incubation with **MJ1**–**MJ20** compounds for 24 h.

**Figure 8:**
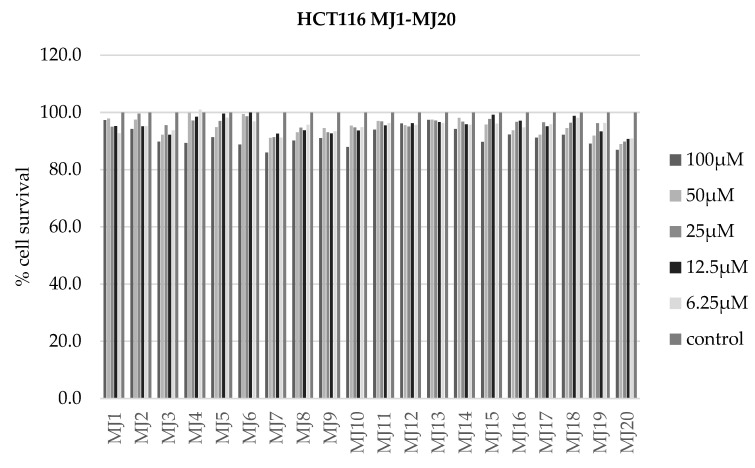
Percentage of HCT116 cell survival after incubation with **MJ1**–**MJ20** compounds for 24 h.

**Figure 9:**
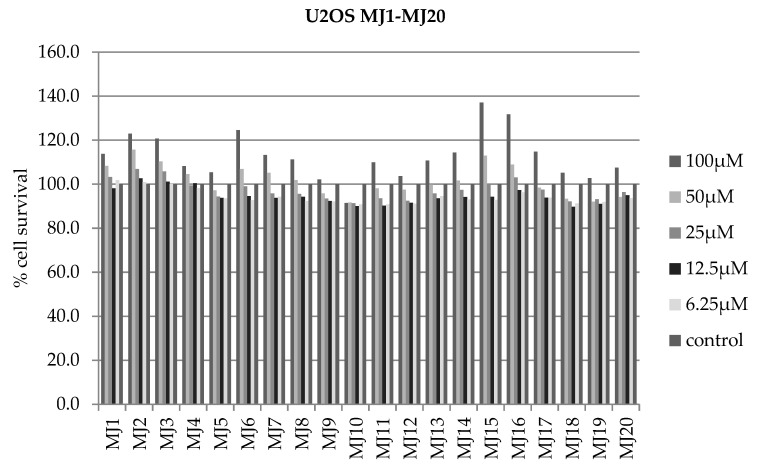
Percentage of U2OS cell survival after incubation with **MJ1**–**MJ20** compounds for 24 h.

**Figure 10:**
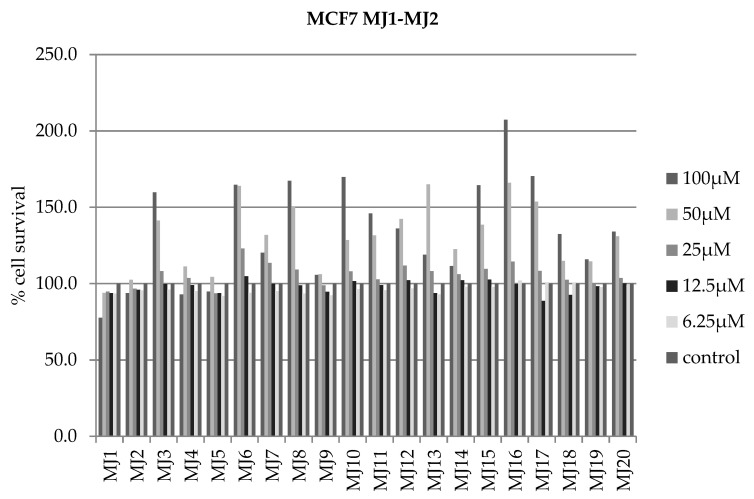
Percentage of MCF7 cell survival after incubation with **MJ1**–**MJ20** compounds for 24 h.

**Figure 11:**
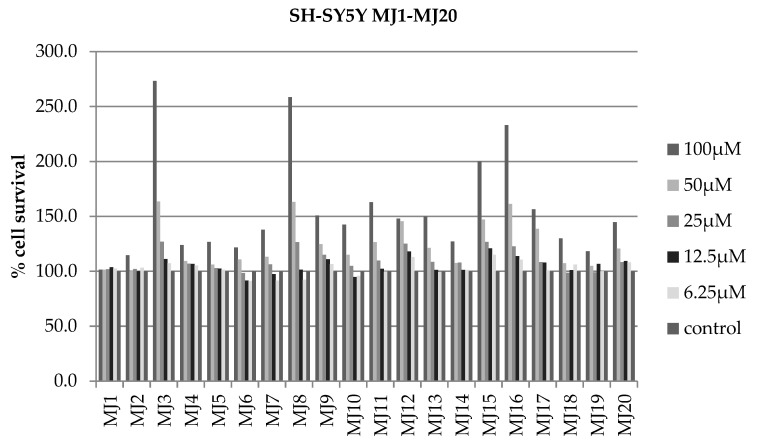
Percentage of SH-SY5Y cell survival after incubation with **MJ1**–**MJ20** compounds for 24 h.

**Figure 12:**
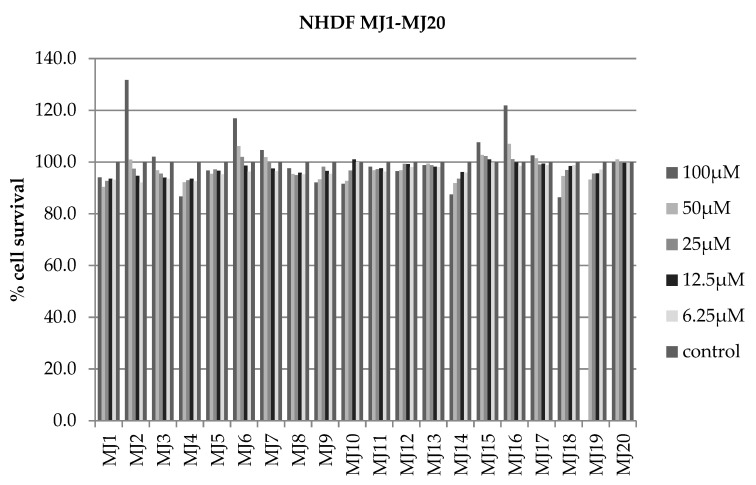
Percentage of survival of NHDF cells after incubation with **MJ1**–**MJ20** compounds for 24 h.

**Figure 13:**
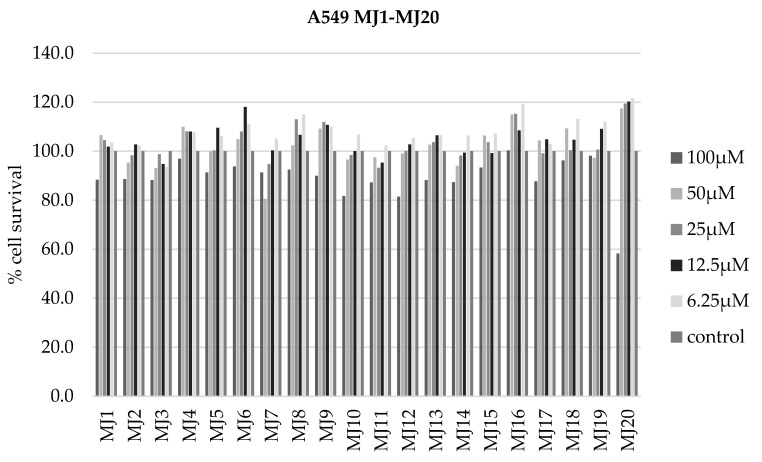
Percentage of A549 cell survival after incubation with **MJ1**–**MJ20** compounds for 24 h.

The cytotoxicity of the tested chemical compounds **MJ1**–**MJ20** was tested using the Alamar Blue test. The experiment was conducted on cell lines BEAS-2B (Figure 7), HCT116 (Figure 8), U2OS (Figure 9), MCF7 (Figure 10), SH-SY5Y (Figure 11), NHDF (Figure 12), and A549 (Figure 13). Cells were seeded in 96-well plates and incubated with the tested compounds for 24 h. The test result is the IC_50_ parameter, which is a measure of cytotoxic activity expressed by the concentration of the substance that causes the death of half of the analyzed cells compared to the control.

For the analyzed compounds, the IC_50_ parameter could only be calculated for the BEAS-2B line. For the remaining cell lines, the percentage of cell survival was so high that it prevented the calculation of IC_50_. The obtained results are summarized in Table 2.

The obtained results indicate that the tested compounds are not good cytostatics for the tested cell lines, as evidenced by the high survival percentages compared to the control after the use of **MJ1**–**MJ20**.

The results of the Alamar Blue test showed that for the BEAS-2B line it was possible to determine the IC_50_ values, but they were high, indicating limited cytotoxicity of the tested quinobenzothiazines against these cells. In the case of cancer lines such as HCT116, A549, MCF7 or SH-SY5Y, the IC_50_ values could not be determined, which suggests their significant resistance to the action of **MJ1**–**MJ20** compounds at the applied concentrations. This phenomenon may result from the activation of cellular resistance mechanisms, such as overexpression of antiapoptotic proteins (e.g., BCL2) or effective drug efflux by transport pumps. Cancer cell lines that demonstrate this resistance may be a valuable model for studying the mechanisms of chemoresistance and assessing potential strategies to overcome it in the context of designing new anticancer therapies.

Alamar Blue test results showed that the highest concentrations of the quinobenzothiazines tested (100 µM) could lead to interference in the reading of the results. Cell viability values exceeding 100% were observed in some samples, suggesting the possibility of interference of the compound with the Alamar Blue reagent itself. This phenomenon could be caused by chemical reactions between the compounds tested and the dye or excessive metabolic activity of the cells in response to high concentrations of the compounds, which could lead to an erroneous increase in the fluorescence signal. These results indicate the need for caution in interpreting data for high concentrations of the compounds.

### 2.5. RT-qPCR

Real-time polymerase chain reaction was performed for the reference gene RPL41 (ribosomal protein L41) and the tested genes: BCL2, AIFM2, MDM2, IL6 and IL8. The RPL41 gene was chosen as a reference gene because it is stable and does not undergo significant changes under different experimental conditions or in different cell types. The BEAS-2B line was selected for the experiment. The expression level of selected genes was measured after 24 h of incubation with the tested substances **MJ1**–**MJ20** at a concentration of 100 μM (Table 3).

#### 2.5.1. BEAS-2B’s BCL2 Gene Expression

**Figure 14:**
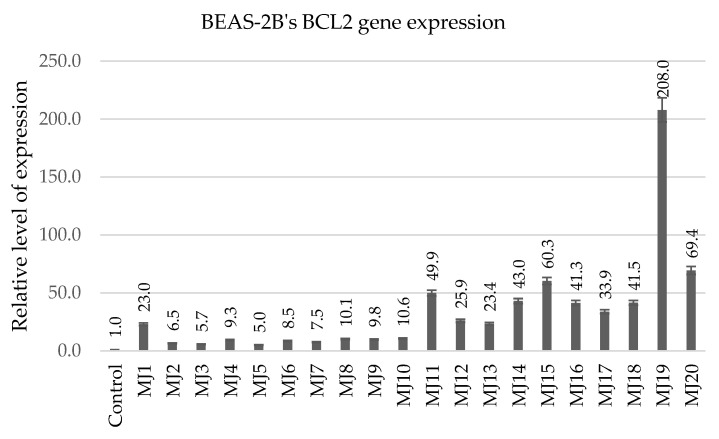
Relative expression level of the BCL2 gene for compounds **MJ1**–**MJ20** at a concentration of 100 μM and 24 h of incubation in BEAS-2B cell line. The results are presented as mean ± SD, calculated from three experiments and compared with the untreated controls.

The RT-qPCR experiment was performed for the BEAS-2B cell line. In this experiment, the BCL2 gene was studied, and RPL41 was used as the reference gene (Figure 14).

BCL2 is a gene encoding an antiapoptotic protein that inhibits the process of programmed cell death. An increase in its expression was observed in response to most of the compounds tested, with the highest induction observed for **MJ19** (208-fold increase) and **MJ20** (69.4-fold increase). These results indicate that these quinobenzothiazines can enhance cell survival mechanisms. In the context of anticancer therapy, such activity is undesirable because it may promote the survival of cancer cells, especially under conditions of oxidative stress or exposure to cytotoxic drugs. However, in degenerative or inflammatory diseases, increased BCL2 may help protect healthy cells from damage.

#### 2.5.2. BEAS-2B’s MDM2 Gene Expression

**Figure 15:**
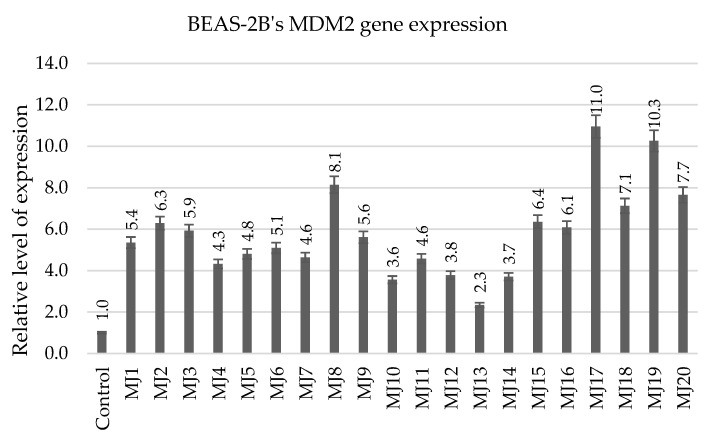
Relative expression level of the MDM2 gene for compounds **MJ1**–**MJ20** at 100 μM concentration and 24 h incubation in BEAS-2B cell line. The results are presented as mean ± SD, calculated from three experiments, and compared with untreated controls.

The RT-qPCR experiment was performed for the BEAS-2B cell line. In this experiment, the MDM2 gene was studied and RPL41 was used as reference gene (Figure 15).

MDM2 is a key regulator of p53, which plays an important role in DNA damage response and cell cycle regulation. The high expression of MDM2 in response to **MJ17** (11.0-fold increase) and **MJ19** (10.3-fold increase) suggests the ability of these compounds to modulate cell survival mechanisms. Similarly to BCL2, this may be beneficial in the context of protecting healthy cells.

#### 2.5.3. BEAS-2B’s AIFM2 Gene Expression

**Figure 16:**
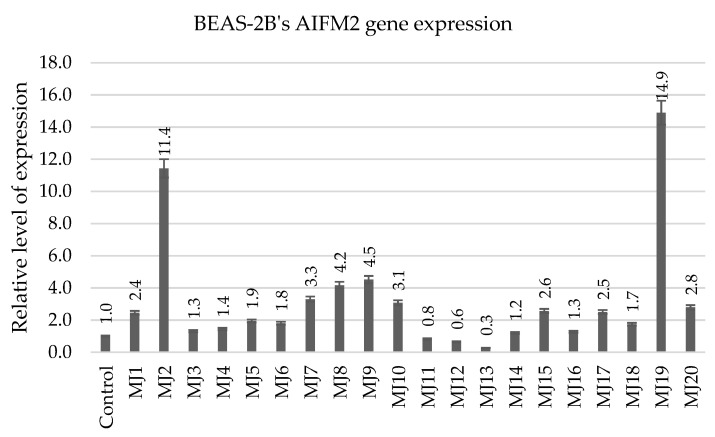
Relative expression level of the AIFM2 gene for compounds **MJ1**–**MJ20** at 100 μM concentration and 24 h incubation in BEAS-2B cell line. The results are presented as mean ± SD, calculated from three experiments, and compared with untreated controls.

The RT-qPCR experiment was performed for the BEAS-2B cell line. In this experiment, the AIFM2 gene was studied and RPL41 was used as reference gene (Figure 16).

Analysis of AIFM2 gene expression showed a significant increase in its level in BEAS-2B cells after the application of **MJ2** (11.4-fold increase) and **MJ19** (14.9-fold increase) compounds compared to the control, indicating the activation of protective mechanisms against ferroptosis. AIFM2 plays a key role in the regulation of ferroptosis, a form of cell death associated with lipid peroxidation and the accumulation of reactive oxygen species (ROS) in the presence of iron. Increased expression of AIFM2 in BEAS-2B cells after the application of **MJ2** and **MJ19** may suggest that the tested compound induces moderate oxidative stress, which stimulates adaptive mechanisms, such as the activation of antiferroptotic pathways. The high expression of AIFM2 may play a key role in the protection of BEAS-2B cells against ferroptotic death, which emphasizes the potential of **MJ2** and **MJ19** in the study of diseases in which the regulation of ferroptosis may be beneficial, such as neurodegenerative or inflammatory diseases.

#### 2.5.4. BEAS-2B’s IL6 and IL8 Gene Expression

**Figure 17:**
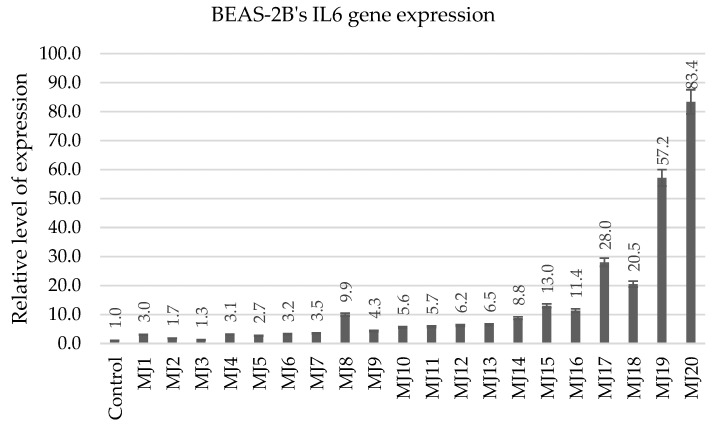
Relative expression level of IL6 gene for compounds **MJ1**–**MJ20** at 100 μM concentration and 24 h incubation in BEAS-2B cell line. The results are presented as mean ± SD, calculated from three experiments, and compared with untreated controls.

**Figure 18:**
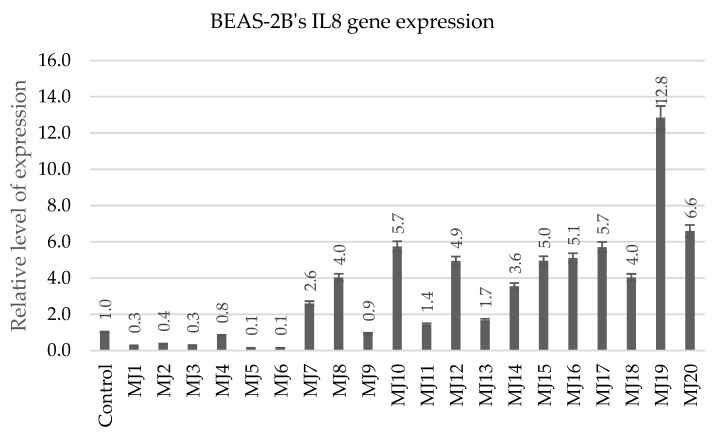
Relative expression level of the IL8 gene for compounds **MJ1**–**MJ20** at 100 μM concentration and 24 h incubation in BEAS-2B cell line. The results are presented as mean ± SD, calculated from three experiments, and compared with untreated controls.

The RT-qPCR experiment was performed for the BEAS-2B cell line. In this experiment, the IL6 and IL8 genes were studied and RPL41 was used as a reference gene (Figure 17 and Figure 18).

The quinobenzothiazines **MJ19** and **MJ20** showed a strong ability to induce pro-inflammatory cytokines. In case of IL6, the highest expression was noted for **MJ20** (83.4-fold increase), while **MJ19** induced an increase of 57.2-fold. A similar trend was observed for IL8, where **MJ19** induced an increase of 12.85 and **MJ20** of 6.6. These interleukins play a key role in the regulation of inflammatory responses. Their induction may indicate the ability of quinobenzothiazines to modulate the immune system, making them potential candidates for adjuvant therapies in inflammatory and autoimmune diseases.

The quinobenzothiazines obtained in the present study differ in their 9-position substituents and in their 1,2,3-triazole ring substituents. **MJ1**–**MJ5** quinobenzothiazines do not have a substituent in position 9; **MJ6**–**MJ10** quinobenzothiazines are substituted with fluorine in this position; and **MJ11**–**MJ15** quinobenzothiazines are substituted with a chlorine atom. The last group, quinobenzothiazines **MJ16**–**MJ20**, have a thiomethyl substituent in position 9. The results demonstrate how different substituents impact the biological activity of the compounds, particularly with respect to gene expression. **MJ19** and **MJ20** compounds, which contain a sulfur-containing thiomethyl substituent in position 9 of the quinobenzothiazine system and a methylthiophenyl substituent in the triazole ring (**MJ20** only), exhibited enhanced activity in modulating BCL2, MDM2, and pro-inflammatory cytokine gene expression levels. This enhanced activity can be attributed to the electron-donating nature of the sulfur substituents, which potentially increases the compounds’ affinity for biological targets that regulate gene expression pathways. Additionally, these derivatives lack halogen atoms in the phenyl ring of the triazole substituent, demonstrating a negative inductive effect. The compounds also differed in the type of substituents in the triazole ring, including benzyl, 4-halobenzyl, 4-cyanobenzyl or phenylthiomethyl groups. It has been shown that compounds containing 4-halobenzyl substituents (e.g., **MJ2**, **MJ3**, **MJ7**, **MJ8**) can show moderate activity, but did not achieve as high gene induction as compounds with a thiomethyl group. Substitution of the phenyl ring with fluorine or chlorine atoms increases bond polarity and can affect the lipophilicity of molecules, which is important for cell membrane permeability. However, the presence of halogens may also have an inductive effect that decreases the activity of certain signaling pathways, as observed in the lower expression levels of IL6 and IL8 for these derivatives compared to **MJ19** and **MJ20**.

#### 2.5.5. HCT116′s BCL2 Gene Expression

In each of the four compound groups: **MJ1**–**MJ5**, **MJ6**–**MJ10**, **MJ11**–**MJ15**, and **MJ16**–**MJ20**, one compound was selected based on the best IC_50_ value and a distinctive gene expression profile (BCL2, MDM2, AIFM2, IL6, IL8). The comparison is presented in Table 3. Based on these data, four of the most promising compounds (**MJ2**; **MJ8**; **MJ15**; **MJ19**)- one from each group—were selected for further studies in the HCT116 tumor cell line.

**Table 3:** Summary of the most promising **MJ1**–**MJ20** compounds from each chemical group based on their biological activity profile in BEAS-2B cells (IC_50_ and relative level of gene expression).

	MJ1–MJ5	MJ6–MJ10	MJ11–MJ15	MJ16–MJ20
**IC_50_**	**MJ4** (64.526 µM)	**MJ8** (71.858 µM)	**MJ12** (75.636 µM)	**MJ17** (72.595 µM)
**BCL2**	**MJ1** (23.0)	**MJ10** (10.6)	**MJ15** (60.3)	**MJ19** (208.0)
**MDM2**	**MJ2** (6.3)	**MJ8** (8.1)	**MJ15** (6.4)	**MJ17** (11.0)
**AIFM2**	**MJ2** (11.4)	**MJ9** (4.5)	**MJ15** (2.6)	**MJ19** (14.9)
**IL6**	**MJ1** (3.0)	**MJ8** (9.9)	**MJ15** (13.0)	**MJ20** (83.4)
**IL8**	**MJ4** (0.8)	**MJ10** (5.7)	**MJ15** (5.0)	**MJ19** (12.8)

**Figure 19:**
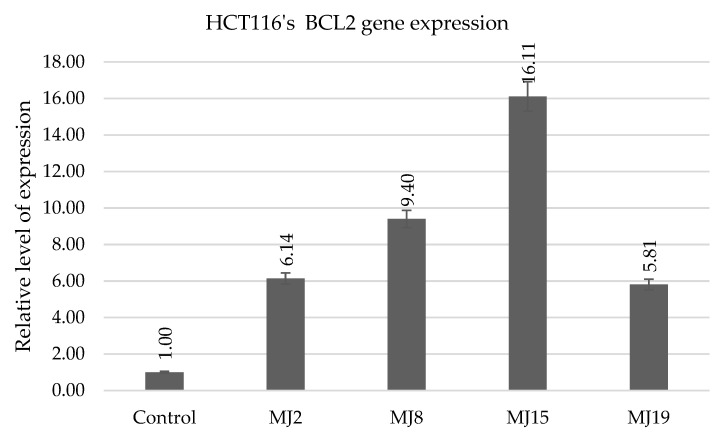
Relative expression level of the BCL2 gene for compounds **MJ2**, **MJ8**, **MJ15**, **MJ19** at 100 μM concentration and 24 h incubation in HCT116 cell line. The results are presented as mean ± SD, calculated from three experiments, and compared with untreated controls.

The results of the analysis of BCL2 gene expression levels in the HCT116 cancer line for compounds **MJ2**, **MJ8**, **MJ15**, and **MJ19** indicate an increase compared to the control, suggesting that the tested compounds may also influence the regulation of mechanisms related to cell survival in cancer cells (Figure 19). Of particular note, although BCL2 expression levels in HCT116 were moderately elevated (from 5.81 to 16.11), they exhibited a clear modulatory effect, which may indicate a potential protective or adaptive effect under conditions of oxidative stress. An even more significant effect was observed in the healthy BEAS-2B lines, in which BCL2 expression was significantly higher, particularly for **MJ15** (60.3) and MJ19 (208.0). This relationship may indicate a selective protective effect of these compounds on normal cells, while having a limited effect on cancer cells, but this requires further study. The observed effects support the hypothesis of a favorable action profile of the tested compounds as molecules capable of modulating survival mechanisms in a manner dependent on the cell type and its biological state.

#### 2.5.6. HCT116′s MDM2 Gene Expression

**Figure 20:**
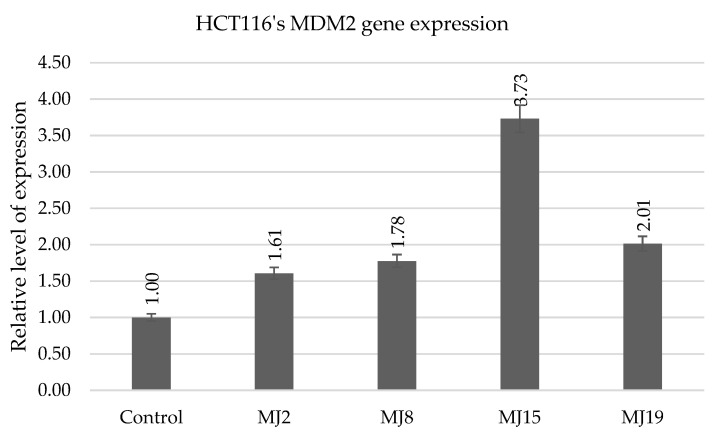
Relative expression level of the MDM2 gene for compounds **MJ2**, **MJ8**, **MJ15**, **MJ19** at 100 μM concentration and 24 h incubation in HCT116 cell line. The results are presented as mean ± SD, calculated from three experiments, and compared with untreated controls.

The results of MDM2 gene expression in the HCT116 cancer line after treatment with **MJ2**, **MJ8**, **MJ15**, and **MJ19** compounds showed a small but noticeable increase compared to the control, with the highest value for **MJ15** (3.73), which may suggest that some of the tested compound derivatives are capable of mildly modulating the p53-MDM2 regulatory axis in cancer cells (Figure 20). On the other hand, significantly higher levels of MDM2 expression were observed in healthy BEAS-2B cells, with values ranging from 6.3 for **MJ2** to as much as 10.3 for **MJ19**. This significant difference may indicate a selective effect of these compounds, which manifests itself in a stronger increase in MDM2 in normal than cancer cells. Because MDM2 is a key inhibitor of p53, its increased expression in healthy cells may provide physiological protection against excessive activation of cellular stress and apoptotic pathways. Under the influence of bioactive compounds, this may be a homeostatic mechanism that promotes the survival of healthy cells. In cancer cells, however, a moderate increase in MDM2 levels may be considered therapeutically beneficial because it does not lead to a strong inhibition of p53 function, which could potentially reduce treatment efficacy. Mild induction of MDM2 may therefore play a regulatory role without inducing severe resistance to apoptosis.

#### 2.5.7. HCT116 AIFM2

**Figure 21:**
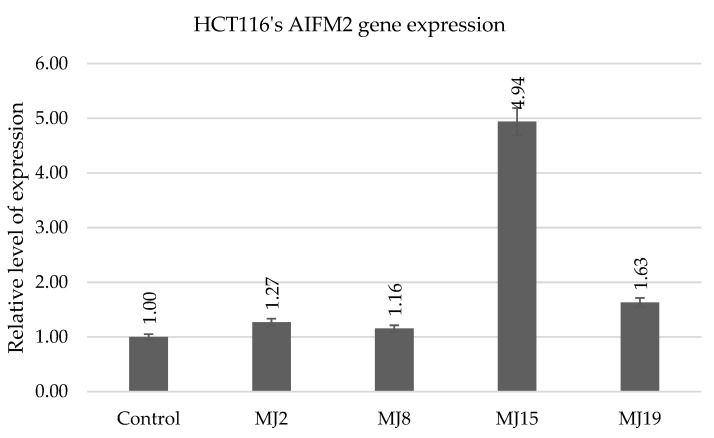
Relative expression level of the AIFM gene for compounds **MJ2**, **MJ8**, **MJ15**, **MJ19** at 100 μM concentration and 24 h incubation in HCT116 cell line. The results are presented as mean ± SD, calculated from three experiments, and compared with untreated controls.

Analysis of AIFM2 gene expression levels in the HCT116 cancer line after treatment with **MJ2**, **MJ8**, **MJ15**, and **MJ19** compounds revealed a slight increase in expression compared to the control for **MJ2**, **MJ8**, and **MJ19** compounds, and a moderate increase compared to the control, particularly for **MJ15**, which reached a relative value of 4.94 (Figure 21). This result may indicate the ability of some of the tested compounds, especially **MJ15**, to activate protective mechanisms related to redox balance and prevent ferroptosis in cancer cells. However, the most significant difference was observed in AIFM2 expression in healthy BEAS-2B cells, where the values were significantly higher—up to 14.9 for **MJ19** and 11.4 for **MJ2**. This suggests that the tested compounds can selectively and more effectively induce AIFM2 expression in normal cells than in cancer cells, which may be a significant advantage in the context of their potential use as protective agents, which requires further research. AIFM2, known as an important suppressor of ferroptosis, protects cells from oxidative stress and lipid peroxidation, so its activation may indicate the ability of phenothiazines to enhance the natural defense mechanisms of healthy cells. At the same time, moderate induction of AIFM2 in cancer cells may be beneficial because it does not block potentially effective pathways leading to cancer cell death, such as ferroptosis.

#### 2.5.8. HCT116′s IL6 andIL8 Gene Expression

**Figure 22:**
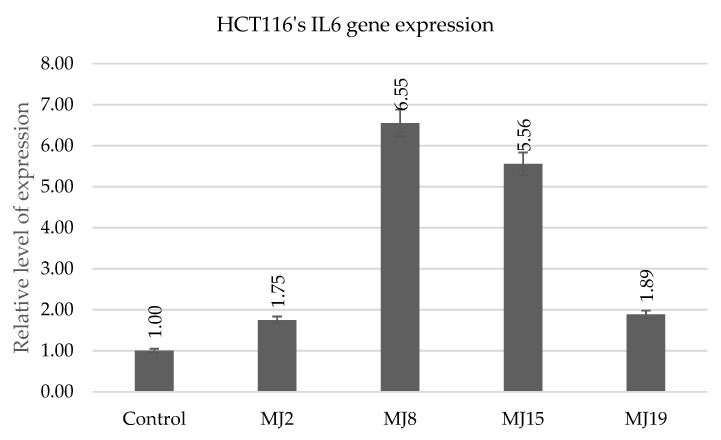
Relative expression level of the IL6 gene for compounds **MJ2**, **MJ8**, **MJ15**, **MJ19** at 100 μM concentration and 24 h incubation in HCT116 cell line. The results are presented as mean ± SD, calculated from three experiments, and compared with untreated controls.

**Figure 23:**
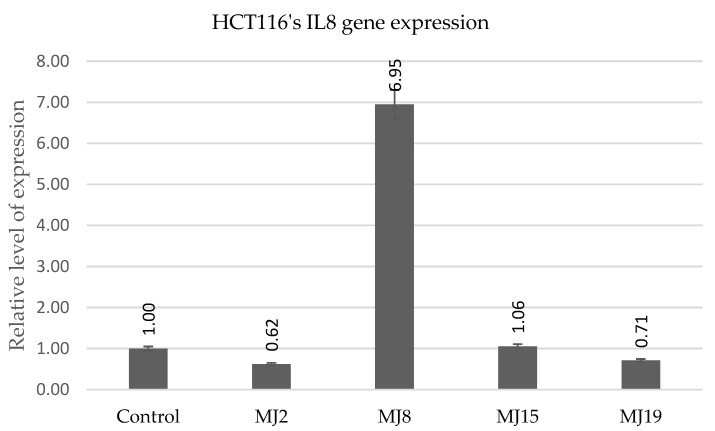
Relative expression level of the IL8 gene for compounds **MJ2**, **MJ8**, **MJ15**, **MJ19** at 100 μM concentration and 24 h incubation in HCT116 cell line. The results are presented as mean ± SD, calculated from three experiments, and compared with untreated controls.

Analysis of IL6 and IL8 gene expression following the administration of **MJ2**, **MJ8**, **MJ15**, and **MJ19** compounds demonstrates that these phenothiazines have the ability to modulate the inflammatory response in a manner that is dependent on both the compound type and the cell type (Figure 22 and Figure 23). A moderate increase in IL6 expression was observed in HCT116 cancer cells, particularly after the administration of **MJ8** (6.55) and **MJ15** (5.56), suggesting activation of a local inflammatory response in the tumor microenvironment. At the same time, IL6 expression was low for **MJ2** and **MJ19**, indicating a more subtle effect of these compounds on pro-inflammatory pathways in cancer cells. In the normal BEAS-2B cell line, IL6 expression values were significantly higher, especially for **MJ19**, which reached a level of 57.2, suggesting its strong potential to activate the immune system in non-neoplastic cells. A similar pattern was observed for IL8, an interleukin that plays a key role in chemotaxis and angiogenesis. In the HCT116 cell line, only **MJ8** significantly increased IL8 expression (6.95), while the remaining compounds induced only a slight or even decreased expression of this gene. In BEAS-2B cells, a significant increase in IL8 expression was observed after treatment with **MJ15** and **MJ19**, with **MJ19** reaching a value as high as 12.85, which may indicate its ability to strongly activate the inflammatory response in healthy cells. This feature could be exploited to enhance anti-infective defenses or to treat conditions where activation of a local inflammatory response is desirable. These results suggest the immunomodulatory potential of the tested compounds and indicate their selective, cell-type-dependent action.

### 2.6. DAPI Staining for Nuclear Morphology Assessment and Apoptosis Observation

During microscopic observations after the application of selected compounds **MJ2**, **MJ8**, **MJ15**, and **MJ19** at a concentration of 100 μM and 24 h of incubation, no significant apoptotic changes were observed in the nuclei of BEAS-2B and HCT116 cells, which is consistent with the increased expression of antiapoptotic genes such as BCL2 compared to the control. Example images of cell nuclei are presented in Supplementary Materials File S2. No significant level of apoptotic bodies was detected.

## 3. Materials and Methods

Melting points were determined on a Boetius apparatus in open capillaries. ^1^H NMR spectra were recorded on a Bruker Avance spectrometer (Bruker, Billerica, MA, USA) at 600 MHz, and ^13^C NMR and ^19^F NMR spectra were recorded on the same spectrometer at 150 MHz and 564 MHz, respectively. CDCl_3_ was used as a solvent. Two-dimensional COSY, NOESY, HSQC, and HMBC spectra of selected compounds were recorded on a Bruker Avance spectrometer at 600 MHz, using CO-SYGPSW, NOESYGPPHSW, HSQCGPPH, and HMBCGP experiments. EI HRMS spectra were recorded on a Brucker Impact II spectrometer (Bruker, Billerica, MA, USA). The ^1^H NMR, ^13^C NMR, and HRMS spectra are provided in Supplementary Materials File S1. Thin layer chromatography was performed on alumina 60 F_254_ neutral (type E) (Merck 1.05581) with CH_2_Cl_2_ as an eluent. 6*H*-Quino [3,2-b]benzo [1,4]thiazine **QBT1**, 6*H*-9-chloroquino [3,2-b]benzo-[1,4]thiazine **QBT2**, 6*H*-9-fluoroquino [3,2-b]benzo [1,4]thiazine **QBT3**, and 6*H*-9-methyltioquino [3,2-b]benzo [1,4] thiazine **QBT4** were obtained according to previously described methods [51]. These quinobenzothiazines were then converted to their 6-propynyl derivatives **PrQBT1**–**PrQBT4** according to previously described procedures [52].

### 3.1. In Silico Study

The biological properties of the analyzed compounds were predicted using the PASS web platform. PASS enables the prediction of the potential biological activities of chemical compounds based on their structure, assuming that biological activity results from the molecular structure of the substance. The tool provides predictions for specific biological properties expressed as the ratio of “probability of activity (Pa)” to the “probability of inactivity (Pi)”. The higher the Pa value, the higher the probability that a given compound exhibits a given activity [62].

### 3.2. General Procedure for the Synthesis of Compounds **MJ1**–**MJ20**

To a solution of 6-propynyl quinobenzothiazine **PrQBT 1**–**4** (0.5 mmol) and copper iodide (I) (0.006 mg) in dry toluene (10 mL), a corresponding organic azide (0.510 mmol) was added. The reaction mixture was heated at 70 °C and stirred for 48 h. The mixture was then cooled, and toluene was distilled off on a vacuum evaporator. The residue was dissolved in CH_2_Cl_2_ and purified by column chromatography (silica gel, CH_2_Cl_2_ as eluent) to obtain pure triazole derivatives **MJ1**–**MJ20**:

[(1-Benzyl-1*H*-1,2,3-triazol-4-yl)-methyl]-quino[3,2-b]benzo[1,4]thiazine (**MJ1**):

Yield: 80% M.p.: 165–166 °C. ^1^H NMR (600 MHz, CDCl_3_) δ: 5.38 (s, 2H, CH_2_), 5.56 (s, 2H, N-CH_2_), 6.88 (t, 1H, H-9, *J* = 7.8 Hz), 7.00 (dd, 1H, H-7, J = 7.8 Hz, J = 1.8 Hz), 7.07–7.09 (m, 2H, 2H-Ph), 7.25–7.19 (m, 5H, 3H-Ph, H-8, H-10), 7.28 (d 1H, H-2, *J* = 8.2 Hz), 7.42–7.46 (m, 2H, H-1, H-3), 7.60 (s, 1H, H-12), 7.78 (m, 1H, H-4), 7.80 (s, 1H, CH). ^13^C NMR (150 MHz, CDCl_3_) δ: 43.94, 54.08, 117.33, 119.77, 120.40, 123.17, 123.86, 124.78, 125.36, 125.69, 126.63, 127.81, 128.16, 128.58, 129.01, 129.22, 130.02, 132.62, 133.01, 134.68, 140.68, 144.36, 151.49. HR MS (ESI) calcd for: C_25_H_20_N_5_S [M + H]^+^: 422.1439, found: 422.1430.

6-((1-(4-Fluorobenzyl)-1*H*-1,2,3-triazol-4-yl)methyl)-quino[3,2-b]benzo[1,4]thiazine (**MJ2**):

Yield: 82% M.p.: 145–146 °C. ^1^H NMR (600 MHz, CDCl_3_) δ: 5.44 (s, 2H, CH_2_), 5.53 (s, 2H, N-CH_2_), 6.93 (t, 1H, H-9, *J* = 7.2 Hz), 6.99–7.03 (m, 2H, H-Ph), 7.08 (d, 1H, H-7, *J* = 7.8 Hz), 7.14 (t, 1H, H-8, *J* = 15.6 Hz), 7.19–7.21 (m, 2H, H-Ph), 7.32 (t, 1H, H-2, *J* = 15 Hz), 7.38 (d, 1H, H-10, *J* = 7.8 Hz), 7.51 (t, 1H, H-3, *J* = 8.4 Hz), 7.55 (d, 1H, H-1, *J* = 7.8 Hz), 7.55–7.7.64 (m, 1H, H-4), 7.66 (s, 1H, H-12), 7.74 (s, 1H, CH). ^13^C NMR (150 MHz, CDCl_3_) δ: 44.10, 53.33, 115.98 (*J* = 22.5 Hz), 117.41, 120.01, 120.53, 122.70, 124.08, 124.78, 125.29, 125.60, 126.41, 126.70, 128.22, 129.70, 129.81 (*J* = 9 Hz), 130.21, 130.54 (*J* = 3 Hz), 133.29, 140.81, 144.23, 151.36, 162.72 (*J* = 246 Hz). ^19^F NMR (564 MHz, CDCl_3_) δ: -112.71. HR MS (ESI) calcd for: C_25_H_19_FN_5_S [M + H]^+^: 440.1345, found: 440.1350.

6-[(1-(4-Chlorobenzyl)-1*H*-1,2,3-triazol-4-yl)methyl]-quino[3,2-b]benzo[1,4]thiazine (**MJ3**):

Yield: 80% M.p.: 165–166 °C. ^1^H NMR (600 MHz, CDCl_3_) δ: 5.32 (s, 2H, CH_2_), 5.45 (s, 2H, N-CH_2_), 6.83 (t, 1H, H-9, *J* = 15.6 Hz), 6.97 (dd, 1H, H-7, *J* = 9 Hz, 1.5 Hz), 7.01 (d, 2H, H-Ph, *J* = 8.5 Hz), 7.03 (t, 1H, H-8, *J* = 8.8 Hz), 7.16 (d, 2H, H-Ph, *J* = 8.5 Hz), 7.20 (t, 1H, H-2, *J* = 15.9 Hz), 7.26 (d, 1H, H-10, *J* = 9 Hz), 7.40–7.43 (m, 2H, H-1, H-3), 7.54 (s, 1H, H-12), 7.58 (d, 1H, H-4, *J* = 7 Hz), 7.69 (s, 1H, CH). ^13^C NMR (150 MHz, CDCl_3_) δ: 43.35, 53.30, 116.88, 119.11, 120.04, 123.40, 124.61, 124.74, 125.91, 126.13, 126.34, 126.53, 128.03, 129.21, 129.29, 129.33, 129.62, 132.37, 133.23, 134.59, 141.04, 145.18, 151.61. HR MS (ESI) calcd for: C_25_H_19_ClN_5_S [M + H]^+^: 456.1050, found: 456.1046.

4-((4-((Quino[3,2-b]benzo[1,4]thiazin-6-yl)methyl)-1*H*-1,2,3-triazol-1-yl)methyl)benzonitrile (**MJ4**):

Yield: 80% M.p.: 165–166 °C. ^1^H NMR (600 MHz, CDCl_3_) δ: 5.56 (s, 2H, CH_2_), 5.71 (s, 2H, N-CH_2_), 7.01–7.05 (m, 2H, H-7, H-9), 7.22 (d, 2H, Ph, *J* = 8.3 Hz), 7.33 (d, 1H, H-2, *J* = 1.8 Hz), 7.41 (t, 1H, H-8, *J* = 7.1 Hz), 7.56–7.60 (m, 5H, H-1, H-3, H-10, 2H-Ph), 7.77 (s, 1H, H-12), 7.97–7.98 (m, 2H, H-4, H-CH). ^13^C NMR (150 MHz, CDCl_3_) δ: 46.72, 53.39, 112.66, 118.08, 119.49, 121.18, 124.71, 124.83, 126.02, 126.36, 126.84, 127.61, 128.00, 128.04, 128.15, 128.30, 132.74, 132.81, 132.83, 134.88, 137.13, 139.73, 141.19, 150.33. HR MS (ESI) calcd for: C_26_H_19_N_6_S [M + H]^+^: 447.1393, found: 447.1386.

6-((1-((Phenylthio)methyl)-1*H*-1,2,3-triazol-4-yl)methyl)-quino[3,2-b]benzo[1,4]thiazine (**MJ5**):

Yield: 80% M.p.: 113–114 °C. ^1^H NMR (600 MHz, CDCl_3_) δ: 5.44 (s, 2H, CH_2_), 5.46 (s, 2H, NCH_2_), 6.85 (t, 1H, H-9, *J* = 7.6 Hz), 6.96–6.99 (m, 3H, H-Ph, H-7, H-10), 7.03–7.06 (m, 2H, H-Ph), 7.08–7.09 (m, 2H, H-Ph), 7.20 (dd, 1H, H-8, *J* = 8.8 Hz, 0.5 Hz), 7.22 (t, 1H, H-2, *J* = 8 Hz), 7.43 (t, 1H, H-3, *J* = 7.1 Hz), 7.45 (d, 1H, H-1, *J* = 8 Hz), 7.58 (s, 1H, H-12), 7.61 (s, 1H, CH), 7.66 (d, 1H, H-4, *J* = 7.1 Hz). ^13^C NMR (150 MHz, CDCl_3_) δ: 43.36, 54.14, 116.91, 119.12, 120.03, 123.49, 124.06, 124.83, 125.91, 126.30, 126.56, 128.03, 128.76, 129.31, 129.46, 129.70, 131.40, 131.55, 132.44, 132.89, 140.88, 144.99, 151.52. HR MS (ESI) calcd for: C_25_H_20_N_5_S_2_ [M + H]^+^: 454.1160, found: 454.1155.

9-Fluoro-6-[(1-benzyl-1*H*-1,2,3-triazol-4-yl)-methyl]-quino[3,2-b]benzo[1,4]thiazine (**MJ6**):

Yield: 77% M.p.: 149–150 °C. ^1^H NMR (600 MHz, CDCl_3_) δ: 5.37 (s, 2H, 2CH_2_), 5.41 (s, 2H, CH_2_), 6.69–6.74 (m, 2H, H-7, H-8), 7.09–7.09 (m, 2H, 2H-Ph), 7.22–7.20 (m, 4H, H-2, 3H-Ph), 7.25–7.27 (m, 1H, H-10), 7.40–7.43 (m, 2H, H-1, H-3), 7.54 (s, 1H, H-12), 7.59 (d, 1H, H-4), 7.71 (s, 1H, CH). ^13^C NMR (150 MHz, CDCl_3_) δ: 43.47, 54.10, 113.44 (d, *J* = 24 Hz), 114.32 (d, *J* = 22.5 Hz), 117.83 (d, *J* = 7.5 Hz), 118.07, 121.90, 121.93 (d, *J* = 9 Hz), 124.71, 125.81, 126.25, 126.39, 127.89, 128.07, 128.63, 129.04, 129.75, 132.51, 134.66, 137.40 (d, *J* = 1.5 Hz), 144.43, 144.79, 151.56, 158.78 (d, *J* = 243 Hz). ^19^F NMR (564 MHz, CDCl_3_) δ: −119.53. HR MS (ESI) calcd for: C_25_H_19_FN_5_S [M + H]^+^: 440.1345, found: 440.1338.

9-Fluoro-6-((1-(4-fluorobenzyl)-1*H*-1,2,3-triazol-4-yl)methyl)-quino[3,2-b]benzo[1,4]thiazine (**MJ7**):

Yield: 78% M.p.: 146–147 °C. ^1^H NMR (600 MHz, CDCl_3_) δ: 5.45 (s, 2H, CH_2_), 5.54 (s, 2H, CH_2_), 6.73–6.78 (m, 2H, H-7, H-10), 6.89–6.92 (m, 2H, 2H-Ph), 7.08–7.11 (m, 2H, 2H-Ph), 7.23–7.27 (m, 2H, H-2, H-8), 7.43–7.46 (m, 2H, H-1, H-3), 7.59 (s, 1H, H-12), 7,67 (d, 1H, H-4), 7.76 (s, 1H, CH).^13^C NMR (150 MHz, CDCl_3_) δ: 43.73, 53.37, 113.55 (d, *J* = 22.5 Hz), 114.44 (d, *J* = 22.5 Hz), 116.04 (d, *J* = 21 Hz), 117.98 (d, *J* = 9 Hz), 118.41, 122.15 (d, *J* = 7.5 Hz), 124.70, 124.98, 125.70, 126.45, 129.79, 129.84, 130.00, 130.48 (*d*, J = 3 Hz), 132.90, 137.29 (d, *J* = 3 Hz), 143.77, 144.60, 151.44, 158.92 (d, *J* = 243 Hz), 162.77 (d, *J* = 246 Hz). ^19^F NMR (564 MHz, CDCl_3_) δ: -119.26, -112.68. HR MS (ESI) calcd for: C_25_H_18_F_2_N_5_S [M + H]^+^: 458.1251, found: 458.1254.

9-Fluoro-6-[(1-(4-chlorobenzyl)-1*H*-1,2,3-triazol-4-yl)methyl]-quino[3,2-b]benzo-[1,4]thiazine (**MJ8**):

Yield: 83% M.p.: 137–138 °C. ^1^H NMR (600 MHz, CDCl_3_) δ: 5.45 (s, 2H, CH_2_), 5.54 (s, 2H, CH_2_), 6.80–6.81 (m, 2H, H-7, H-10), 7.14–7.16 (m, 2H, 2H-Ph), 7.30–7.31 (m, 3H, H-2, 2H-Ph), 7.36–7.37 (d, 1H, H-8), 7.52–7.54 (m, 2H, H-1, H-3), 7.58 (d, 1H, H-4), 7.64 (s, 1H, H-12), 7.80 (s, 1H, CH). ^13^C NMR (150 MHz, CDCl_3_) δ: 42.98, 53.47, 113.39 (d, *J* = 25.5 Hz), 114.22 (d, *J* = 22.5 Hz), 117.43 (d, *J* = 9 Hz), 117.63, 121.64 (d, *J* = 7.5 Hz), 124.50, 125.00, 125.98, 126.39, 126.78, 129.26, 129.31, 129.49, 132.03, 133.13, 134.70, 137.53 (d, *J* = 1.5 Hz), 145.26, 151.65, 158.63 (d, *J* = 241.5 Hz). ^19^F NMR (564 MHz, CDCl_3_) δ: −119.18. HR MS (ESI) calcd for: C_25_H_18_ClFN_5_S [M + H]^+^: 474.0955, found: 474.0959.

4-((4-((9-Fluoroquino [3,2-b]benzo[1,4]thiazin-6-yl)methyl)-1H-1,2,3-triazol-1-yl)methyl)-benzonitrile (**MJ9**):

Yield: 78% M.p.: 179–180 °C. ^1^H NMR (600 MHz, CDCl_3_) δ: 5.46 (s, 2H, CH_2_), 5.55 (s, 2H, CH_2_), 6.76–6.80 (m, 2H, H-7, H-8), 7.16 (d, 2H, 2H-Ph, *J* = 8.34 Hz), 7.23–7.25 (m, 1H, H-10), 7.28 (t, 1H, H-2, *J* = 6.96 Hz), 7.46–7.49 (m, 4H, H-1, H-3, 2H-Ph), 7.64 (s, 1H, H-12), 7.80 (m, 1H, H-4), 7.90 (s, 1H, CH). ^13^C NMR (150 MHz, CDCl_3_) δ: 44.09, 53.33, 112.66, 112.66, 113.73 (d, *J* = 25.5 Hz), 114.61 (d, *J* = 21 Hz), 118.15 (d, *J* = 9 Hz), 118.91, 122.51 (d, *J* = 7.5 Hz), 125.14, 125.40, 125.56, 126.56, 128.26, 128.29, 128.34, 130.40, 132.91, 133.11, 133.51, 137.11, 139.81, 144.52, 151.26, 159.15 (d, *J* = 244.5 Hz). ^19^F NMR (564 MHz, CDCl_3_) δ: −114.49. HR MS (ESI) calcd for: C_26_H_18_FN_6_S [M + H]^+^: 465.1298, found: 465.1286.

9-Fluoro-6-((1-((phenylthio)methyl)-1*H*-1,2,3-triazol-4-yl)methyl)-quino[3,2-b]-benzo[1,4]thiazine (**MJ10**):

Yield: 75% M.p.: 121–122 °C. ^1^H NMR (600 MHz, CDCl_3_) δ: 5.34 (s, 2H, CH_2_), 5.43 (s, 2H, CH_2_), 6.69–6.72 (m, 2H, H-7, H-8), 6.96–6.98 (m, 2H, 2H-Ph), 7.02–7.03 (m, 1H, H-10), 7.08–7.10 (m, 2H, 2H-Ph), 7.17–7.19 (m, 1H, H-2), 7.20–7.23 (m, 1H, H-3), 7,41–7.45 (m, 2H, H-1, 1H-Ph), 7.53–7.54 (m, 2H, H-4, H-12), 7.57 (s, 1H, CH). ^13^C NMR (150 MHz, CDCl_3_) δ: 43.12, 54.08, 113.43 (d, *J* = 25.5 Hz), 114.23 (d, *J* = 22.5 Hz), 117.54 (d, *J* = 7.5 Hz), 117.74, 121.70, 121.76, 124.15, 124.66, 125.91, 126.36, 126.71, 128.74, 129.30, 129.65, 131.57, 132.28, 132.79, 137.34, 144.82, 145.07, 151.46, 158.69 (d, *J* = 243 Hz). ^19^F NMR (564 MHz, CDCl_3_) δ: −115.40. HR MS (ESI) calcd for: C_25_H_19_FN_5_S_2_ [M + H]^+^: 472.1066, found: 472.1067.

9-Chloro-6-[(1-benzyl-1*H*-1,2,3-triazol-4-yl)-methyl]-quino[3,2-b]benzo[1,4]thiazine (**MJ11**):

Yield: 78% M.p.: 127–128 °C. ^1^H NMR (600 MHz, CDCl_3_) δ: 5.38 (s, 2H, CH_2_), 5.52 (s, 2H, CH_2_), 7.97 (d, 1H, H-7, *J* = 2.2 Hz), 7.01 (dd, 1H, H-10, *J* = 11.2 Hz, 2.4 Hz), 7.08–7.10 (m, 2H, 2H-Ph), 7.21–7.27 (m, 4H, 3H-Ph, H-8), 7.26 (t, 1H, H-2, *J* = 7.7 Hz), 7.43–7.47(m, 2H, H-1, H-3), 7.61 (s, 1H, H-12), 7.77 (d, 1H, H-4, *J* = 8.2 Hz), 7.79 (m, 1H, CH). ^13^C NMR (150 MHz, CDCl_3_) δ: 43.91, 54.15, 118.19, 118.75, 122.27, 124.80, 125.30, 125.34, 125.47, 125.67, 126.12, 126.45, 127.86, 127.90, 127.95, 128.66, 129.05, 130.26, 133.35, 134.57, 139.56, 144.01, 151.15. HR MS (ESI) calcd for: C_25_H_19_ClN_5_S [M + H]^+^: 456.1050, found: 456.1053.

9-Chloro-6-((1-(4-fluorobenzyl)-1*H*-1,2,3-triazol-4-yl)methyl)-quino[3,2-b]benzo-[1,4]thiazine (**MJ12**):

Yield: 79% M.p.: 158–159 °C. ^1^H NMR (600 MHz, CDCl_3_) δ: 5.36 (s, 2H, CH_2_), 5.57 (s, 2H, CH_2_), 6.88–6.91 (m, 2H, 2H-Ph), 6.99 (d, 1H, H-7, *J* = 2.3 Hz), 7.09 (dd, 1H, H-10, *J* = 11 Hz, 2.3 Hz), 7.08–7.10 (m, 2H, 2H-Ph), 7.21–7.23 (m, 2H, H-2, H-8), 7.28 (t, 1H, H-1, *J* = 9.5 Hz), 7.46–7.49 (m, 3H, H-3, 2H-Ph,), 7.65 (s, 1H, H-12), 7.83 (d, 1H, H-4, *J* = 2.4 Hz), 7.85 (m, 1H, CH). ^13^C NMR (150 MHz, CDCl_3_) δ: 44.16, 53.40, 116.05 (d, *J* = 21.8 Hz), 118.33, 119.08, 122.46, 124.81, 124.95, 125.55, 126.21, 126.51, 128.00, 129.40, 129.79 (d, *J* = 9 Hz), 129.92, 130.43 (*J* = 3 Hz), 130.52, 133.74, 139.47, 142.50, 143.80, 151.02, 162.77 (*J* = 246.0 Hz). ^19^F NMR (564 MHz, CDCl_3_) δ: −112.66. HR MS (ESI) calcd for: C_25_H_18_ClFN_5_S [M + H]^+^: 474.0955, found: 474.0965.

9-Chloro-6-[(1-(4-chlorobenzyl)-1*H*-1,2,3-triazol-4-yl)methyl]-quino[3,2-b]benzo-[1,4]thiazine (**MJ13**):

Yield: 79% M.p.: 173–174 °C. ^1^H NMR (600 MHz, CDCl_3_) δ: 5.35 (s, 2H, CH_2_), 5.47 (s, 2H, CH_2_), 7.06 (d, 1H, H-7, *J* = 2.4 Hz), 7.01 (dd, 1H, H-10, *J* = 11.2 Hz, 2.4 Hz,), 7.03–7.05 (m, 2H, 2H-Ph), 7.17–7.20 (m, 2H, 2H-Ph), 7.22 (d, 1H, H-8, *J* = 11 Hz), 7.26 (t, 1H, H-1), 7.45–7.47 (m, 3H, h-3, 2H-Ph), 7.60 (s, 1H, H-12), 7.67 (d, 1H, H-4, *J* = 8.2 Hz), 7.76 (s, 1H, CH). ^13^C NMR (150 MHz, CDCl_3_) δ: 43.81, 53.38, 118.07, 118.69, 122.22, 124.79, 125.27, 125.32, 125.44, 125.68, 126.12, 126.47, 127.46, 127.90, 129.06, 130.27, 133.09, 133.28, 134.70, 139.60, 143.21, 144.27, 151.10. HR MS (ESI) calcd for: C_25_H_18_Cl_2_N_5_S [M + H]^+^: 490.0660, found: 490.0665.

4-((4-((9-Chloroquino[3,2-b]benzo[1,4]thiazin-6-yl)methyl)-1*H*-1,2,3-triazol-1-yl)methyl)-benzonitrile (**MJ14**):

Yield: 65% M.p.: 192–193 °C. ^1^H NMR (600 MHz, CDCl_3_) δ: 5.46 (s, 2H, CH_2_), 5.67 (s, 1H, CH_2_), 7.03 (d, 1H, H-7, *J* = 2.3 Hz), 7.08 (dd, 1H, H-10, J = 11.2 Hz, 2.4 Hz), 7.08–7.14 (m, 2H, 2H-Ph), 7.20 (d, 1H, H-8, *J* = 8.2 Hz), 7.34 (t, 1H, H-2, *J* = 8 Hz), 7.46–7.48 (m, 2H, 2H-Ph), 7.49–7.52 (m, 2H, H-1, H-3), 7.70 (s, 1H, H-12), 8.00 (d, 1H, H-4, *J* = 8.4 Hz), 8.02 (s, 1H, CH). ^13^C NMR (150 MHz, CDCl_3_) δ: 44.57, 53.36, 112.69, 118.11, 118.56, 119.64, 122.80, 124.20, 125.30, 125.39, 126.00, 126.39, 126.64, 128.16, 128.19, 129.94, 130.94, 132.79, 132.83, 134.41, 139.29, 139.76, 143.71, 150.86. HR MS (ESI) calcd for: C_26_H_18_ClN_6_S [M + H]^+^: 481.1002, found: 481.0997.

9-Chloro-6-((1-((phenylthio)methyl)-1*H*-1,2,3-triazol-4-yl)methyl)-quino[3,2-b]benzo[1,4]thiazine (**MJ15**):

Yield: 81% M.p.: 142–143 °C. ^1^H NMR (600 MHz, CDCl_3_) δ: 5.45 (s, 4H, 2CH_2_), 6.97 (m, 4H, H-7, H-8, 2H-Ph), 7.05–7.08 (m, 1H, H-2), 7.10–7.11 (m, 2H, 2H-Ph), 7.16 (d, 1H, H-10, *J* = 9.2 Hz), 7.27 (t, 1H, H-3, *J* = 8 Hz), 7.45–7.49 (m, 2H, H-1, 1H-Ph), 7.62 (s, 1H, H-12), 7.65 (s, 1H, CH), 7.72 (d, 1H, H-4, *J* = 8.1 Hz). ^13^C NMR (150 MHz, CDCl_3_) δ: 43.51, 54.10, 117.90, 118.34, 122.03, 124.13, 125.18, 125.80, 126.08, 126.40, 127.81, 128.75, 128.83, 129.33, 129.39, 129.44, 130.10, 131.51, 132.72, 133.03, 139.51, 144.41, 151.12. HR MS (ESI) calcd for: C_25_H_19_ClN_5_S_2_ [M + H]^+^: 488.0770, found: 488.0754.

9-Methylthio-6-[(1-benzyl-1*H*-1,2,3-triazol-4-yl)-methyl]-quino[3,2-b]benzo[1,4]thiazine (**MJ16**):

Yield: 68% M.p.: 169–170 °C. ^1^H NMR (600 MHz, CDCl_3_) δ: 2.45 (s, 3H, CH_3_), 5.48 (s, 2H, CH_2_), 5.62 (s, 2H, CH_2_), 7.00 (d, 1H, H-7, *J* = 1.6 Hz), 7.07 (dd, 1H, H-10, *J* = 10.7 Hz, 2.2 Hz), 7.17–7.19, (m, 2H, 2H-Ph), 7.28–7.35 (m, 5H, 3H-Ph, H-2, H-8), 7.52–7.55 (m, 2H, H-1, H-12), 7,70 (s, 1H, H-12), 7.87–7.89 (m, 2H, H-4, CH). ^13^C NMR (150 MHz, CDCl_3_) δ: 16.68, 43.98, 54.12, 117.65, 119.23, 121.28, 121.52, 125.10, 125.18, 125.40, 125.60, 126.38, 127.00, 127.84, 127.98, 128.61, 128.70, 129.03, 129.07, 130.11, 133.09, 134.64, 138.47, 151.24. HR MS (ESI) calcd for: C_26_H_22_N_5_S_2_ [M + H]^+^: 468.1317, found: 468.1312.

9-Methylthio-6-((1-(4-fluorobenzyl)-1*H*-1,2,3-triazol-4-yl)methyl)-quino[3,2-b]-benzo[1,4]thiazine (**MJ17**):

Yield: 82% M.p.: 154–155 °C. ^1^H NMR (600 MHz, CDCl_3_) δ: 2.44 (s, 3H, CH_3_), 5.44 (s, 2H, CH_2_), 5.51 (s, 2H, CH_2_), 6.99–7.02 (m, 4H, H-7, H-10, 2H-Ph), 7.18–7.21 (m, 2H, 2H-Ph), 7.30–7.33 (m, 2H, H-2, H-8), 7.51–7.55 (m, 2H, H-1, H-3), 7.64–7.65 (m, 2H, H-4, H-12), 7.75 (s, 1H, CH). ^13^C NMR (150 MHz, CDCl_3_) δ: 16.75, 43.37, 53.35, 116.03 (d, *J* = 21 Hz), 117.34, 118.79, 121.02, 123.51, 124.56, 124.86, 125.22, 125.77, 125.92, 126.37, 127.03, 129.79 (d, *J* = 9 Hz), 130.51 (d, *J* = 3 Hz), 132.59, 133.10, 138.67, 143.80, 144.83, 151.33, 162.76 (d, *J* = 247.5 Hz). ^19^F NMR (564 MHz, CDCl_3_) δ: −112.77. HR MS (ESI) calcd for: C_26_H_21_FN_5_S_2_ [M + H]^+^: 486.1222, found: 486.1225.

9-Methylthio-6-[(1-(4-chlorobenzyl)-1*H*-1,2,3-triazol-4-yl)methyl]-quino[3,2-b]-benzo[1,4]thiazine (**MJ18**):

Yield: 80% M.p.: 168–169 °C. ^1^H NMR (600 MHz, CDCl_3_) δ: 2.39 (s, 3H, CH_3_), 5.38 (s, 2H, CH_2_), 5.43 (s, 2H, CH_2_), 6.93 (d, 1H, H-7, *J* = 2.2 Hz), 6.98 (dd, 1H, H-10, *J* = 15.7 Hz, 2.2 Hz), 7.09 (d, 2H, 2H-Ph, *J* = 8.4 Hz), 7.23 (d, 2H, 2H-Ph, *J* = 8.4 Hz), 7.25 (t, 1H, H-2), 7.30 (d, 1H, H-8, *J* = 8.4 Hz), 7.46–7.50 (m, 2H, H-1, H-3), 7.54 (s, 1H, H-12), 7.57 (d, 1H, H-4, *J* = 8.3 Hz), 7.71 (s, 1H, CH). ^13^C NMR (150 MHz, CDCl_3_) δ: 16.78, 42.79, 53.25, 116.70, 118.14, 120.71, 124.51, 125.18, 125.94, 126.35, 126.67, 126.91, 128.26, 129.19, 129.26, 129.42, 131.93, 132.45, 133.24, 134.55, 138.83, 145.00, 145.34, 151.38. HR MS (ESI) calcd for: C_26_H_21_ClN_5_S_2_ [M + H]^+^: 502.0926, found: 502.0926.

4-((4-((9-Methylthioquino[3,2-b]benzo[1,4]thiazin-6-yl)methyl)-1H-1,2,3-triazol-1-yl)methyl)benzonitrile (**MJ19**):

Yield: 74% M.p.: 161–162 °C. ^1^H NMR (600 MHz, CDCl_3_) δ: 2.34 (s, 3H, CH_3_), 5.54 (s, 2H, CH_2_), 5.66 (s, 2H, CH_2_), 6.89 (m, 1H, H-7), 6.93 (d, 1H, H-8), 7.16 (d, 2H, 2H-Ph, *J* = 8.1 Hz), 7.19–7.23 (m, 3H, H-2, H-10), 7.41–7.45 (m, 2H, H-1, H-3), 7.50 (d, 2H, 2H-Ph, *J* = 7.5 Hz), 7.54–7.55 (m, 2H, H-4, H-12), 7.66 (s, 1H, CH). ^13^C NMR (150 MHz, CDCl_3_) δ: 16.79. 42.87, 53.30, 112.60, 116.92, 118.18, 120.78, 120.81, 124.61, 124.67, 125.24, 125.98, 126.40, 126.63, 126.98, 128.23, 129.46, 129.50, 132.02, 132.09, 132.80, 138.79, 139.88, 145.73, 151.43. HR MS (ESI) calcd for: C_27_H_21_N_6_S_2_ [M + H]^+^: 493.1296, found: 493.1298.

9-Methylthio-6-((1-((phenylthio)methyl)-1*H*-1,2,3-triazol-4-yl)methyl)-quino[3,2-b]benzo[1,4]thiazine (**MJ20**):

Yield: 54% M.p.: 173–174 °C. ^1^H NMR (600 MHz, CDCl_3_) δ: 2.35 (s, 3H, CH_3_), 5.36 (s, 2H, CH_2_), 5.44 (s, 2H, CH_2_), 6.91 (d, 1H, H-7, *J* = 2.2 Hz), 6.93 (dd, 1H, H-10, *J* = 10.6 Hz, 2.0 Hz), 6.96–6.99 (m, 2H, 1H-Ph, H-2), 7.04 (t, 1H, H-2, *J* = 7.3 Hz), 7.10 (d, 2H, 2H-Ph, *J* = 7.2 Hz), 7.16 (d, 1H, H-8, *J* = 17.6Hz), 7.23 (t, 1H, H-3, *J* = 7.9 Hz), 7.43 (t, 1H, 1-Ph, *J* = 7.0 Hz), 7.45 (d, 1H, H-1, *J* = Hz), 7.50–7.51 (m, 2H, H-4, H-12), 7.57 (s, 1H, CH). ^13^C NMR (150 MHz, CDCl_3_) δ: 16.86, 42.83, 54.13, 116.98, 118.23, 120.68, 123.99, 124.58, 125.32, 125.98, 126.28, 126.81, 127.08, 128.77, 129.46, 131.54, 132.05, 132.17, 132.50, 132.91, 138.81, 144.88, 145.23, 151.36. HR MS (ESI) calcd for: C_26_H_22_N_5_S_3_ [M + H]^+^: 500.1073, found: 500.1040.

### 3.3. Microscopic Observations

The BEAS-2B, NHDF, HCT116, MCF7, SH-SY5Y, A549 cell lines studied were cultured in DMEM medium supplemented with 10% fetal bovine serum (FBS) and 1% penicillin/streptomycin solution. Cells were seeded at 1 × 10^4^ per well in 96-well plates and incubated for 24 h under standard culture conditions: temperature 37 °C, 5% CO_2_, 95% humidity. After 24 h, compounds from the **MJ1**–**MJ20** group were added to individual wells at different concentrations (6.25 µM, 12.5 µM, 25 µM, 50 µM, 100 µM). The control consisted of cells grown only in medium without the addition of chemical compounds. After 24 h of incubation, cells were observed under a microscope (JuLI™ FL Live fluorescence microscope from NanoEntek, Martinsried, Germany), documenting morphological changes. Observations included visual assessments of confluence, cell shape, arrangement, and interaction with the substrate. The Supplementary Materials include a comprehensive collection of microscopic images showing the morphology of the tested cell lines (BEAS-2B, NHDF, HCT116, MCF7, SH-SY5Y, A549) after 24 h of incubation with compounds **MJ1**–**MJ20**. These images illustrate the effects of the compounds on cell shape, density, and adhesion. The images are provided as a separate PDF file for further reference. For apoptosis observations, the samples of BEAS-2B and HCT116 cells were additionally fixed with 4% paraformaldehyde (PFA) after 24 h of incubation with, or without selected compounds (Section 2.6) and DAPI stained for apoptotic bodies visualization (Fluoromount-G™ medium with DAPI dye; Thermo Fisher Scientific, Waltham, MA, USA). The samples were visualized using fluorescent microscopy AXIO IMAGER M1 by ZEISS, DAPI channel and 20x magnification. The percent of counted cells with apoptotic nucleus was defined in comparison to all visualized events.

### 3.4. Alamar Blue Test

In cytotoxicity studies, the Alamar Blue test was used, which is based on the ability of living cells to reduce the non-fluorescent dye resazurin to fluorescent resorufin, which reflects the metabolic activity of cells. BEAS-2B, NHDF, HCT116, MCF7, A549, SH-SY5Y and U2OS cell lines were cultured under standard conditions, in DMEM medium supplemented with 10% FBS and 1% penicillin/streptomycin, at 37 °C, 5% CO_2_ and 95% humidity. Cells were seeded in 96-well plates at 1 × 10^4^ cells per well and incubated for 24 h. Then, the tested compounds (**MJ1**–**MJ20**) were added at different concentrations (from 6.25 µM to 100 µM). The control contained cells untreated with the tested compounds. After 24 h of incubation, 10 µL of Alamar Blue solution was added to each well and incubated for another 2–4 h under the same conditions. The signal was measured spectrophotometrically at wavelengths of 570 nm (for resorufin) and 600 nm (for resazurin) or optionally fluorimetrically, using an excitation maximum of 570 nm and an emission maximum of 585 nm. The results were presented as percentage of cell survival relative to the control, calculating the ratio of absorbance or fluorescence of the sample to the control value. The Alamar Blue assay, due to its sensitivity and non-toxicity, allows for an accurate assessment of metabolic activity and cell survival in response to the tested quinobenzothiazines.

### 3.5. RT-qPCR

Gene expression studies used quantitative RT-qPCR to assess the effect of the tested quinobenzothiazines (**MJ1**–**MJ20**) on the expression of apoptosis-related genes (BCL2, AIFM2, MDM2) and pro-inflammatory cytokines (IL6, IL8) (Table 4).

BEAS-2B and HCT116 cells were seeded in 6-well plates at 2 × 10^5^ cells per well and cultured in RPMI-1640 (BEAS-2B) or DMEM (HCT116) medium supplemented with 10% FBS and 1% penicillin/streptomycin at 37 °C, 5% CO_2_ and 95% humidity. After 24 h of incubation, the tested compounds were added at a concentration of 100 µM. After 24 h, the medium was removed from the cells, washed with PBS solution, 0.5 mL of trypsin was added per well and after 5 min it was neutralized with medium. The obtained cell suspension was centrifuged for 3 min at 2000 rpm, the supernatant was removed, and the obtained pellet was suspended in 400 µL of phenozol. Samples were frozen at −20 °C and stored until further experiment and RNA isolation.

Total RNA was isolated from cells using the A&A Biotechnology Total RNA Mini kit according to the manufacturer’s protocol. RNA quality and quantity were assessed using a NanoDrop 2000 spectrophotometer at 260/280 nm. Reverse transcription was performed using the protocol (EURx NG dART RT Kit) to obtain cDNA. After incubation, samples were placed in a freezer (−20 °C) and stored until further analysis.

The experiment was performed according to the protocol (Brilliant III Ultra Fast SYBR^®^ Green QPCR Master Mix, Quick Reference Guide for the Bio-Rad CFX96 Real-Time PCR Detection System). RT-qPCR reaction was performed using specific primers for target genes (BCL2, AIFM2, MDM2, IL6, IL8). Gene expression was normalized to the expression of the reference gene RPL41. Reaction profiles and results were performed using Bio-Rad CFX Maestro 1.1 software from Bio-Rad. Analysis and calculations of the results obtained during RT-qPCR were performed using Microsoft Excel.

The results were analyzed using the R = 2^−ΔΔCt^ method, which allows for determining the relative level of gene expression (R) compared to the control [63]. Each experiment was performed in three technical and biological replicates. RT-qPCR allowed for precise assessment of changes in the expression of genes related to the processes of apoptosis and inflammatory response under the influence of quinobenzothiazines, which allowed for identifying their potential mechanisms of action at the molecular level.

### 3.6. DAPI Staining for Nuclear Morphology Assessment

BEAS-2B and HCT116 cells were seeded into culture plates at 5 × 10^3^ cells per well using 0.5 mL of RPMI-1640 medium (for BEAS-2B) or DMEM (for HCT116). The plates were incubated for 24 h at 37 °C in an atmosphere containing 5% CO_2_. After incubation, the medium was removed and compounds **MJ2**, **MJ8**, **MJ15**, and **MJ19** were added in the appropriate medium at a concentration of 100 μM. The plates were incubated for 24 h at 37 °C in an atmosphere containing 5% CO_2_. After incubation, the medium was removed, and 500 µL of 4% paraformaldehyde (PFA) was added to each well to fix the cells. Incubation continued for 10 min at room temperature. After removal of the PFA, the wells were rinsed with 500 µL of PBS (5 min of incubation) and then with distilled water. The cells were allowed to dry at room temperature. After drying, the cells were stained with DAPI. Observations were made using a fluorescence microscope AXIO IMAGER M1 by ZEISS at 20x magnification.

## 4. Summary

Studies conducted on cell lines using quinobenzothiazines (**MJ1**–**MJ20**) provided interesting results that shed light on their potential therapeutic applications in various fields of medicine. The results of analyses conducted using the PASS server indicated a wide potential of the compounds studied in the modulation of biological processes, including regulation of anaphylotoxin receptors and inflammatory mechanisms. Especially compounds **MJ19** and **MJ20**, which showed high Pa values for activities related to inflammatory processes and modulation of the immune system, confirmed their potential also in laboratory studies. This indicates a possible direction of research, combining the ability of quinobenzothiazines to modulate the cellular microenvironment with their potential use in the therapy of inflammatory and autoimmune diseases.

Microscopic observations showed that the effect of quinobenzothiazines on cell morphology depends on their concentration. At low concentrations (6.25–25 µM), cells retained their typical morphology, and changes were minimal. High concentrations (50–100 µM) induced significant changes, such as cell detachment from the substrate and their adoption of a spherical shape, which can indicate the initial stages of cell death, such as apoptosis or necrosis. Cancer cell lines showed greater susceptibility to morphological changes in response to quinobenzothiazines compared to physiological NHDF cells, which may suggest their selectivity in action on different cell types.

The results of the Alamar Blue test indicated limited cytotoxicity of the tested compounds against the tested cell lines. For BEAS-2B cells, the IC_50_ values were determined, which were high, suggesting the safety of these compounds against healthy cells. On the other hand, for cancer lines (HCT116, A549, SH-SY5Y, MCF7, U2OS), the IC_50_ values were unmeasurable, which may indicate mechanisms of resistance to cytotoxicity of these compounds. The activation of gene expression, such as BCL2, and genes related to inflammation processes, such as IL6 and IL8, in healthy cells suggests that these compounds can induce cellular resistance. Moreover, increased AIFM2 expression indicates the potential effect of quinobenzothiazines on alternative cell death pathways, such as ferroptosis, which opens new directions for research on structural modifications of these compounds. These results emphasize the potential of quinobenzothiazines as research tools in the analysis of the mechanisms of chemoresistance of cancer cells.

Gene expression analysis performed on the BEAS-2B line showed a significant increase in the expression of genes related to cell survival and resistance to cell death. Increased expression of BCL2, MDM2, and AIFM2 indicates the activation of protective mechanisms against apoptosis and ferroptosis, which may support cell survival under stress conditions. Induction of pro-inflammatory genes, such as IL6 and IL8, especially in response to **MJ19** and **MJ20**, suggests that the compounds studied may modulate inflammatory pathways and affect the cellular microenvironment. These properties may be beneficial in the context of immunotherapy, where controlled activation of the immune system is desirable. At the same time, increased expression of IL6 and IL8 may in some cases support processes associated with tumor progression, such as angiogenesis or cell migration.

The compound **MJ19** was found to significantly increase the expression of the BCL2 and AIFM2 genes, as well as the pro-inflammatory cytokines IL6 and IL8, in the BEAS-2B cell line. This indicates its potential anti-apoptotic and protective effects on cells, as well as its ability to modulate the inflammatory response. The high IC_50_ value for the BEAS-2B cell line suggests that **MJ19** is safe for healthy cells and does not directly cause cell death, which could make it a candidate for supportive therapy or for use in conditions requiring cell protection and control of inflammatory processes.

Moreover, compound **MJ2** significantly increased the expression of the AIFM2 gene in BEAS-2B cells, indicating a potential role in activating protective mechanisms against ferroptosis. This suggests that **MJ2** may contribute to the modulation of cell survival pathways, which could be beneficial in the context of inflammatory or degenerative diseases.

Based on the conducted analyses, it can be concluded that the tested compound derivatives demonstrate the ability to selectively modulate the expression of genes involved in apoptosis, oxidative stress, and the inflammatory response in a cell-type-dependent manner. Compounds **MJ2**, **MJ8**, **MJ15**, and **MJ19** had a stronger effect on the expression of the BCL2, MDM2, AIFM2, IL6, and IL8 genes in the healthy BEAS-2B cell line than in the cancerous HCT116 cell line, which may indicate their potential protective effect on normal cells with limited effect on cancer cells. Of particular importance is the ability of **MJ19** to strongly induce pro-inflammatory cytokines and antiapoptotic genes, which may make this compound an interesting candidate for further research into adjunctive therapies.

## 5. Conclusions

In summary, the tested quinobenzothiazines exhibit complex biological activity, including the ability to modulate cell survival pathways and immune response, making them promising candidates for further studies. The results suggest their potential in supportive therapy and as research tools in the analysis of mechanisms of chemoresistance and ferroptosis. The **MJ19** compound stands out in particular, as it induced high expression of pro-inflammatory genes and protective mechanisms against cell death, which may indicate its potential use in the therapy of inflammatory, autoimmune diseases and in the protection of healthy cells from environmental stress. Quinobenzothiazines are a valuable starting point for further studies on their therapeutic application, both in oncology and in diseases of inflammatory or degenerative origin.

## Figures and Tables

**Table 1:** Results of the prediction of cytotoxicity of compounds **MJ1**–**MJ20** (Pa—probability of activity, Pi—probability of inactivity).

	Pa	Pi	Activity		Pa	Pi	Activity
**MJ1**	0.746	0.018	Anaphylatoxin receptor antagonist	**MJ11**	0.702	0.023	Anaphylatoxin receptor antagonist
	0.681	0.041	Glycosylphosphatidylinositol phospholipase D inhibitor		0.694	0.037	Glycosylphosphatidylinositol phospholipase D inhibitor
	0.574	0.011	Atherosclerosis treatment		0.531	0.015	Atherosclerosis treatment
	0.503	0.027	Autoimmune disorders treatment		0.484	0.031	Autoimmune disorders treatment
	0.493	0.004	Leukotriene synthesis inhibitor		0.460	0.004	Leukotriene synthesis inhibitor
**MJ2**	0.665	0.029	Anaphylatoxin receptor antagonist	**MJ12**	0.619	0.036	Anaphylatoxin receptor antagonist
	0.579	0.010	Atherosclerosis treatment		0.542	0.014	Atherosclerosis treatment
	0.522	0.023	Autoimmune disorders treatment		0.503	0.027	Autoimmune disorders treatment
	0.478	0.004	Leukotriene synthesis inhibitor		0.474	0.110	Glycosylphosphatidylinositol phospholipase D inhibitor
	0.453	0.004	Allergic rhinitis treatment		0.450	0.004	Leukotriene synthesis inhibitor
**MJ3**	0.757	0.016	Anaphylatoxin receptor antagonist	**MJ13**	0.702	0.023	Anaphylatoxin receptor antagonist
	0.754	0.022	Glycosylphosphatidylinositol phospholipase D inhibitor		0.694	0.037	Glycosylphosphatidylinositol phospholipase D inhibitor
	0.546	0.013	Atherosclerosis treatment		0.531	0.015	Atherosclerosis treatment
	0.471	0.034	Autoimmune disorders treatment		0.484	0.031	Autoimmune disorders treatment
	0.466	0.004	Leukotriene synthesis inhibitor		0.460	0.004	Leukotriene synthesis inhibitor
**MJ4**	0.665	0.029	Anaphylatoxin receptor antagonist	**MJ14**	0.619	0.036	Anaphylatoxin receptor antagonist
	0.542	0.014	Atherosclerosis treatment		0.495	0.018	Atherosclerosis treatment
	0.550	0.044	Neurotransmitter uptake inhibitor		0.493	0.029	Autoimmune disorders treatment
	0.511	0.025	Autoimmune disorders treatment		0.474	0.110	Glycosylphosphatidylinositol phospholipase D inhibitor
	0.480	0.009	CYP2C19 inhibitor		0.439	0.013	CYP2C19 inhibitor
**MJ5**	0.740	0.019	Anaphylatoxin receptor antagonist	**MJ15**	0.696	0.024	Anaphylatoxin receptor antagonist
	0.671	0.043	Glycosylphosphatidylinositol phospholipase D inhibitor		0.684	0.040	Glycosylphosphatidylinositol phospholipase D inhibitor
	0.650	0.006	Atherosclerosis treatment		0.615	0.008	Atherosclerosis treatment
	0.436	0.037	Interleukin 2 agonist		0.439	0.119	Nicotinic alpha4beta4 receptor agonist
	0.414	0.140	Nicotinic alpha4beta4 receptor agonist		0.419	0.044	Interleukin 2 agonist
**MJ6**	0.740	0.019	Anaphylatoxin receptor antagonist	**MJ16**	0.596	0.041	Anaphylatoxin receptor antagonist
	0.671	0.043	Glycosylphosphatidylinositol phospholipase D inhibitor		0.567	0.011	Atherosclerosis treatment
	0.650	0.006	Atherosclerosis treatment		0.524	0.023	Autoimmune disorders treatment
	0.436	0.037	Interleukin 2 agonist		0.430	0.005	Leukotriene synthesis inhibitor
	0.414	0.140	Nicotinic alpha4beta4 receptor agonist		0.424	0.002	Transglutaminase 2 inhibitor
	0.740	0.019	Anaphylatoxin receptor antagonist		0.596	0.041	Anaphylatoxin receptor antagonist
**MJ7**	0.579	0.044	Anaphylatoxin receptor antagonist	**MJ17**	0.572	0.011	Atherosclerosis treatment
	0.542	0.014	Atherosclerosis treatment		0.536	0.021	Autoimmune disorders treatment
	0.471	0.004	Leukotriene synthesis inhibitor		0.504	0.063	Anaphylatoxin receptor antagonist
	0.470	0.035	Autoimmune disorders treatment		0.427	0.083	Antiinflammatory
	0.446	0.004	Allergic rhinitis treatment		0.423	0.005	Leukotriene synthesis inhibitor
**MJ8**	0.619	0.036	Anaphylatoxin receptor antagonist	**MJ18**	0.632	0.034	Anaphylatoxin receptor antagonist
	0.512	0.017	Atherosclerosis treatment		0.541	0.014	Atherosclerosis treatment
	0.474	0.110	Glycosylphosphatidylinositol phospholipase D inhibitor		0.509	0.096	Glycosylphosphatidylinositol phospholipase D inhibitor
	0.450	0.004	Leukotriene synthesis inhibitor		0.491	0.029	Autoimmune disorders treatment
	0.444	0.042	Autoimmune disorders treatment		0.413	0.005	Leukotriene synthesis inhibitor
**MJ9**	0.508	0.017	Atherosclerosis treatment	**MJ19**	0.538	0.014	Atherosclerosis treatment
	0.486	0.068	Anaphylatoxin receptor antagonist		0.528	0.022	Autoimmune disorders treatment
	0.479	0.032	Autoimmune disorders treatment		0.504	0.063	Anaphylatoxin receptor antagonist
	0.439	0.004	Leukotriene synthesis inhibitor		0.418	0.102	Neurotransmitter uptake inhibitor
	0.430	0.095	Neurotransmitter uptake inhibitor		0.400	0.005	Leukotriene synthesis inhibitor
**MJ10**	0.621	0.008	Atherosclerosis treatment	**MJ20**	0.628	0.007	Atherosclerosis treatment
	0.577	0.044	Anaphylatoxin receptor antagonist		0.596	0.041	Anaphylatoxin receptor antagonist
	0.433	0.039	Interleukin 2 agonist		0.404	0.144	Glycosylphosphatidylinositol phospholipase D inhibitor
	0.371	0.047	Anxiolytic		0.391	0.059	Interleukin 2 agonist
	0.365	0.044	Cognition disorders treatment		0.377	0.038	MAP3K5 inhibitor

**Table 2:** IC_50_ values for BEAS-2B line [μM].

**MJ1**	**MJ2**	**MJ3**	**MJ4**	**MJ5**	**MJ6**	**MJ7**	**MJ8**	**MJ9**	**MJ10**
73.838	76.163	75.436	64.526	72.958	72.869	73.617	71.858	72.961	72.267
**MJ11**	**MJ12**	**MJ13**	**MJ14**	**MJ15**	**MJ16**	**MJ17**	**MJ18**	**MJ19**	**MJ20**
88.062	75.635	85.203	78.428	87.21	84.727	72.595	74.130	72.719	83.657

**Table 4:** Primer sequences used in the rt-qPCR reaction.

Gene	Primer Forward	Primer Reverse
**BCL2**	**CTTCAGGGACGGGGTGAAC**	**GGATCCAGGTGTGCAGGTG**
**MDM2**	**GCCCTTCGTGAGAATTGGCT**	**CCTCAACACATGACTCTCTGG**
**AIFM2**	**CTGCCCTTCTCTCATCTTATCCT**	**CTGCCTCACCATGTCCTCATAG**
**IL6**	**GTTCTGCCAAACCAGCCTTG**	**AGATCACCTAGTCCACCCCC**
**IL8**	**GGTGCAGTTTTGCCAAGGAG**	**ACCAAGGCACAGTGGAACAA**
**RPL41**	**TCCTGCGTTGGGATTCCGTG**	**ACGGTGCAACAAGCTAGCGG**

## Data Availability

Data are contained within the article and Supplementary Materials.

## Supplementary Materials

The following supporting information can be downloaded at https://www.mdpi.com/article/10.3390/ijms26146920/s1, File S1: ^1^H NMR and ^13^C NMR spectra and HR MS of compounds **MJ1**–**MJ20**; File S2: images of BEAS-2B, NHDF, HCT116, MCF7, SH-SY5Y, and A549 cells after 24 h incubation with **MJ1**–**MJ20** compounds at concentrations of 6.25–100 µM, images of BEAS-2B and HCT116 cell line nuclei after 24 h of incubation with **MJ2**, **MJ8**, **MJ15**, **MJ19** compounds at concentration of 100 µM after DAPI staining.

## Abbreviations

The following abbreviations are used in this manuscript:

AIFM2	Apoptosis-Inducing Factor Mitochondria-Associated 2
A549	Human lung adenocarcinoma cell line
BEAS-2B	Human bronchial epithelial cell line
BCL2	B-cell CLL/lymphoma 2
COSY	COrrelation SpectroscopY
CuAAC	Copper(I)-catalyzed Azide-Alkyne Cycloaddition
DMEM	Dulbecco’s Modified Eagle Medium
FBS	Fetal Bovine Serum
HCT116	Human colon cancer cell line
IL6	Interleukin 6
IL8	Interleukin 8
MCF7	Human breast cancer cell line
MDM2	Mouse Double Minute 2 homolog
NHDF	Normal Human Dermal Fibroblasts
NOESY	Nuclear Overhauser effect Spectroscopy
PASS	Prediction of Activity Spectra for Substances
PDE3	Phosphodiesterase 3
RPL41	Ribosomal Protein L41
RT-qPCR	quantitative Real-Time Polymerase Chain Reaction
SH-SY5Y	Human neuroblastoma cell line
U2OS	Human osteosarcoma cell line
