# Supplementary material for: Design, Synthesis, and Testing of 1,2,3-Triazolo-Quinobenzothiazine Hybrids for Cytotoxic and Immunomodulatory Activity
Source: Int J Mol Sci. 2025 Jul 18;26(14):6920. doi: 10.3390/ijms26146920 (PMC12295927; doi:10.3390/ijms26146920)
Supplement: Supplementary file 1 [file ijms-26-06920-s001.zip › Supplementary Materials 1.pdf]

Design, synthesis, and cytotoxic properties of 1,2,3-triazolo-azaphenothiazines hybrids.

Klaudia Giercuszkiewicz-Haśnik [1,2], Magdalena Skonieczna [1,2], Beata Morak-Młodawska [3], Małgorzata Jeleń [3*]

[1] Department of Systems Biology and Engineering, The Silesian University of Technology, Akademicka Street 16, 44–100 Gliwice, Poland; magdalena.skonieczna@polsl.pl (M.S.)
[2] Centre of Biotechnology, Silesian University of Technology, Krzywoustego Street 8, 44-100 Gliwice, Poland
[3] Department of Organic Chemistry, Faculty of Pharmaceutical Sciences in Sosnowiec, Medical University of Silesia in Katowice, Jagiellońska Street 4, 41-200 Sosnowiec, Poland; bmlodawska@sum.edu.pl (B.M.-M.)

Content:

1. Synthesis of substrates for the preparation of the title tested 1,2,3-triazole-quinobenzothiazine hybrids. 2
2. ^{1}H NMR and ^{13}C NMR spectra and HR MS of compounds **MJ1-MJ20**. 3

1. Synthesis of substrates for the preparation of the title tested 1,2,3-triazole-quinobenzothiazine hybrids.

1.1. Synthesis of 6*H*-quino[3,2-b]benzo[1,4]thiazines **QBT1**-**QBT4**.

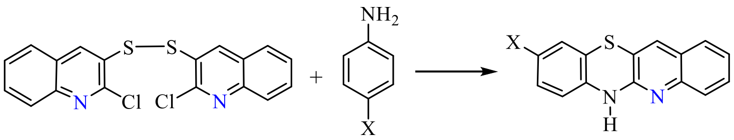

DS **QBT 1 - 4**

X = H, F, Cl, SCH_3

A solution of 2,2′-dichloro-3,3′-diquinolinyl disulfide (**DS**) (0.40g, 1 mmol) and aniline, *p*-fluoroaniline, *p*-chloroaniline or *p*-methylthioaniline (4 mmol) in MEDG (10 mL) was refluxed for 3 h. After cooling the solution was poured into water (40 mL) and alkalized with 5% aqueous sodium hydroxide to pH = 10. The resulting solid was filtered off, washed with water and purified by column chromatography (silica gel, $CHCl_3$) to give appropriately:

6*H*-quino[3,2-b]benzo[1,4]thiazine **QBT1** (0.26 g, 52%), mp 169-170 °C,
6*H*-9-fluoroquino[3,2-b]benzo[1,4]thiazine **QBT2** (0.30 g, 56%), mp 158-159 °C,
6*H*-9-chloroquino[3,2-b]benzo[1,4]thiazine **QBT3** (0.38 g, 67%), mp 224-225 °C,
6*H*-9-methtlthioquino[3,2-b]benzo[1,4]thiazine **QBT4** (0.36 g, 60%), mp 204-205 °C [46].

1.2. Synthesis of propargyl- quino[3,2-b]benzo[1,4]thiazines **PrQBT1**-**PrQBT4**.

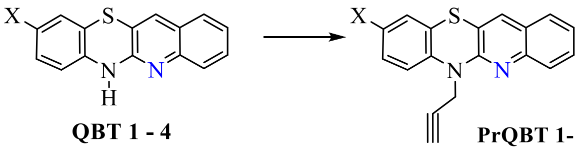

QBT 1 - 4 **PrQBT 1- 4**

X = H, F, Cl, SCH_3

To a solution of appropriately 9-substituted 6*H*-quino[3,2-b]benzo[1,4]thiazine (**QBT1**-**QBT4**) (1 mmol) in dry DMF (5 mL) potassium tert-butoxide (0.16 g, 1.44 mmol) was added. The mixture was stirred at room temperature for 1 h. Then 80% solution of propargyl bromide in toluene (0.3 mL, 2.7 mmol) was added dropwise. The solution was stirred at room temperature for 24 h and poured into water (20 mL), extracted with methylene chloride (20 mL), dried with Na_2SO_4 and evaporated to the brown oil. The residue was purified by column chromatography (silica gel, CH_2Cl_2) to give appropriately:

6-propargyl-quino[3,2-b]benzo[1,4]thiazine (**PrQBT1**) (0.21 g, 73%), mp 141-142 °C [47],

9-fluoro-6-propargyl-quino[3,2-b]benzo[1,4]thiazine (**PrQBT2**) (0.24 g, 78%), mp 124-125 °C [48],

9-chloro-6-propargyl-quino[3,2-b]benzo[1,4]thiazine (**PrQBT3**) (0.24 g, 75%), mp 169-170 °C [47],

9-methylthio-6-propargyl-quino[3,2-b]benzo[1,4]thiazine (**PrQBT4**) (0.25 g, 75%), mp 154-155 °C [47].

3. ^{1}H NMR and ^{13}C NMR spectra and HR MS of compounds **MJ1-MJ20**.

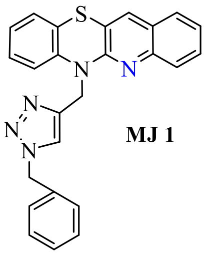

MJ 1

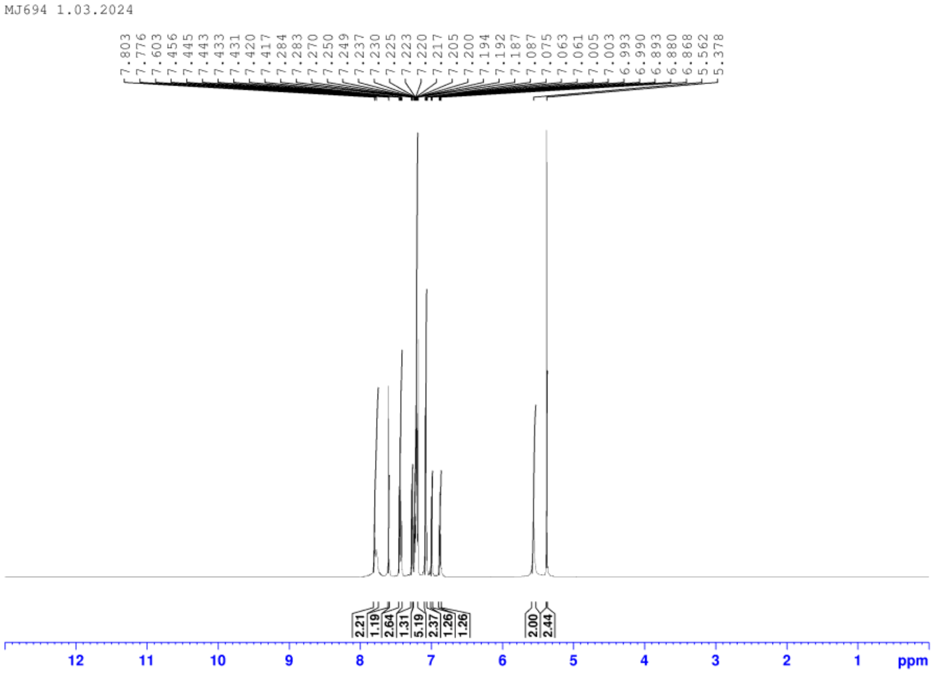

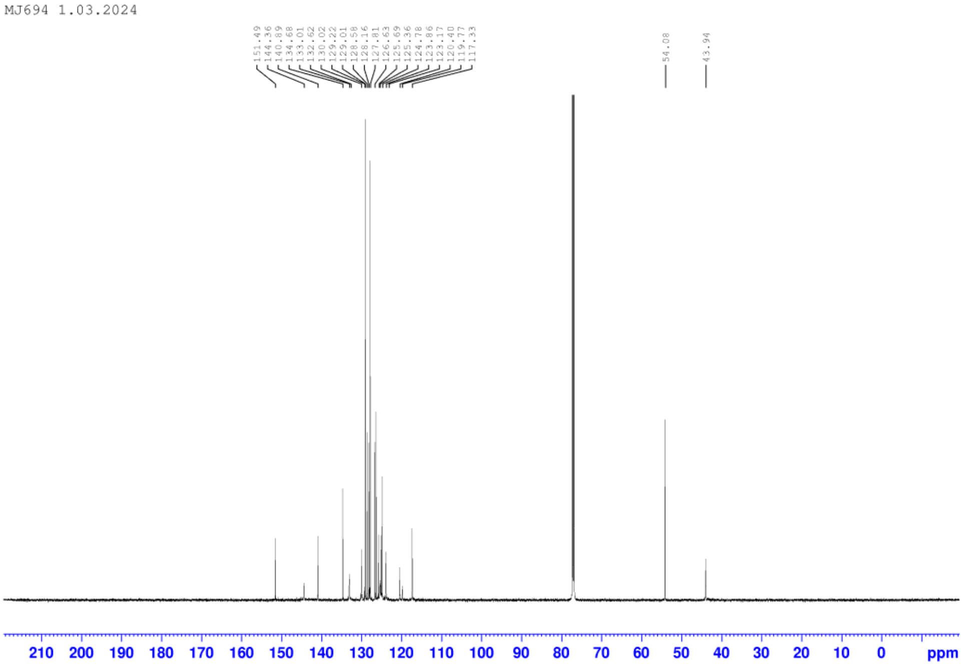

Acquisition Parameter

Source Type	ESI	Ion Polarity	Positive	Set Nebulizer	0.3 Bar
Focus	Active	Set Capillary	4000 V	Set Dry Heater	240 °C
Scan Begin	100 m/z	Set End Plate Offset	-500 V	Set Dry Gas	4.0 l/min
Scan End	1000 m/z	Set Charging Voltage	2000 V	Set Divert Valve	Source
		Set Corona	0 nA	Set APCI Heater	0 °C

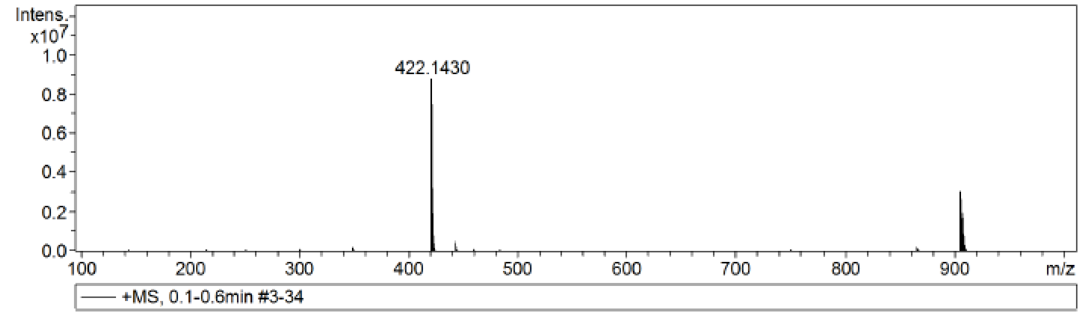

#	m/z	Res.	S/N	I	I %	FWHM
1	422.1430	46499	29186.8	8787963	100.0	0.0091

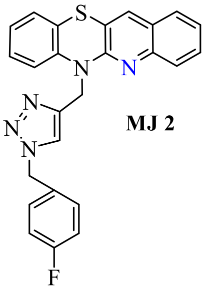

MJ 2

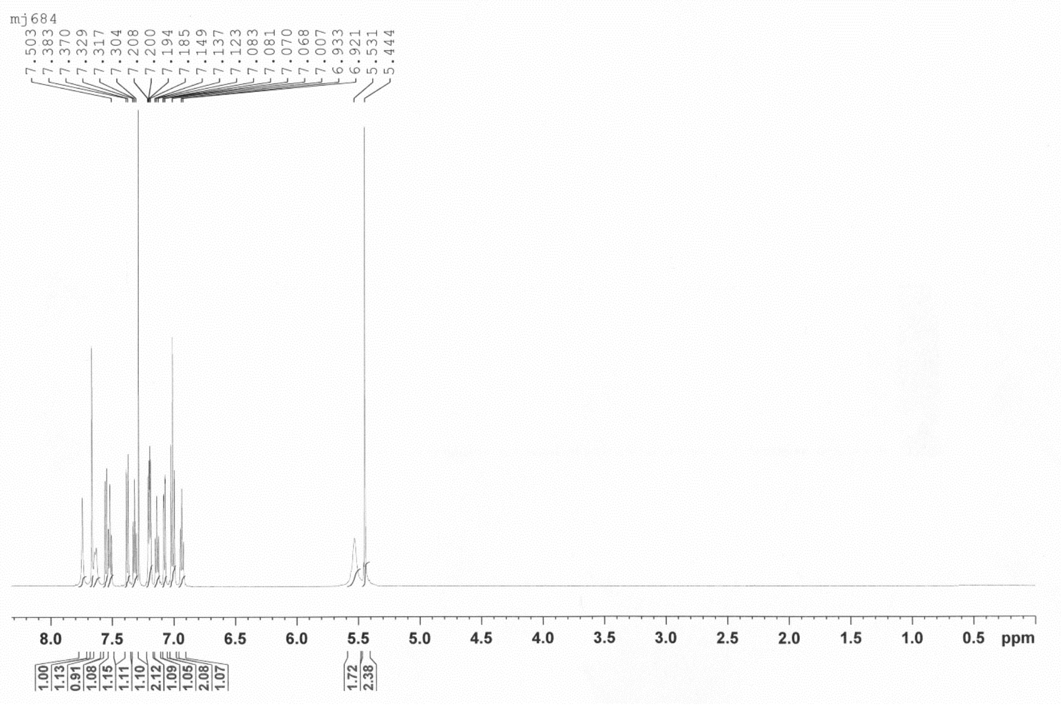

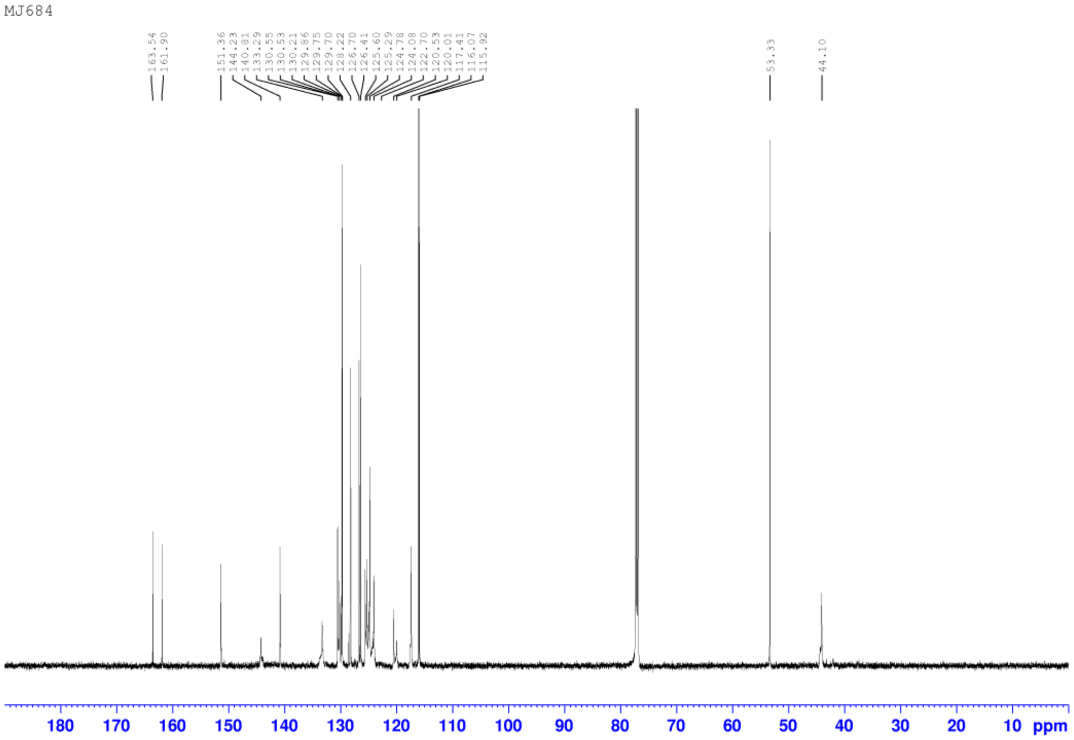

Acquisition Parameter

Source Type	ESI	Ion Polarity	Positive	Set Nebulizer	0.3 Bar
Focus	Active	Set Capillary	4000 V	Set Dry Heater	240 °C
Scan Begin	100 m/z	Set End Plate Offset	-500 V	Set Dry Gas	4.0 l/min
Scan End	1000 m/z	Set Charging Voltage	2000 V	Set Divert Valve	Source
		Set Corona	0 nA	Set APCI Heater	0 °C

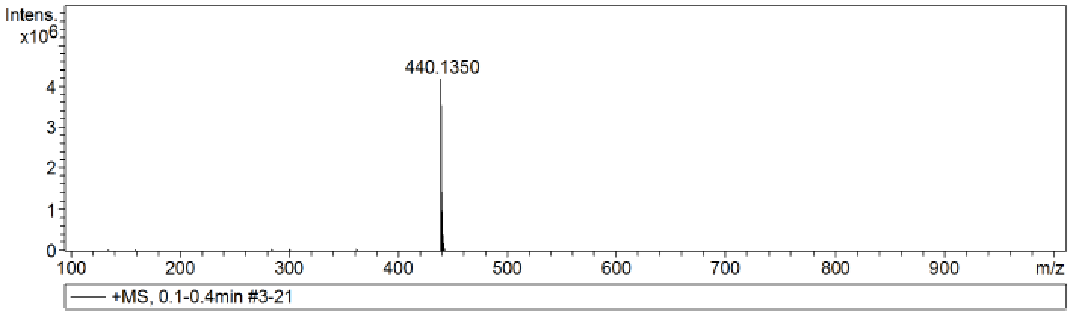

#	m/z	Res.	S/N	I	I %	FWHM
1	440.1350	46682	33045.3	4205728	100.0	0.0094

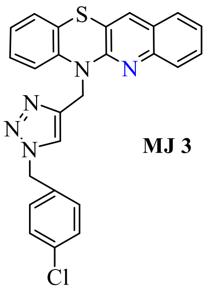
S
N
N
N
N
N
Cl

MJ 3

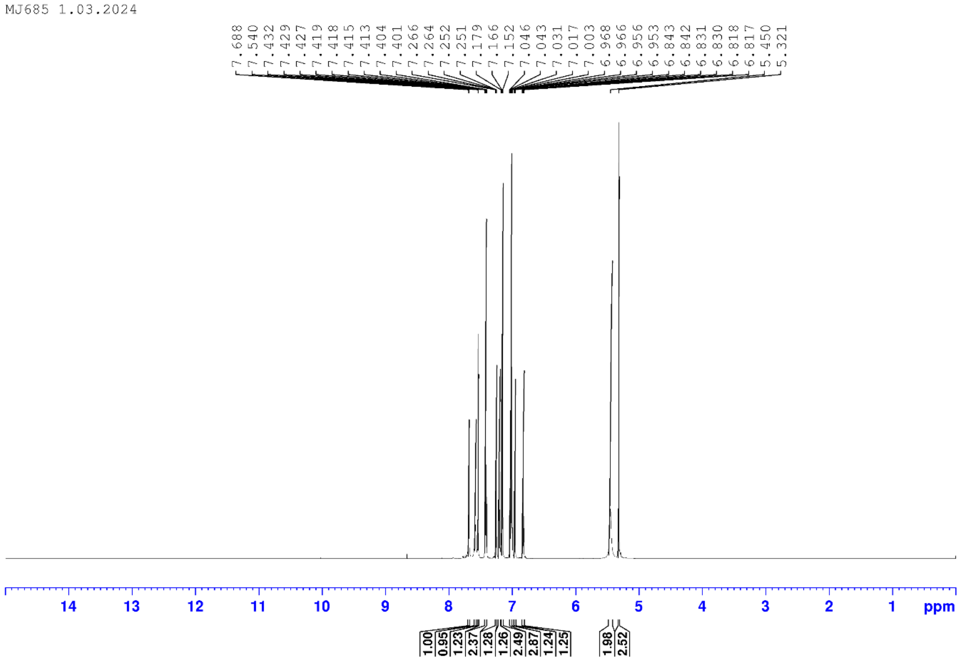
MJ685 1.03.2024
7.688
7.540
7.432
7.429
7.427
7.419
7.418
7.415
7.413
7.404
7.401
7.266
7.264
7.252
7.251
7.179
7.166
7.152
7.046
7.043
7.031
7.017
7.003
6.968
6.966
6.956
6.953
6.843
6.842
6.831
6.830
6.818
6.817
5.450
5.321
14
13
12
11
10
9
8
7
6
5
4
3
2
1
ppm
1.00
0.95
1.23
2.37
1.28
1.26
2.49
2.87
1.24
1.25
1.98
2.52

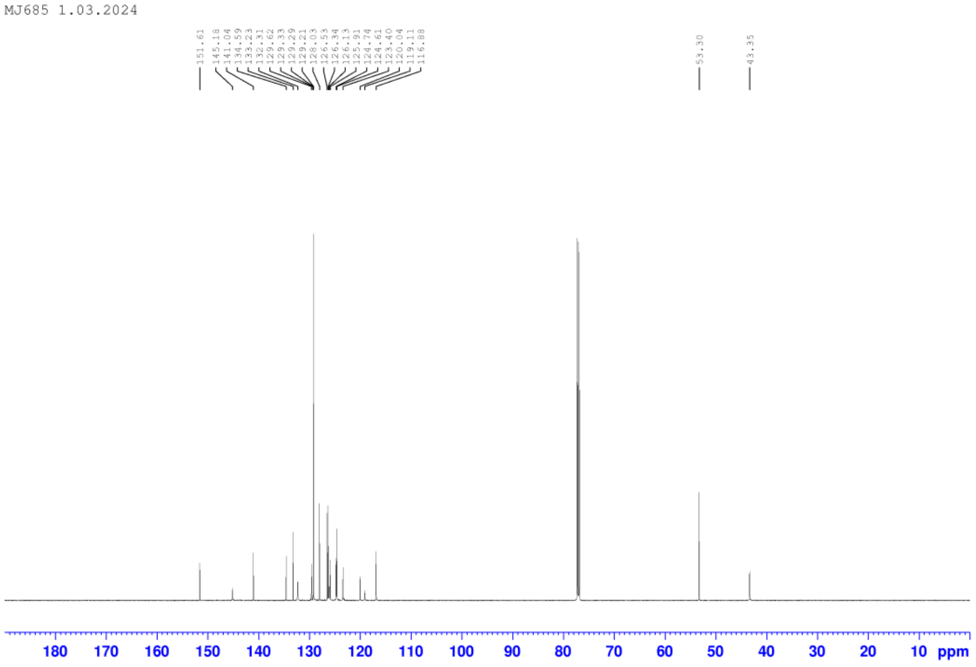
MJ685 1.03.2024
151.61
145.18
141.04
134.59
133.23
132.31
129.62
129.33
129.29
129.21
128.03
126.53
126.34
126.13
125.91
124.74
124.61
123.40
120.04
119.11
116.88
53.30
43.35
180
170
160
150
140
130
120
110
100
90
80
70
60
50
40
30
20
10
ppm

Acquisition Parameter

Source Type	ESI	Ion Polarity	Positive	Set Nebulizer	0.3 Bar
Focus	Active	Set Capillary	4000 V	Set Dry Heater	240 °C
Scan Begin	100 m/z	Set End Plate Offset	-500 V	Set Dry Gas	4.0 l/min
Scan End	1000 m/z	Set Charging Voltage	2000 V	Set Divert Valve	Source
		Set Corona	0 nA	Set APCI Heater	0 °C

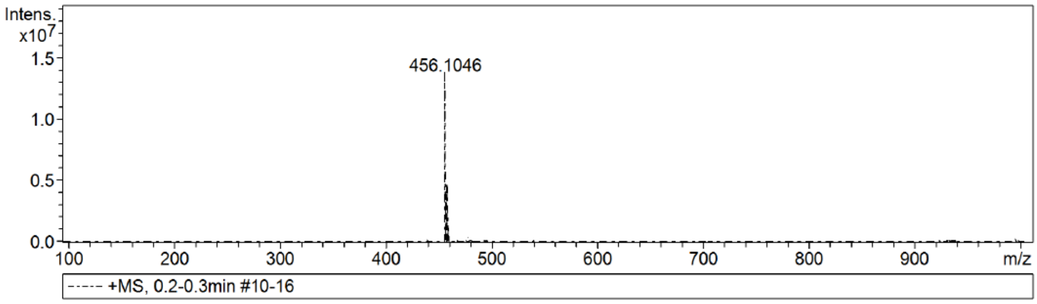

#	m/z	Res.	S/N	I	I %	FWHM
1	456.1046	47856	55239.7	13513015	100.0	0.0095
2	458.1018	46151	19753.1	4838887	35.8	0.0099

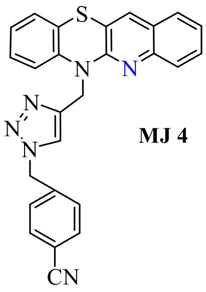

MJ 4

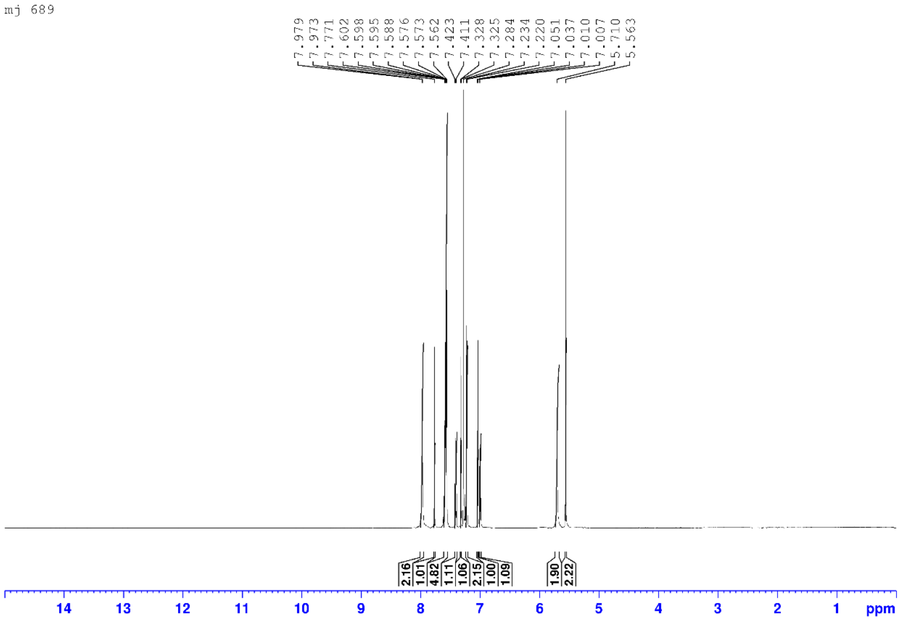

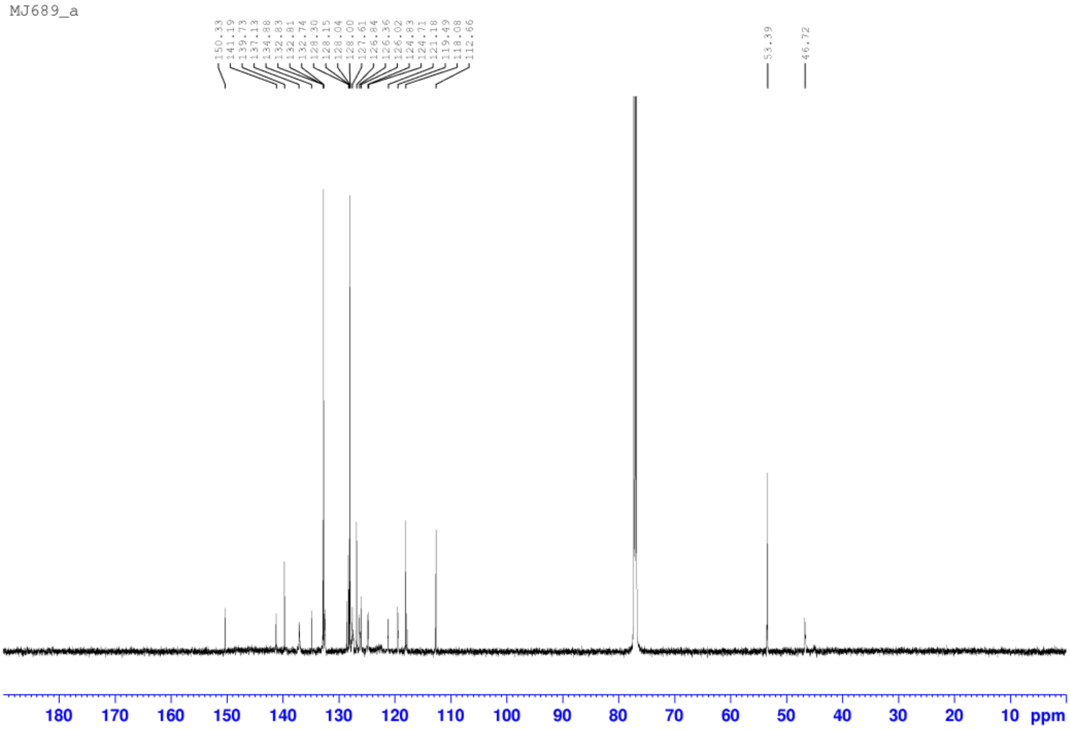
MJ689_a
150.33
141.19
139.73
137.13
134.88
132.83
132.81
132.74
128.30
128.15
128.04
128.00
127.61
126.84
126.36
126.02
124.83
124.71
121.18
119.49
118.08
112.66
53.39
46.72
180
170
160
150
140
130
120
110
100
90
80
70
60
50
40
30
20
10
ppm

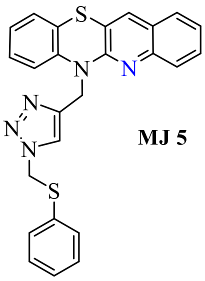
S
N
N
N
N
N
S

MJ 5

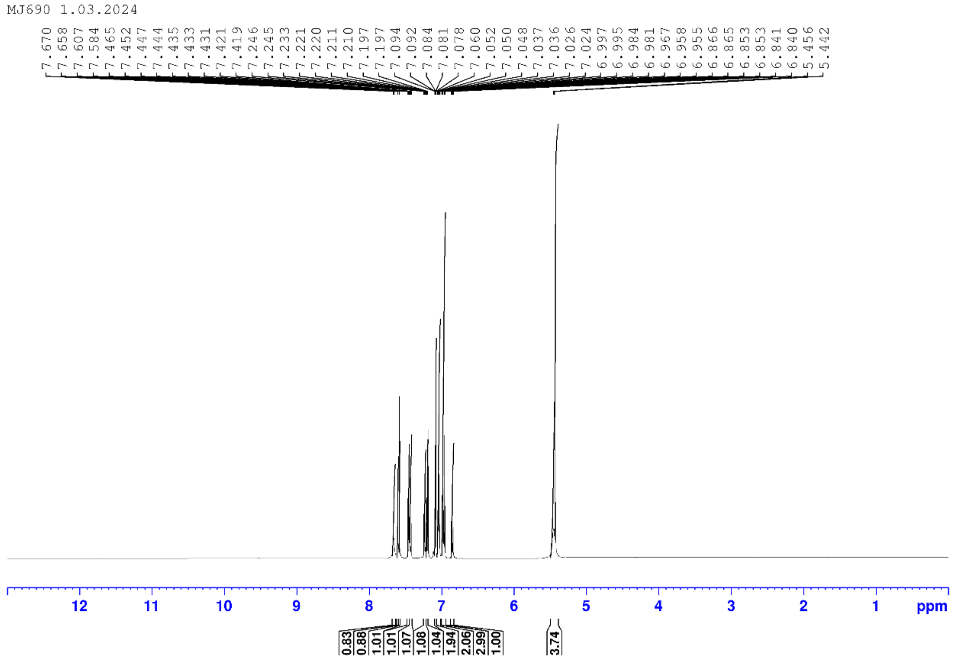
MJ690 1.03.2024
7.670 7.658 7.607 7.584 7.465 7.452 7.447 7.444 7.435 7.433 7.431 7.421 7.419 7.246 7.245 7.233 7.221 7.220 7.211 7.210 7.197 7.197 7.094 7.092 7.084 7.081 7.078 7.060 7.052 7.050 7.048 7.037 7.036 7.026 7.024 6.997 6.995 6.984 6.981 6.967 6.958 6.955 6.866 6.865 6.853 6.853 6.841 6.840 5.456 5.442
12 11 10 9 8 7 6 5 4 3 2 1 ppm
0.83 0.88 1.01 1.01 1.07 1.08 1.04 1.94 2.06 2.99 1.00 3.74

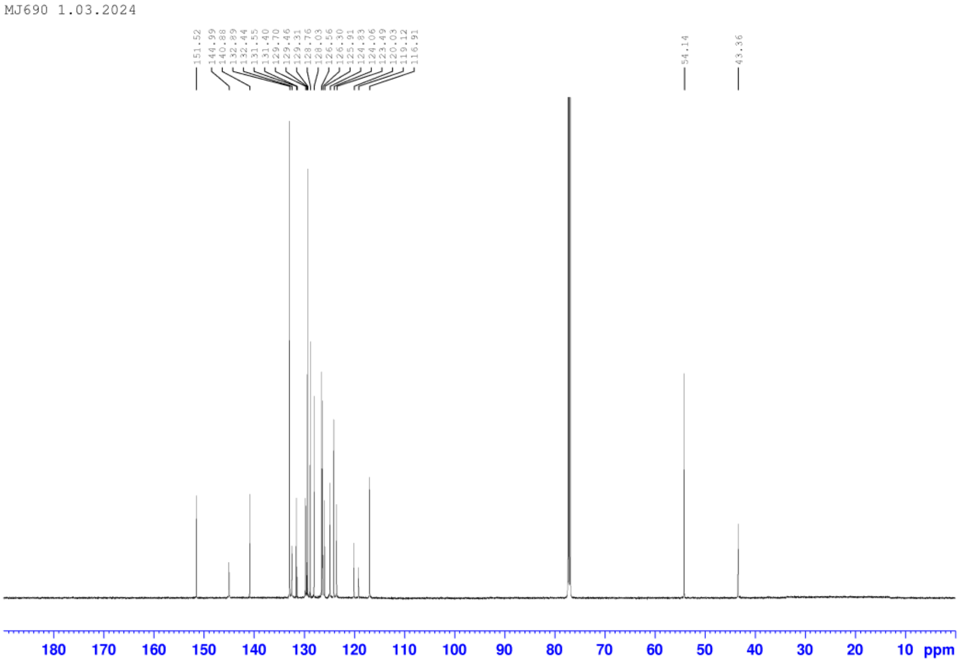
MJ690 1.03.2024
151.52 144.99 140.88 132.89 132.44 131.55 131.40 129.70 129.46 129.31 128.76 128.03 126.56 126.30 125.91 124.83 124.06 123.49 120.03 119.12 116.91
54.14
43.36
180 170 160 150 140 130 120 110 100 90 80 70 60 50 40 30 20 10 ppm

Acquisition Parameter

Source Type	ESI	Ion Polarity	Positive	Set Nebulizer	0.3 Bar
Focus	Active	Set Capillary	4000 V	Set Dry Heater	240 °C
Scan Begin	100 m/z	Set End Plate Offset	-500 V	Set Dry Gas	4.0 l/min
Scan End	1000 m/z	Set Charging Voltage	2000 V	Set Divert Valve	Source
		Set Corona	0 nA	Set APCI Heater	0 °C

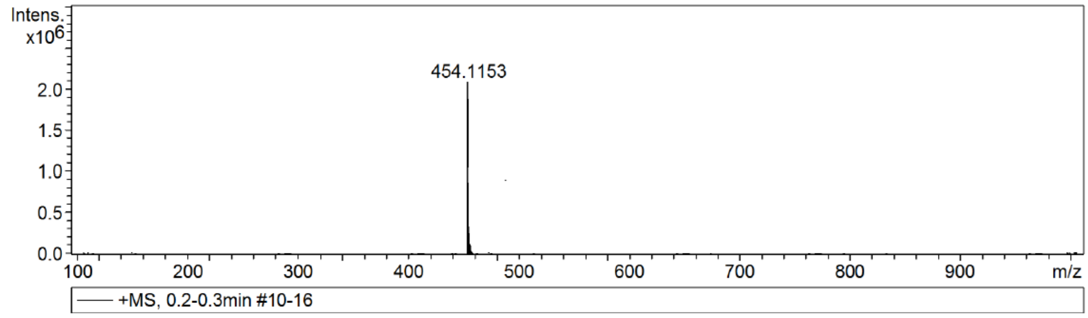

#	m/z	Res.	S/N	I	I %	FWHM
1	454.1153	40072	2649.4	1074968	100.0	0.0113
2	456.1115	22001	322.2	131407	12.2	0.0207

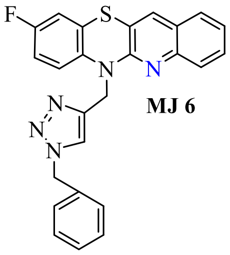

MJ 6

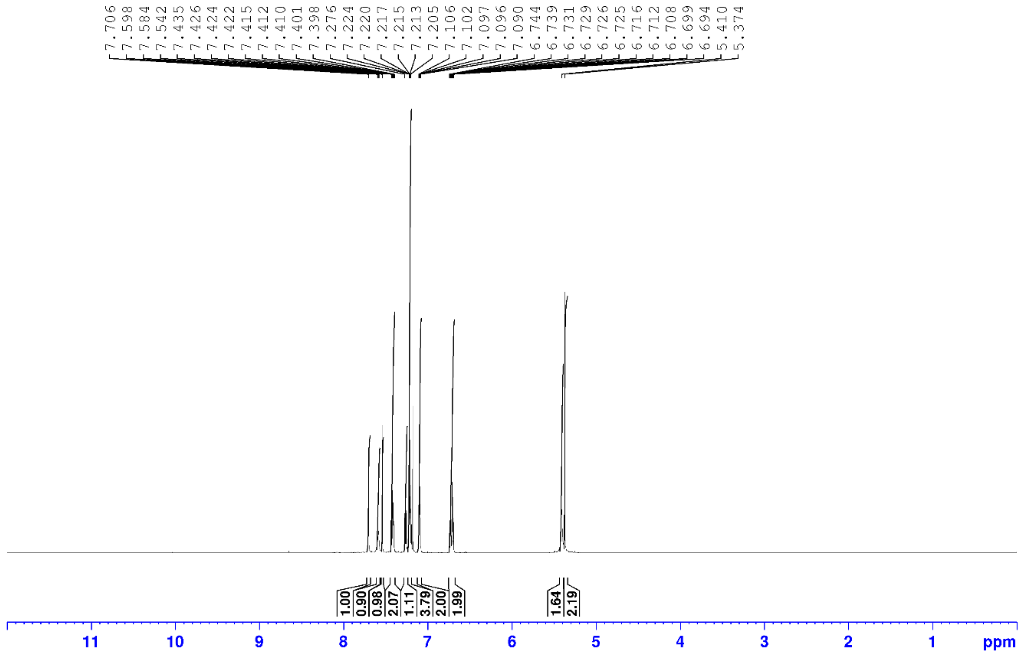

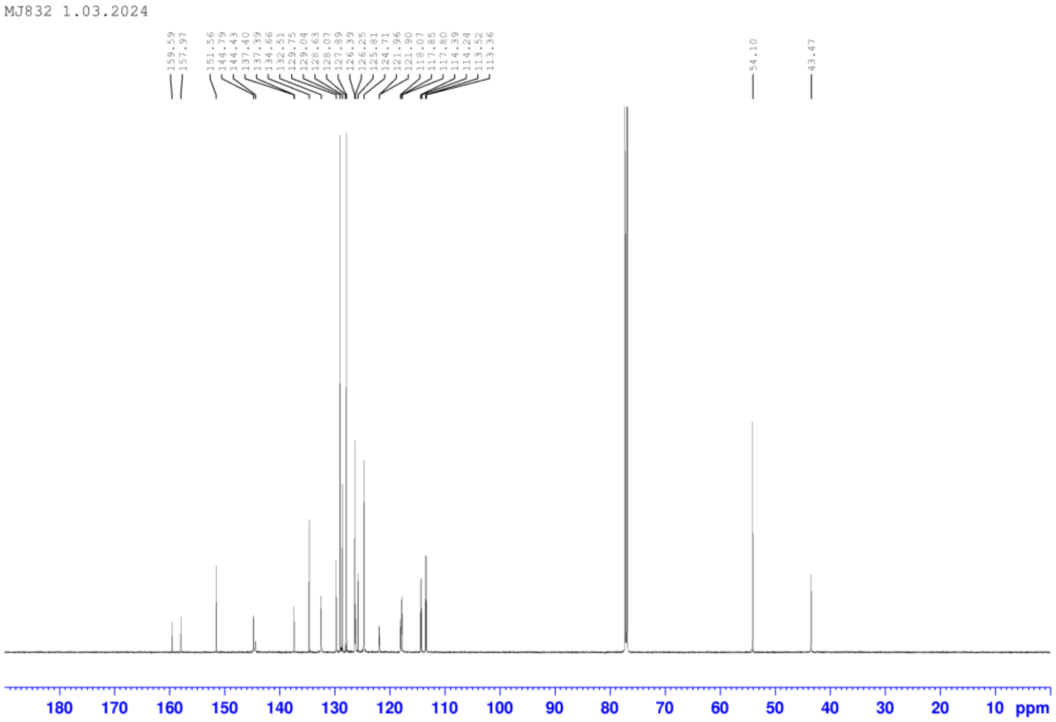

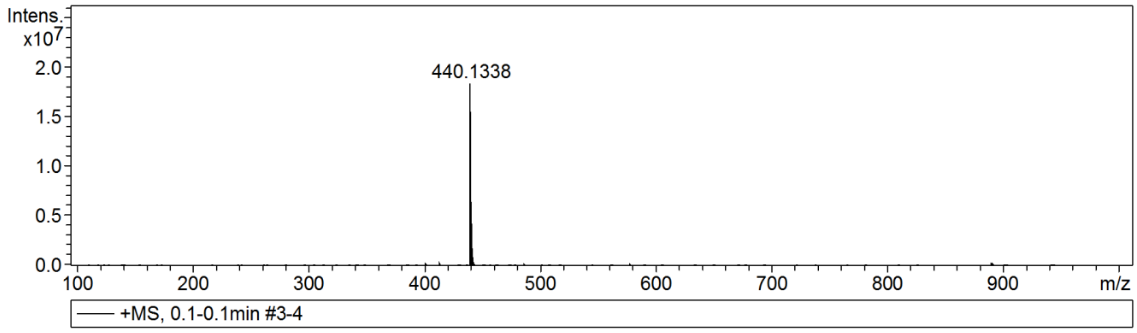

#	m/z	Res.	S/N	I	I %	FWHM
1	440.1338	43039	29180.8	18398392	100.0	0.0102

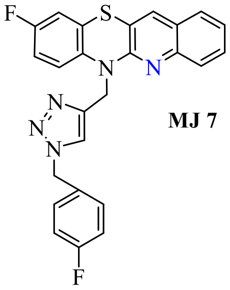

MJ 7

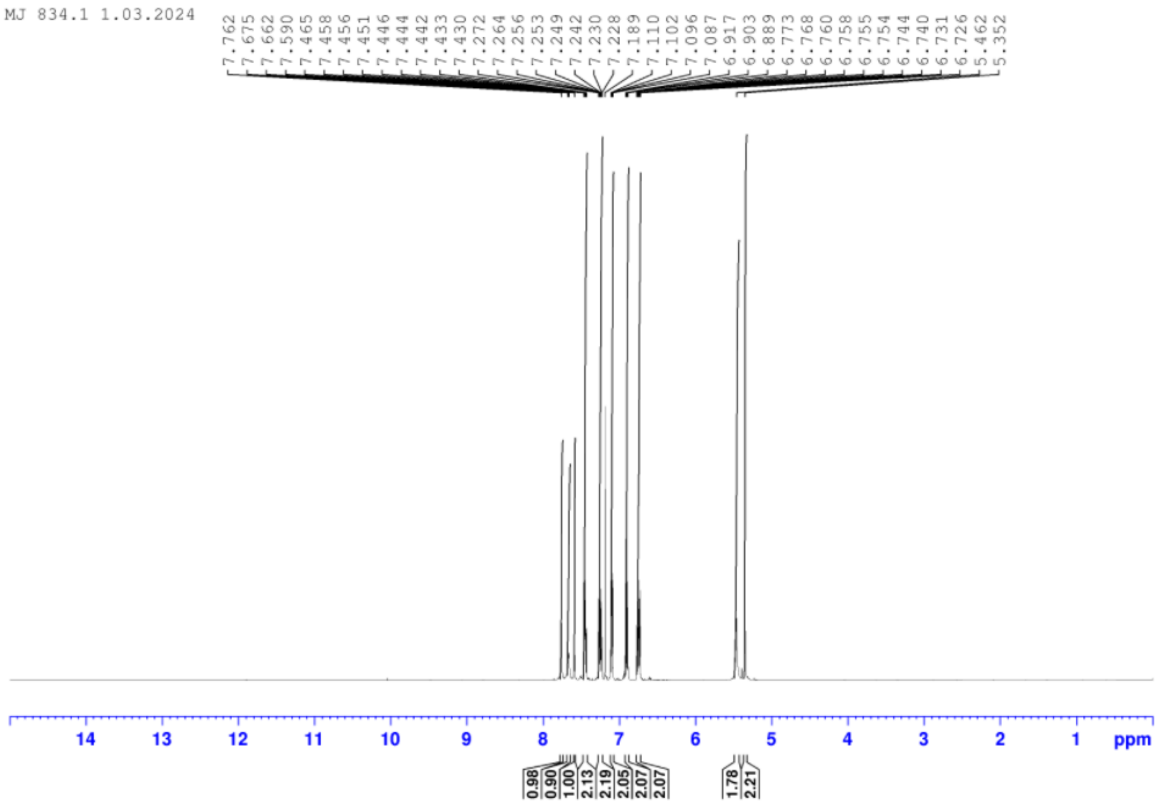
MJ 834.1 1.03.2024
7.762
7.675
7.662
7.590
7.465
7.458
7.456
7.451
7.446
7.444
7.442
7.433
7.430
7.272
7.264
7.256
7.253
7.249
7.242
7.230
7.228
7.189
7.110
7.102
7.096
7.087
6.917
6.903
6.889
6.773
6.768
6.760
6.758
6.755
6.754
6.744
6.740
6.731
6.726
5.462
5.352
0.98
0.90
1.00
2.13
2.19
2.05
2.07
2.07
1.78
2.21
14
13
12
11
10
9
8
7
6
5
4
3
2
1
ppm

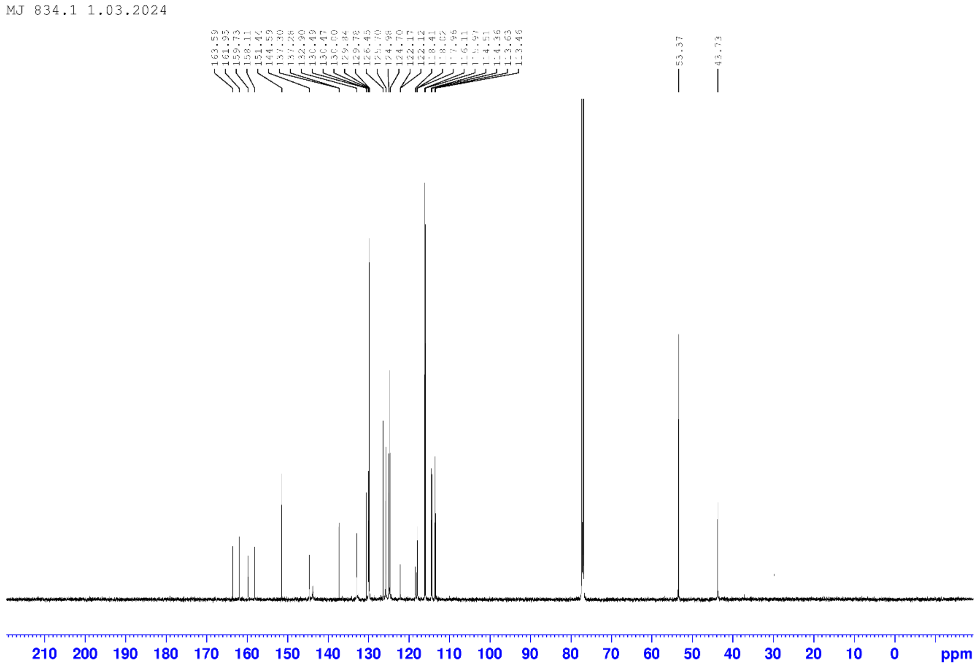
MJ 834.1 1.03.2024
163.59
161.95
159.73
158.11
151.44
144.59
137.30
137.28
132.90
130.49
130.47
130.00
129.84
129.78
126.45
125.70
124.98
124.70
122.17
122.12
118.41
118.02
117.96
116.11
115.97
114.51
114.36
113.63
113.46
53.37
43.73
210
200
190
180
170
160
150
140
130
120
110
100
90
80
70
60
50
40
30
20
10
0
ppm

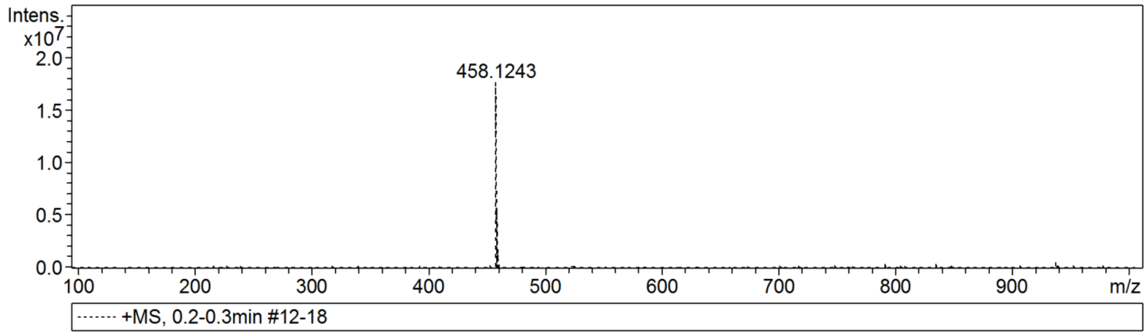

#	m/z	Res.	S/N	I	I %	FWHM
1	458.1243	41719	31132.9	17594376	100.0	0.0110
2	459.1273	43285	8910.7	5043899	28.7	0.0106

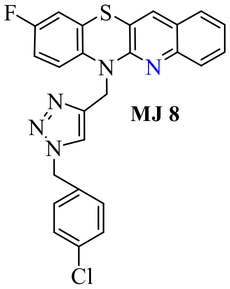

MJ 8

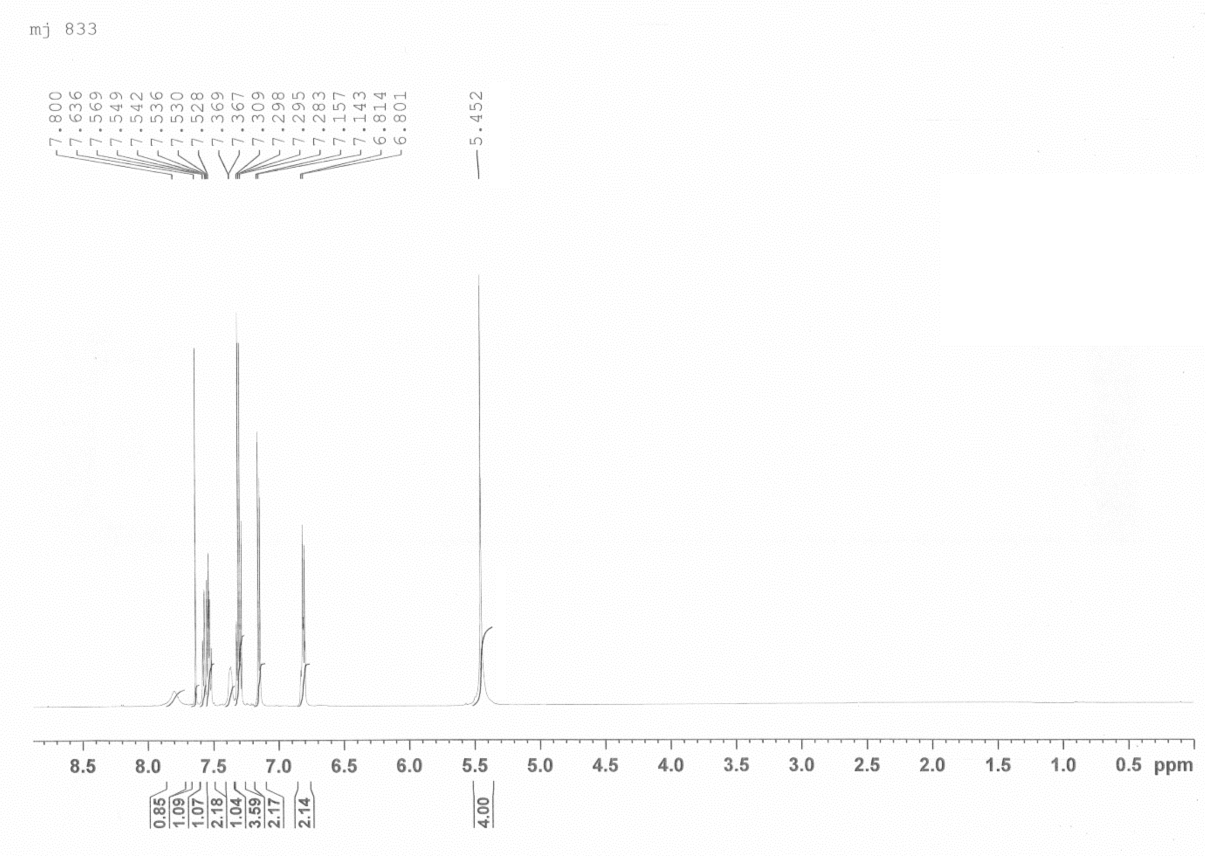

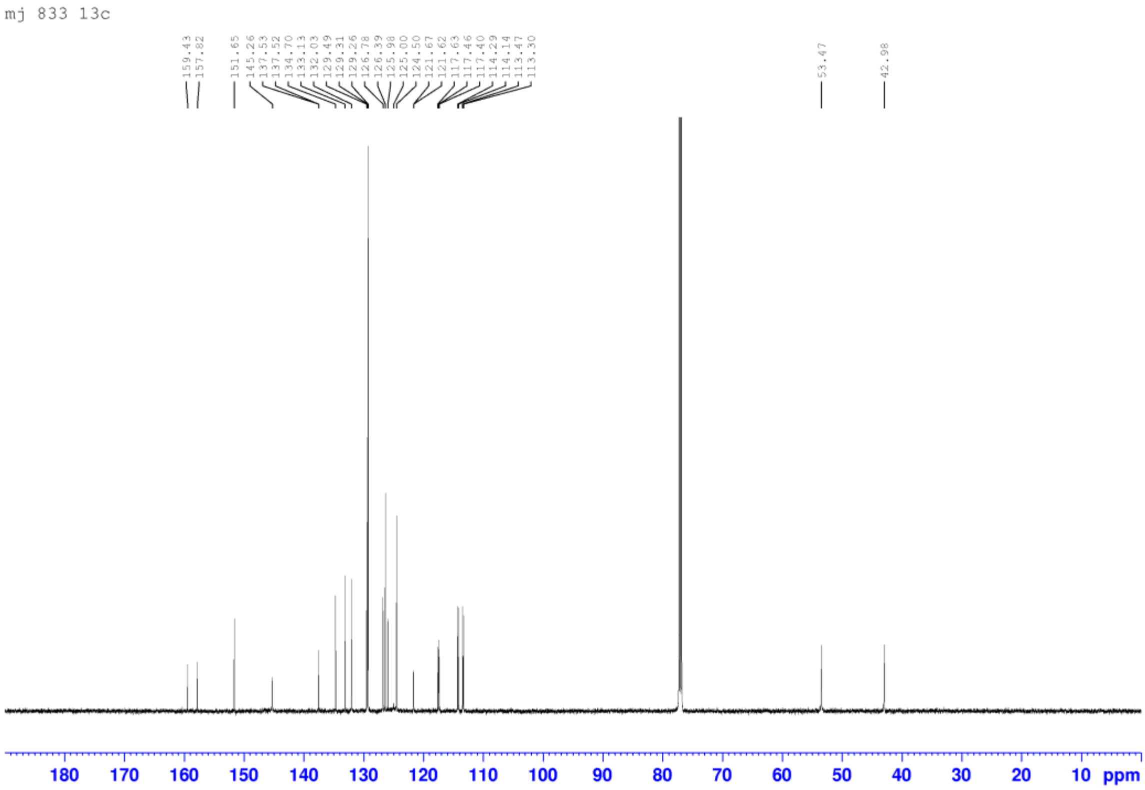

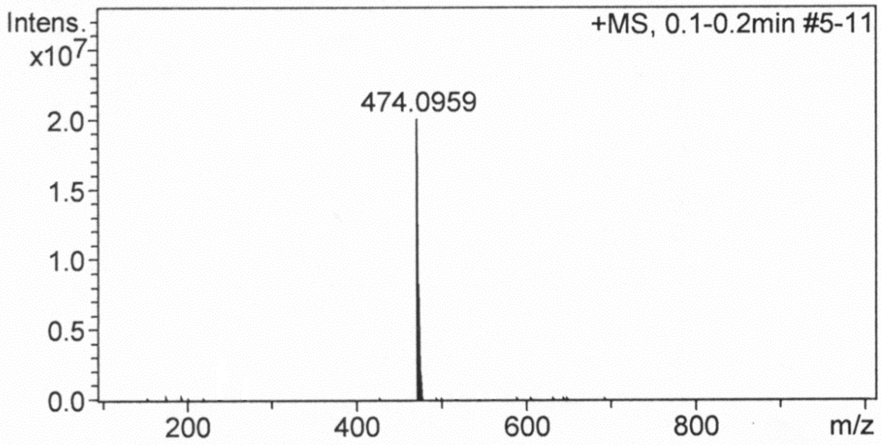

#	m/z	Res.	S/N	I	I %	FWHM
1	474.0959	29916	41379.5	20089818	100.0	0.0158
2	476.0931	44522	17018.9	8264077	41.1	0.0107

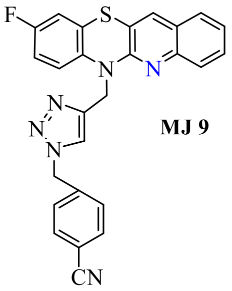

MJ 9

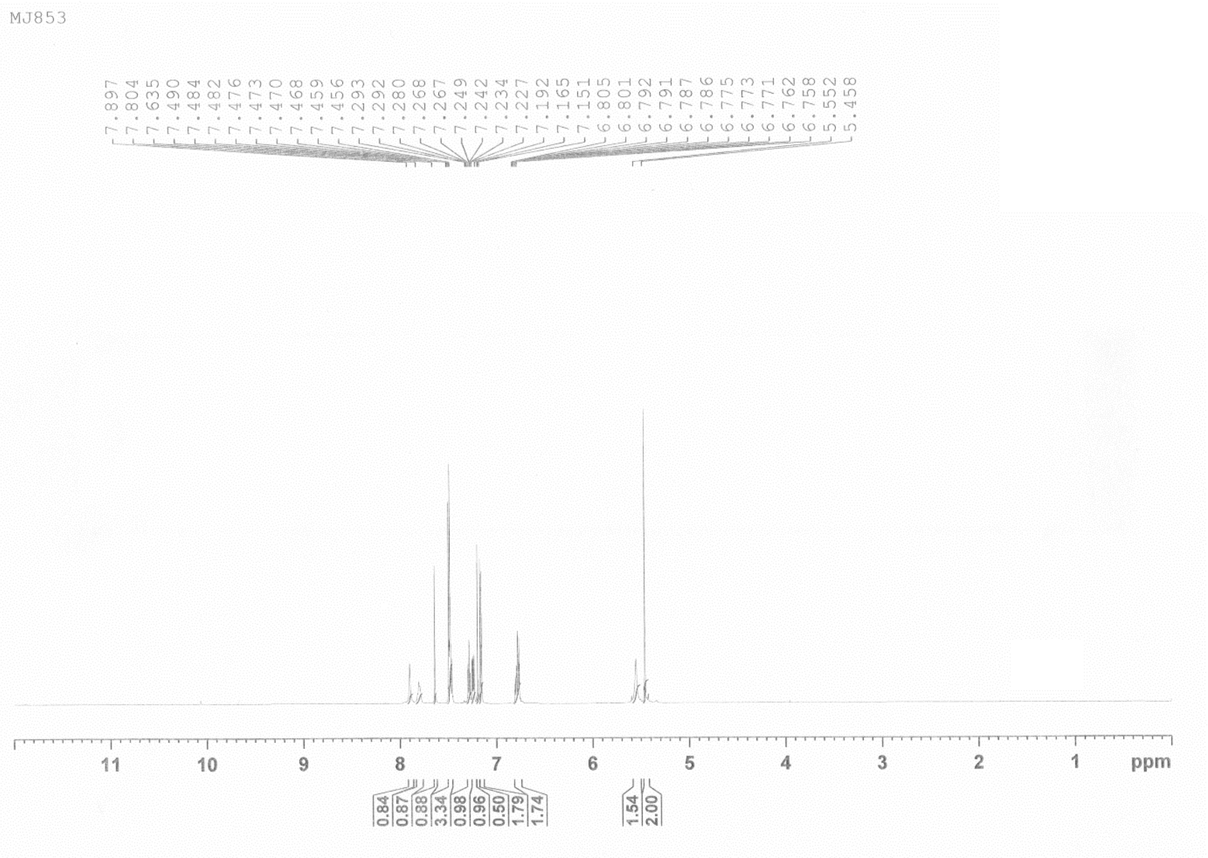
MJ853
7.897
7.804
7.635
7.490
7.484
7.482
7.476
7.473
7.470
7.468
7.459
7.456
7.293
7.292
7.280
7.268
7.267
7.249
7.242
7.234
7.227
7.192
7.165
7.151
6.805
6.801
6.792
6.791
6.787
6.786
6.775
6.773
6.771
6.762
6.758
5.552
5.458
11 10 9 8 7 6 5 4 3 2 1 ppm
0.84
0.87
0.88
3.34
0.98
0.96
0.50
1.79
1.74
1.54
2.00

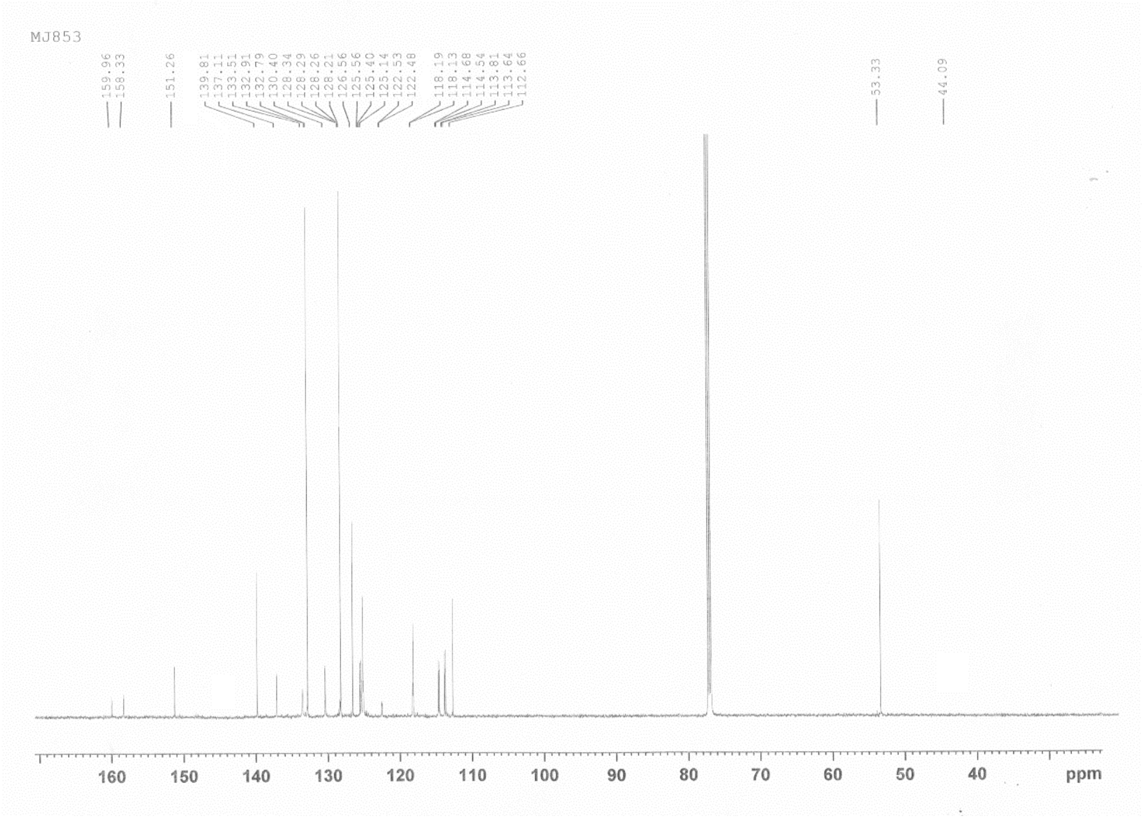

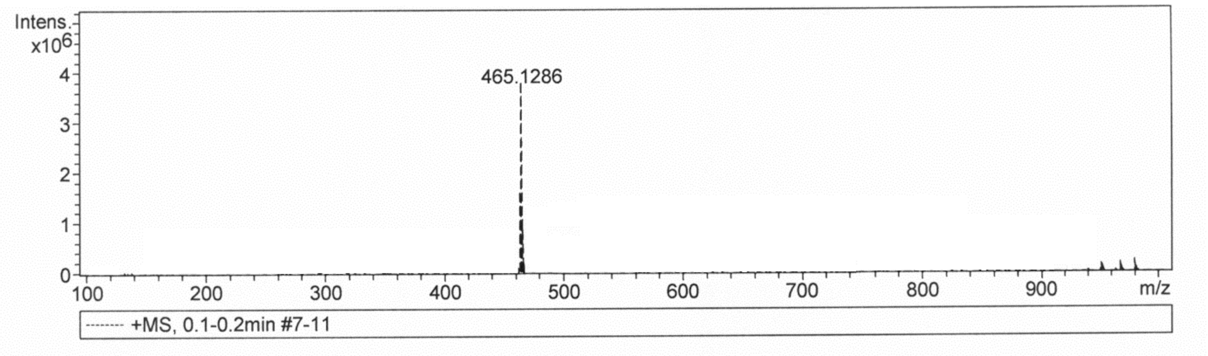

#	m/z	Res.	S/N	I	I %	FWHM
1	465.1286	41059	4474.9	3685459	100.0	0.0113
2	466.1316	30847	1206.0	993290	27.0	0.0151

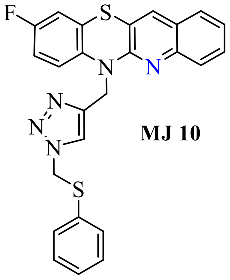

MJ 10

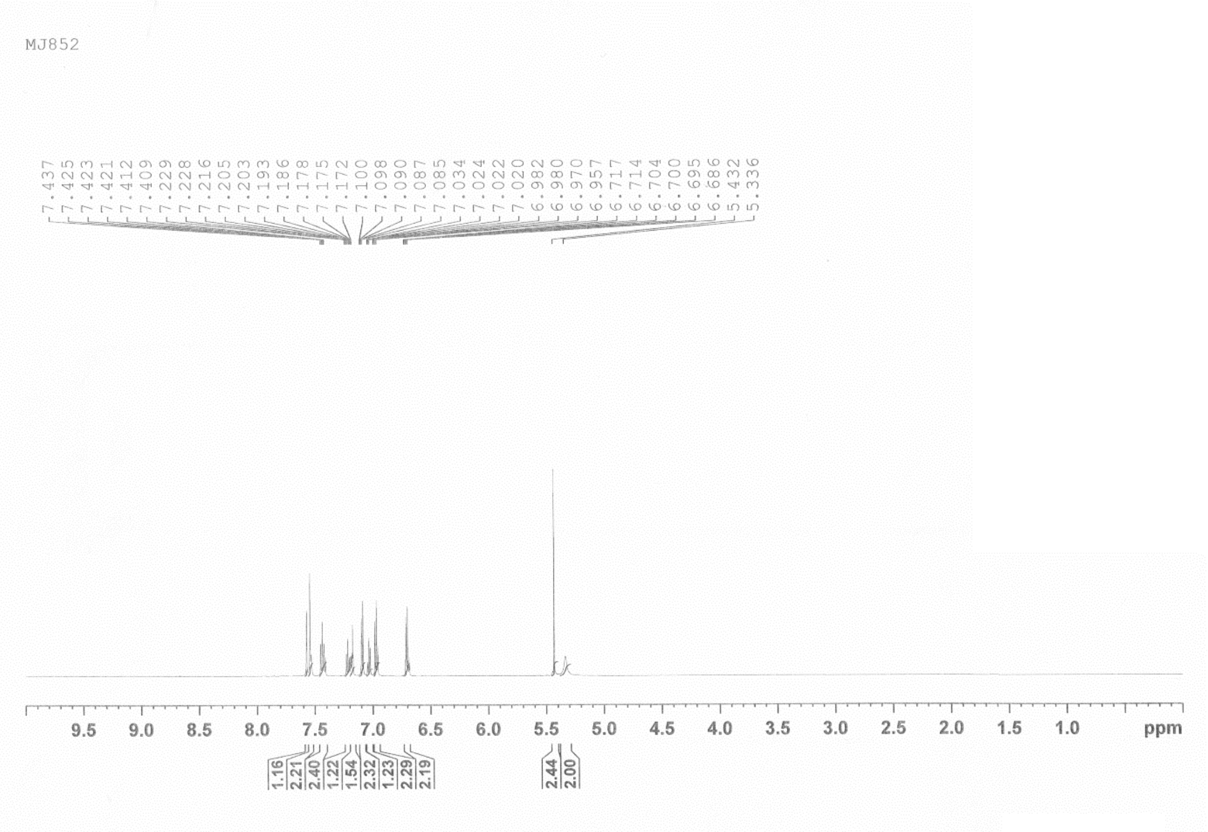
MJ852
ppm

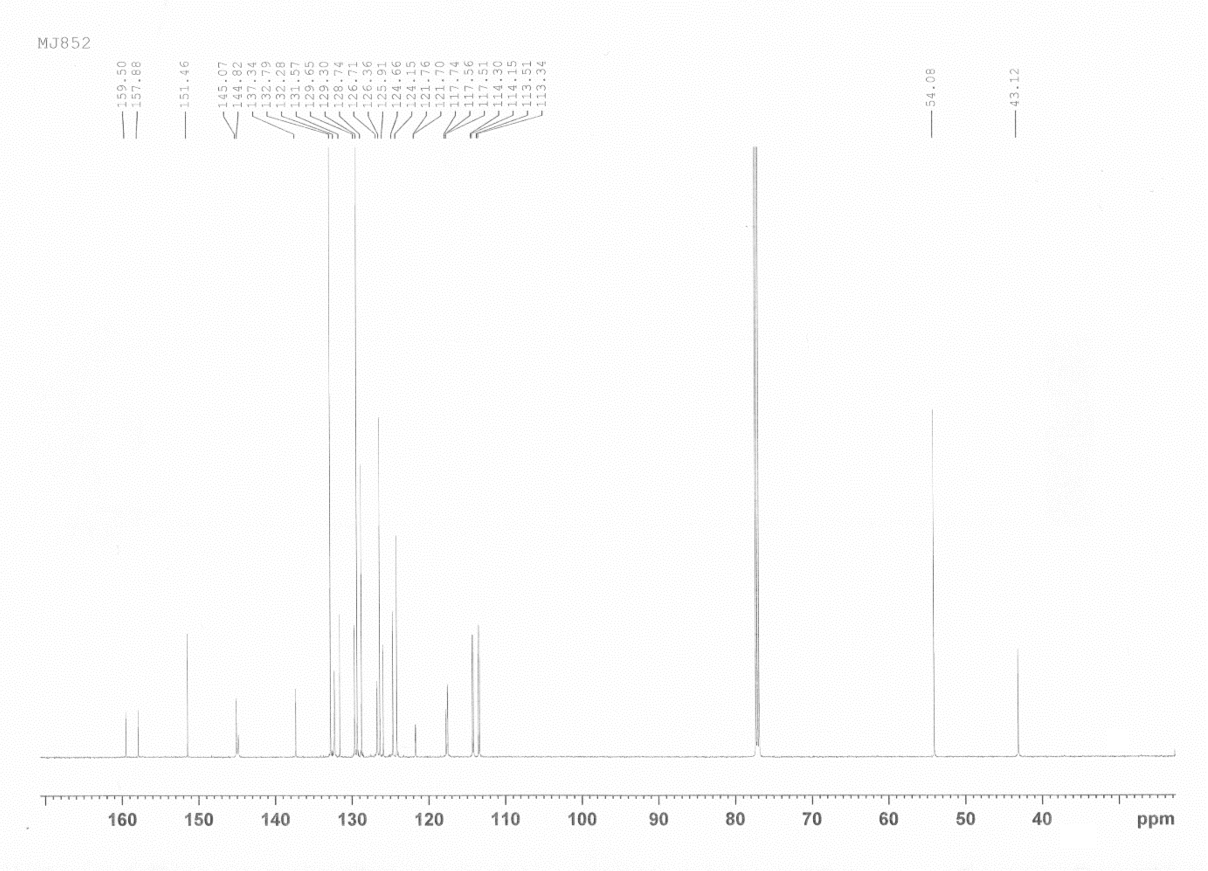
MJ852
ppm

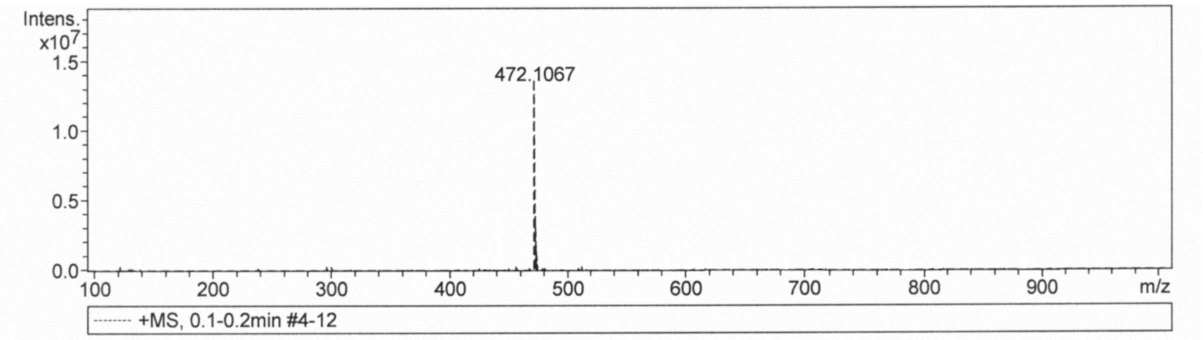

#	m/z	Res.	S/N	I	I %	FWHM
1	472.1067	46868	13439.5	13213756	100.0	0.0101

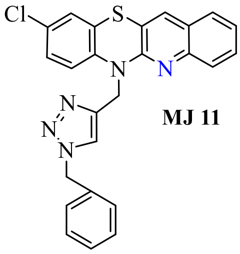

MJ 11

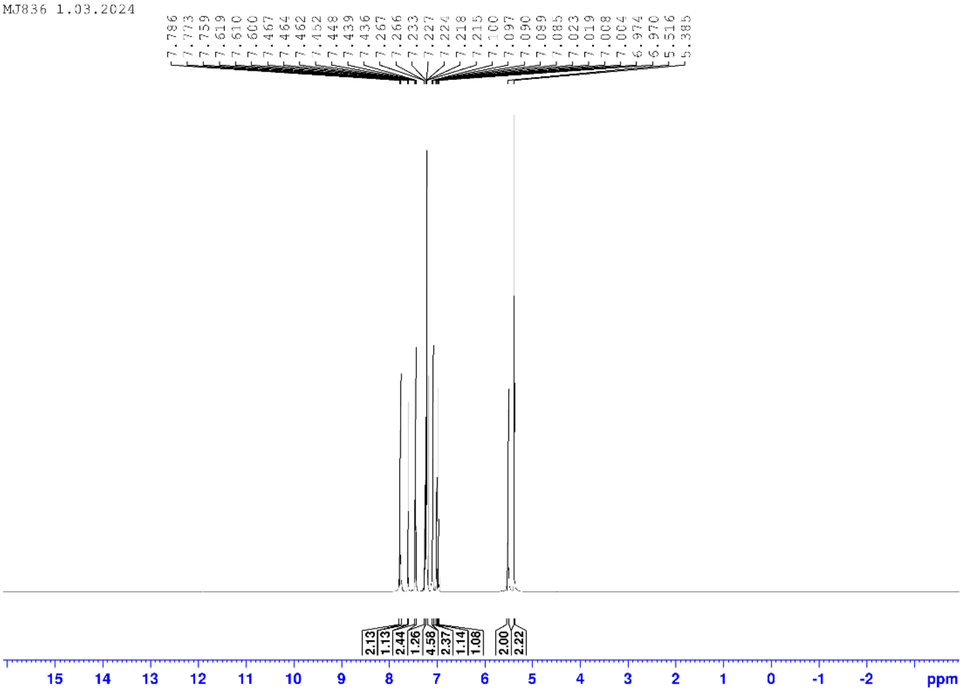

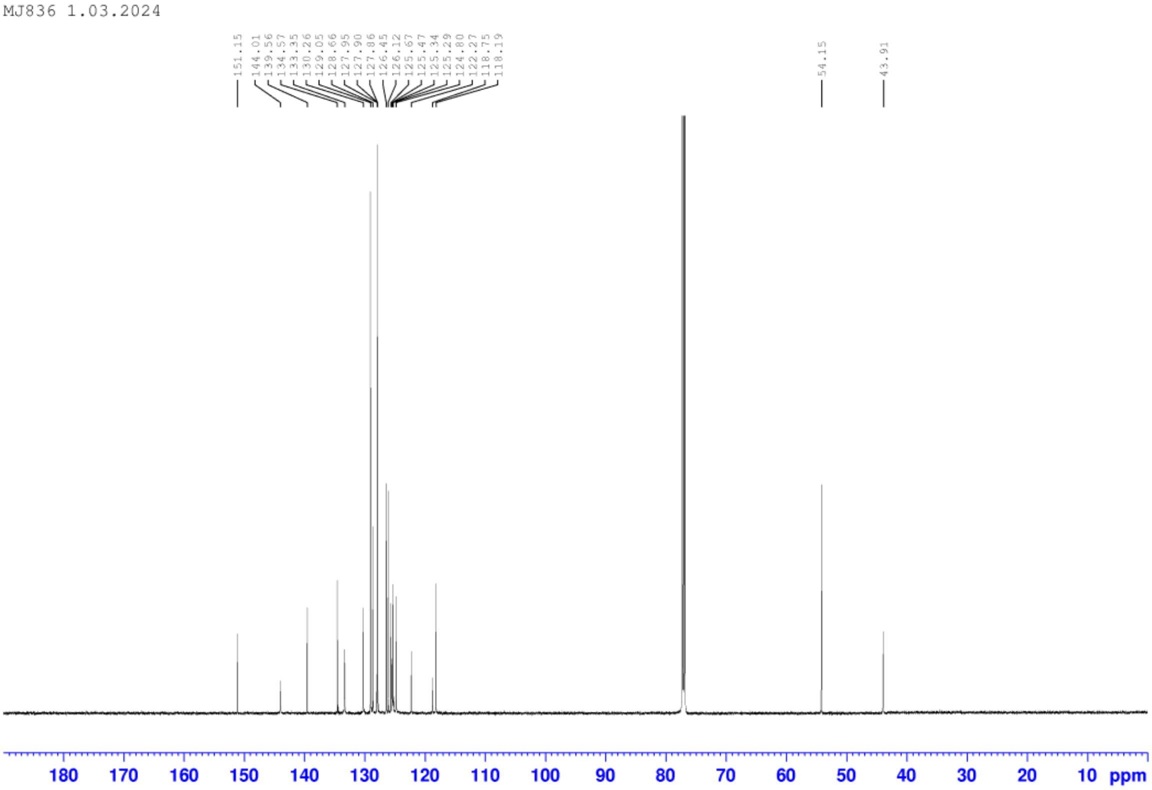

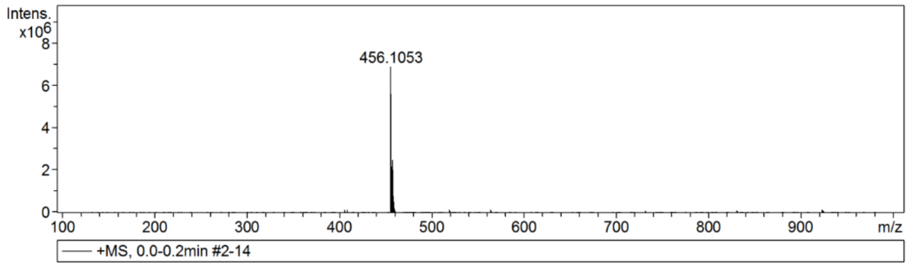

#	m/z	Res.	S/N	I	I %	FWHM
1	456.1053	43666	12303.3	6880326	100.0	0.0104

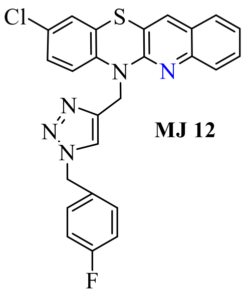

MJ 12

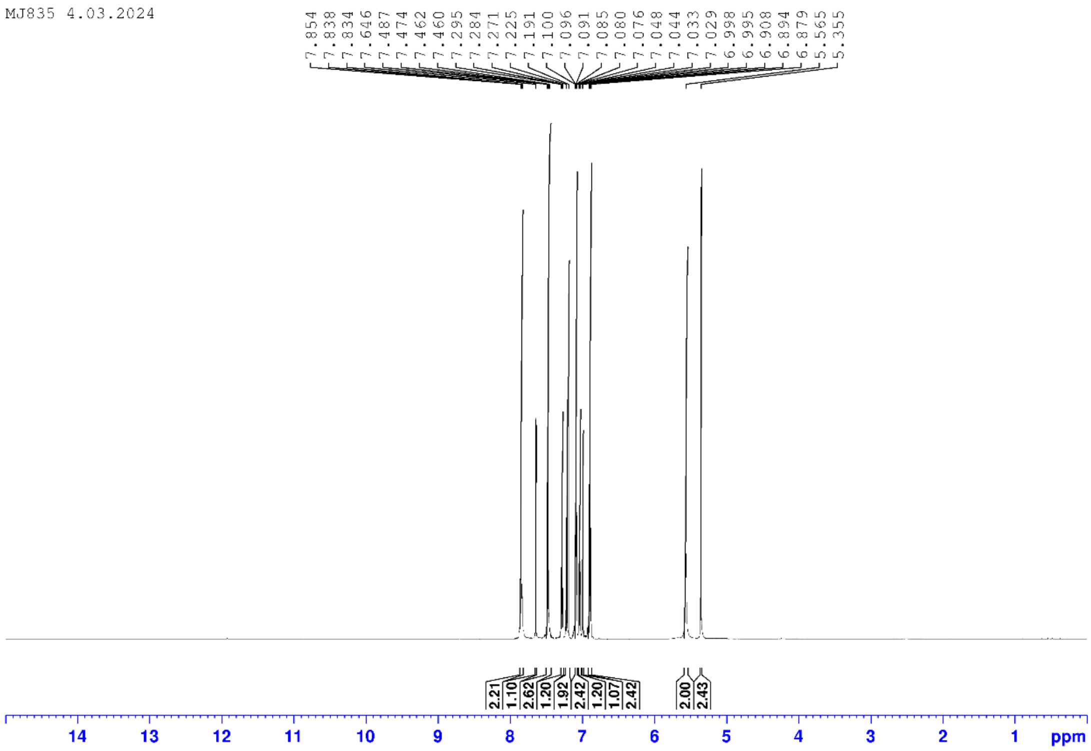
MJ835 4.03.2024
7.854
7.838
7.834
7.646
7.487
7.474
7.462
7.460
7.295
7.284
7.271
7.225
7.191
7.100
7.096
7.091
7.085
7.080
7.076
7.048
7.044
7.033
7.029
6.998
6.995
6.908
6.894
6.879
5.565
5.355
2.21
1.10
2.62
1.20
1.92
2.42
1.20
1.07
2.42
2.00
2.43
14
13
12
11
10
9
8
7
6
5
4
3
2
1
ppm

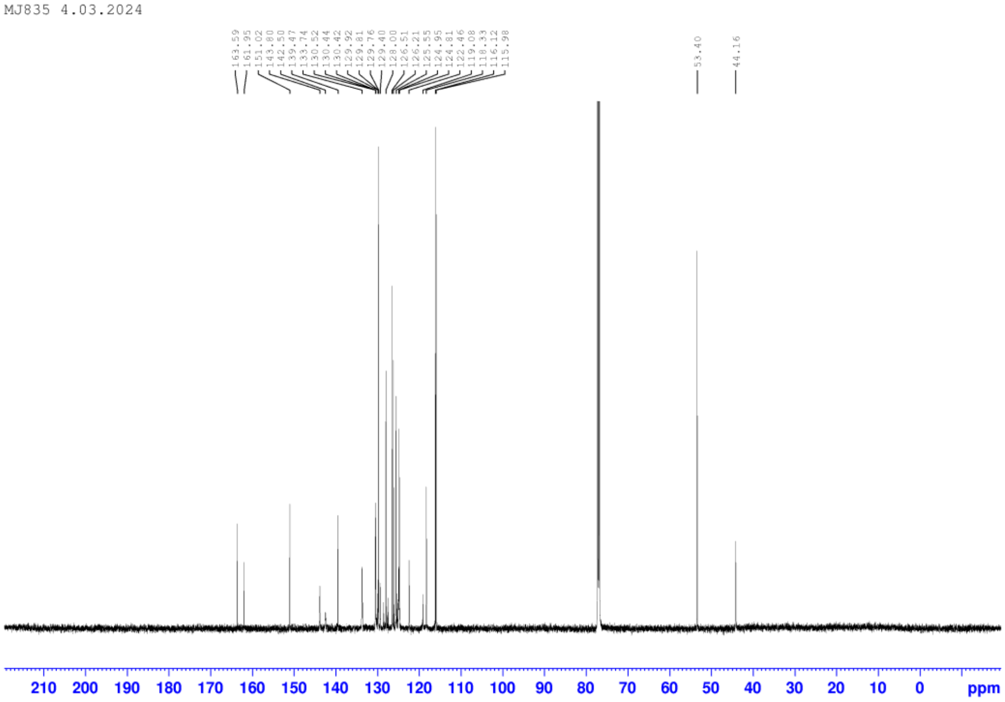
MJ835 4.03.2024
163.59
161.95
151.02
143.80
142.50
139.47
133.74
130.52
130.44
130.42
129.92
129.81
129.76
129.40
128.00
126.51
126.21
125.55
124.95
124.81
122.46
119.08
118.33
116.12
115.98
53.40
44.16
210
200
190
180
170
160
150
140
130
120
110
100
90
80
70
60
50
40
30
20
10
0
ppm

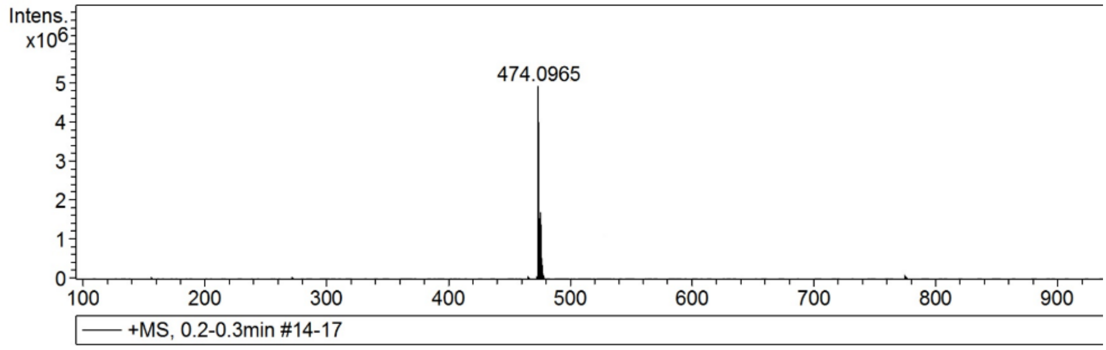

#	m/z	Res.	S/N	I	I %	FWHM
1	474.0965	43894	6620.6	4905955	100.0	0.0108
2	476.0943	35343	2303.4	1711240	34.9	0.0135

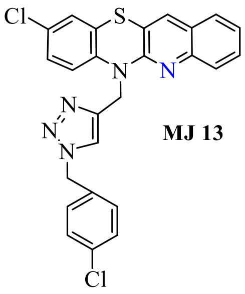

MJ 13

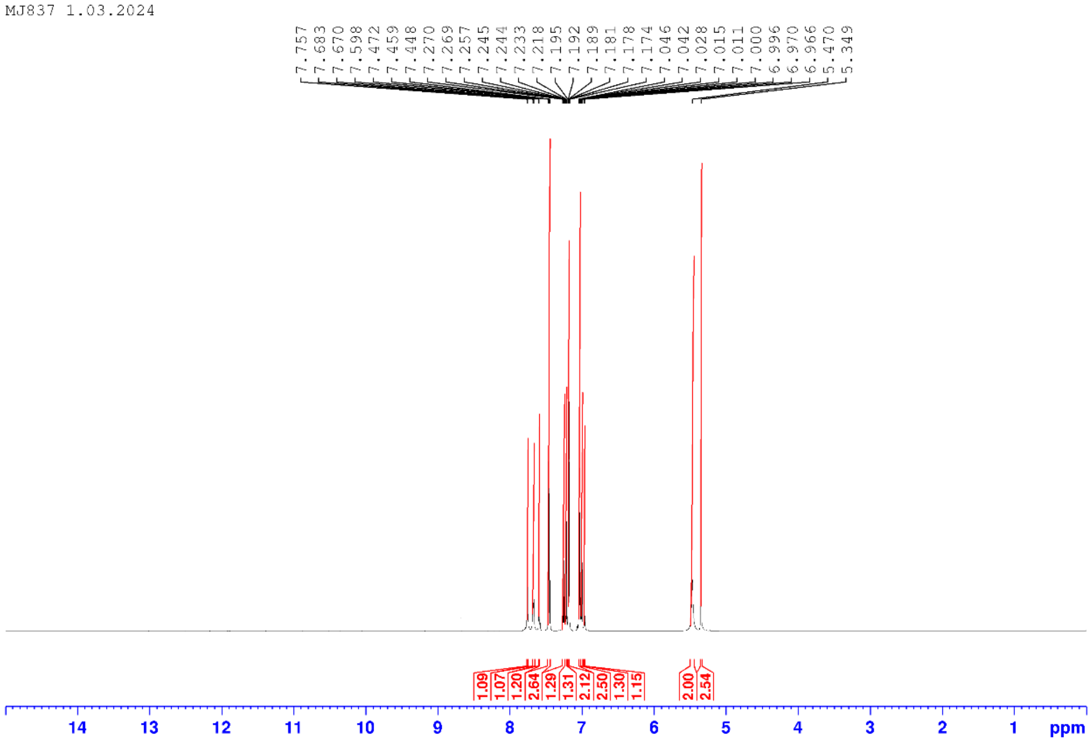

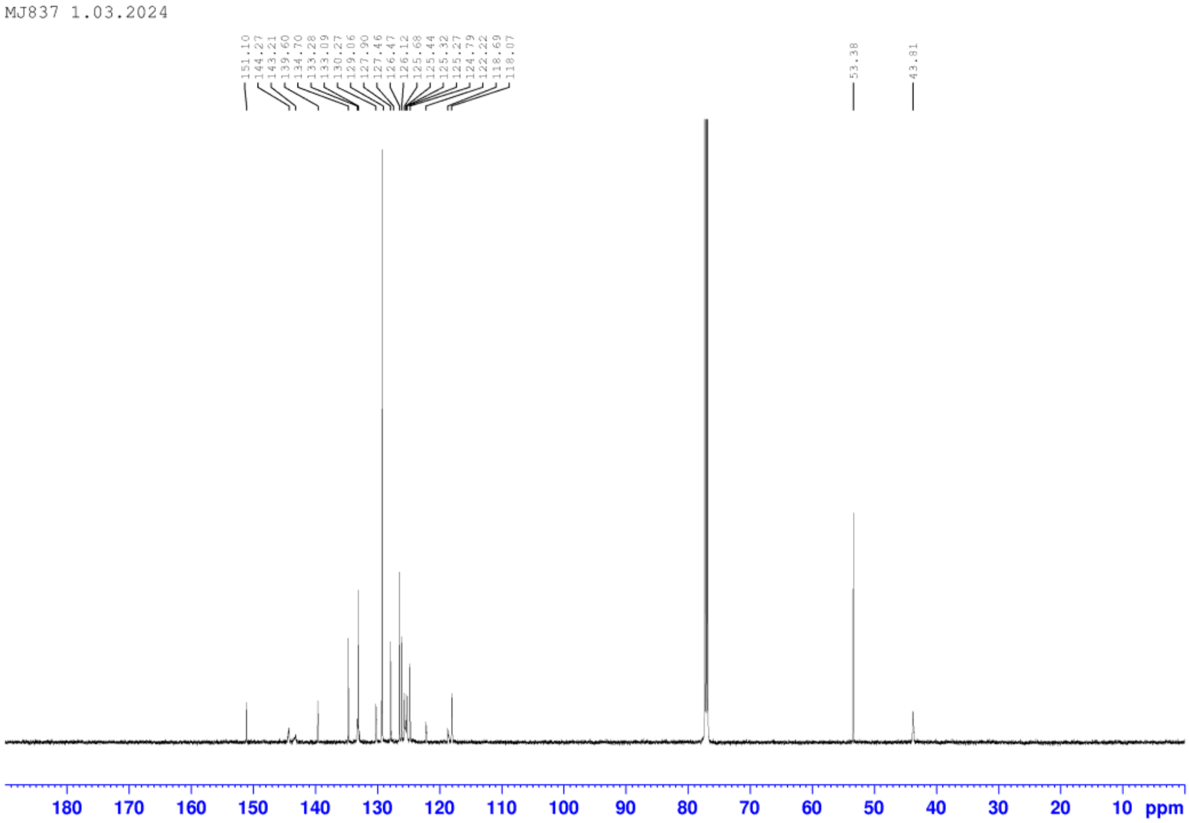

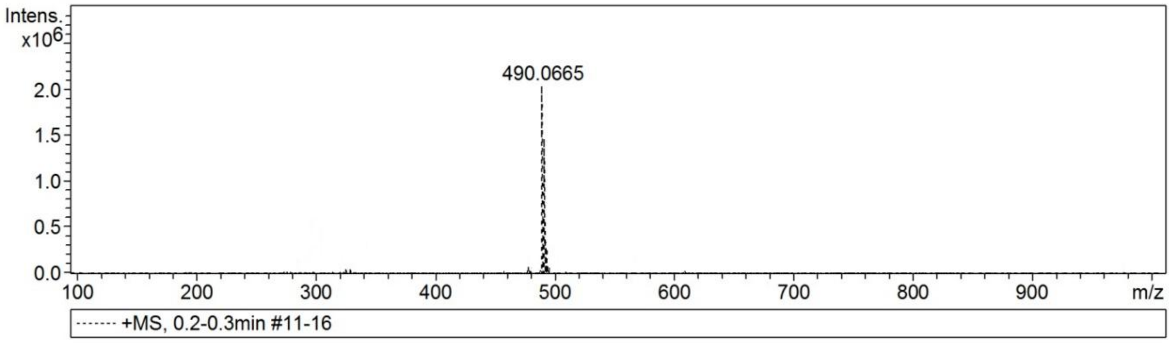

#	m/z	Res.	S/N	I	I %	FWHM
1	490.0665	38620	1631.4	2044084	100.0	0.0127
2	492.0580	35567	1121.2	1412107	69.1	0.0138

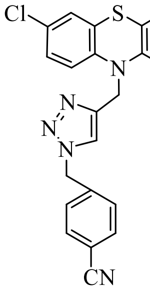

MJ 14

MJ855

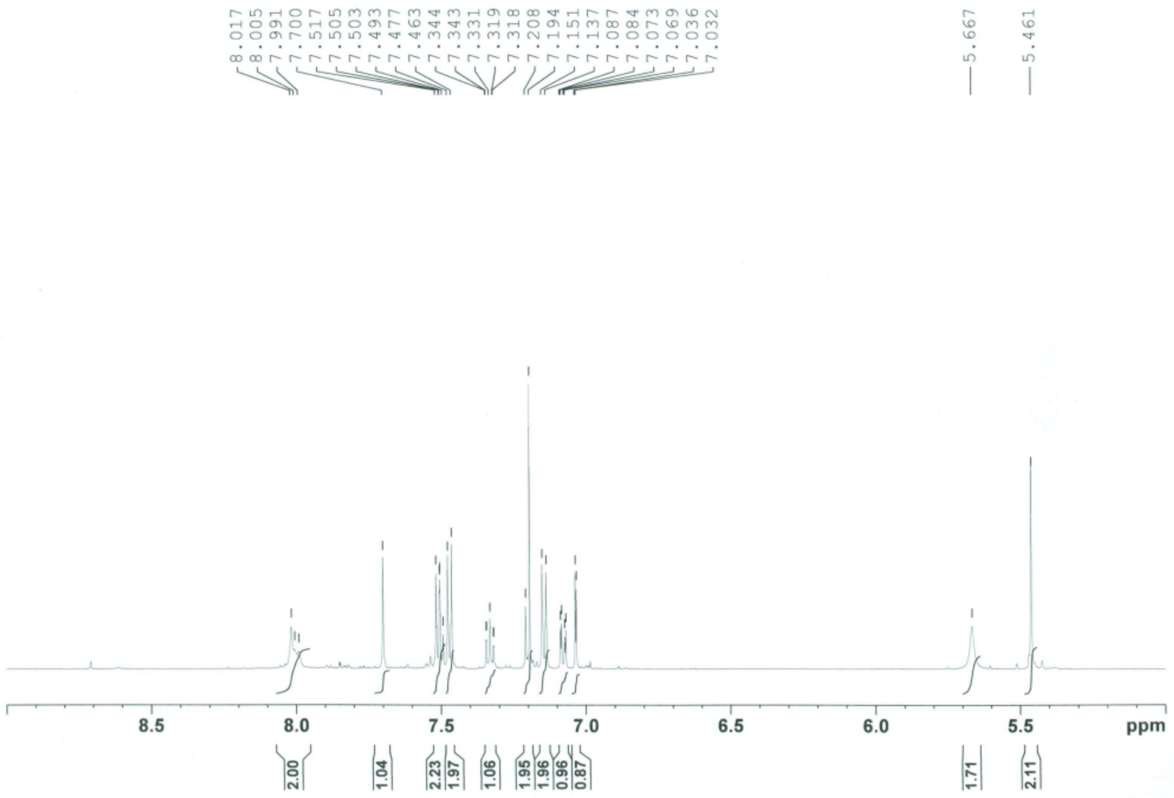
8.017
8.005
7.991
7.700
7.517
7.505
7.503
7.493
7.477
7.463
7.344
7.343
7.331
7.319
7.318
7.208
7.194
7.151
7.137
7.087
7.084
7.073
7.069
7.036
7.032
5.667
5.461
8.5
8.0
7.5
7.0
6.5
6.0
5.5
ppm
2.00
1.04
2.23
1.97
1.06
1.95
1.96
0.96
0.87
1.71
2.11

MJ855

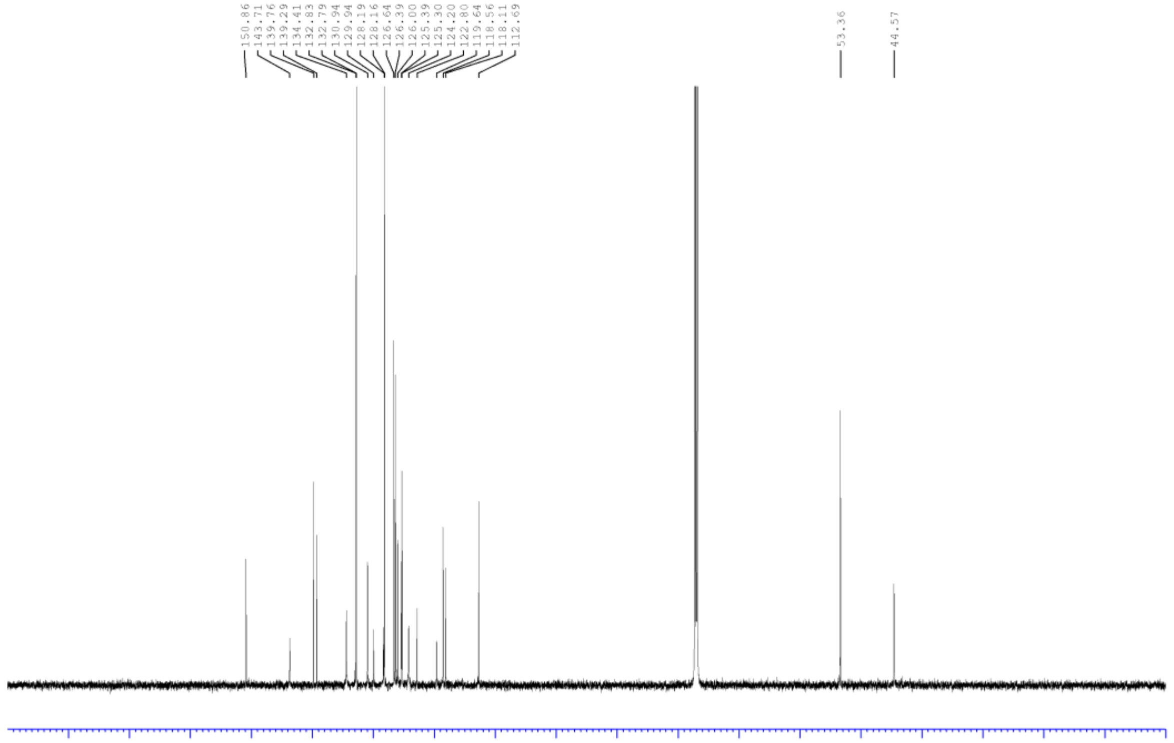
150.86
143.71
139.76
139.29
134.41
132.83
132.79
130.94
129.94
128.19
128.16
126.64
126.39
126.00
125.39
125.30
124.20
122.80
119.64
118.56
118.11
112.69
53.36
44.57
180
170
160
150
140
130
120
110
100
90
80
70
60
50
40
30
20
10
ppm

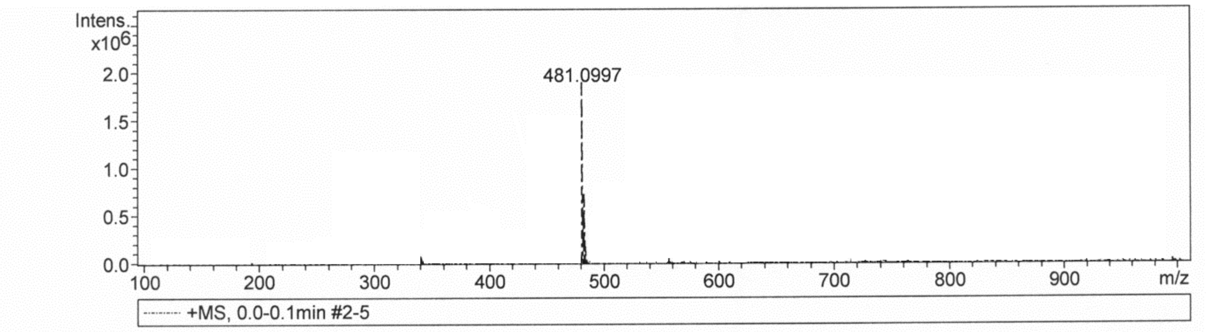

#	m/z	Res.	S/N	I	I %	FWHM
1	481.0997	36515	1459.2	1854323	100.0	0.0132
2	483.0977	27983	544.4	695440	37.5	0.0173

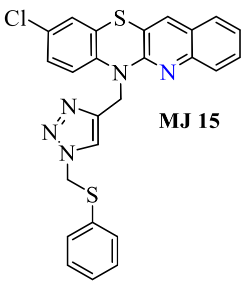

MJ 15

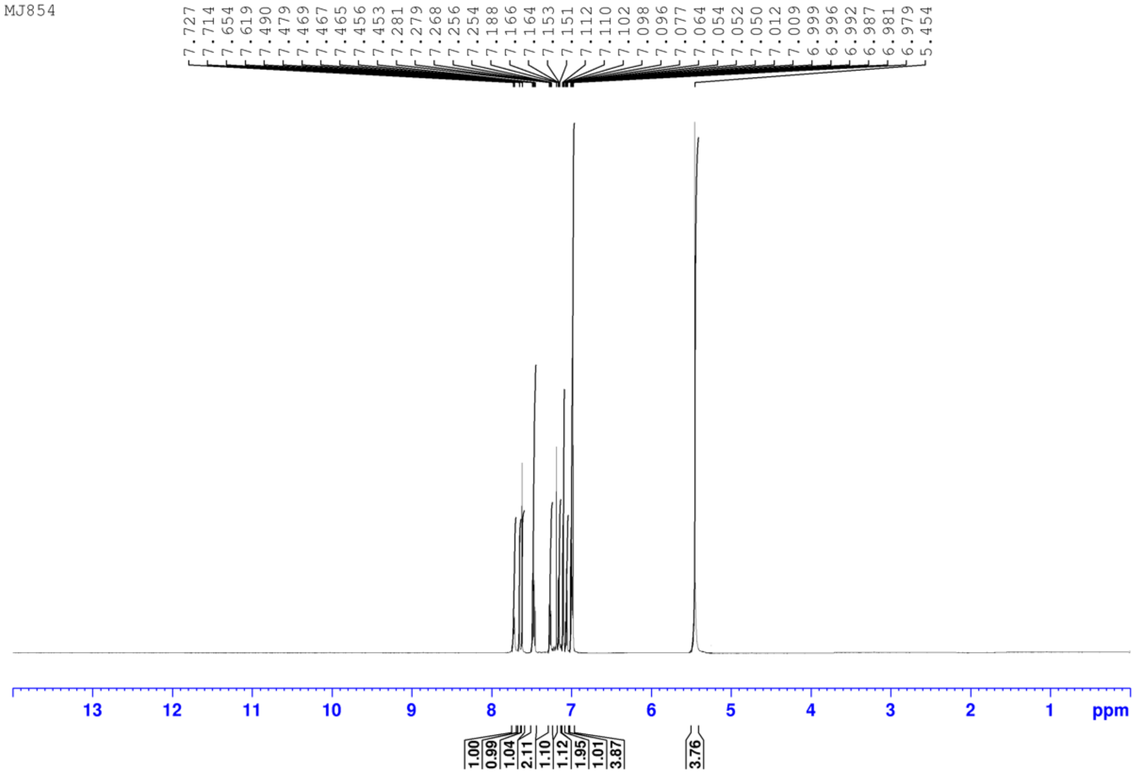

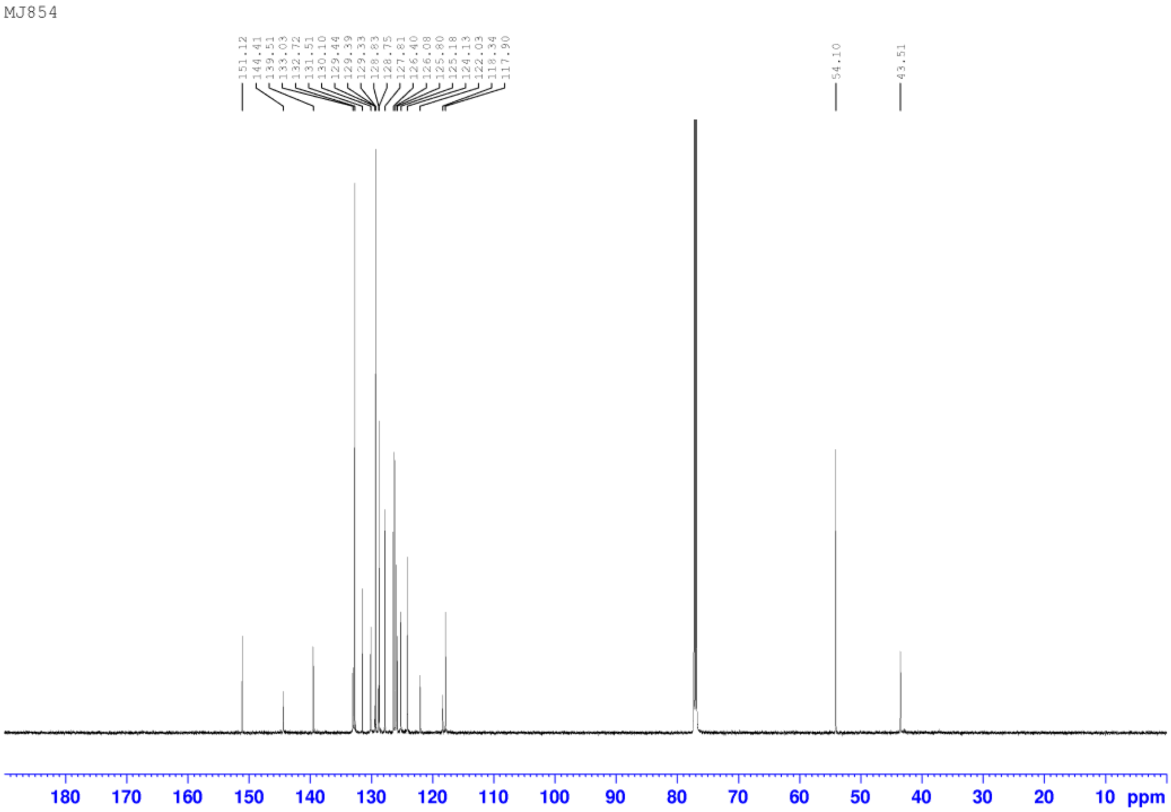

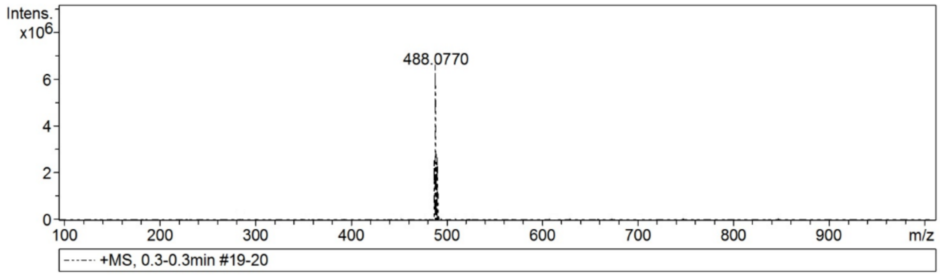

#	m/z	Res.	S/N	I	I %	FWHM
1	488.0770	41547	10221.6	6447837	100.0	0.0117
2	490.0716	34199	4048.1	2556067	39.6	0.0143

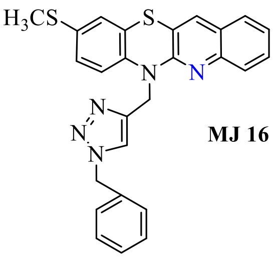

MJ 16

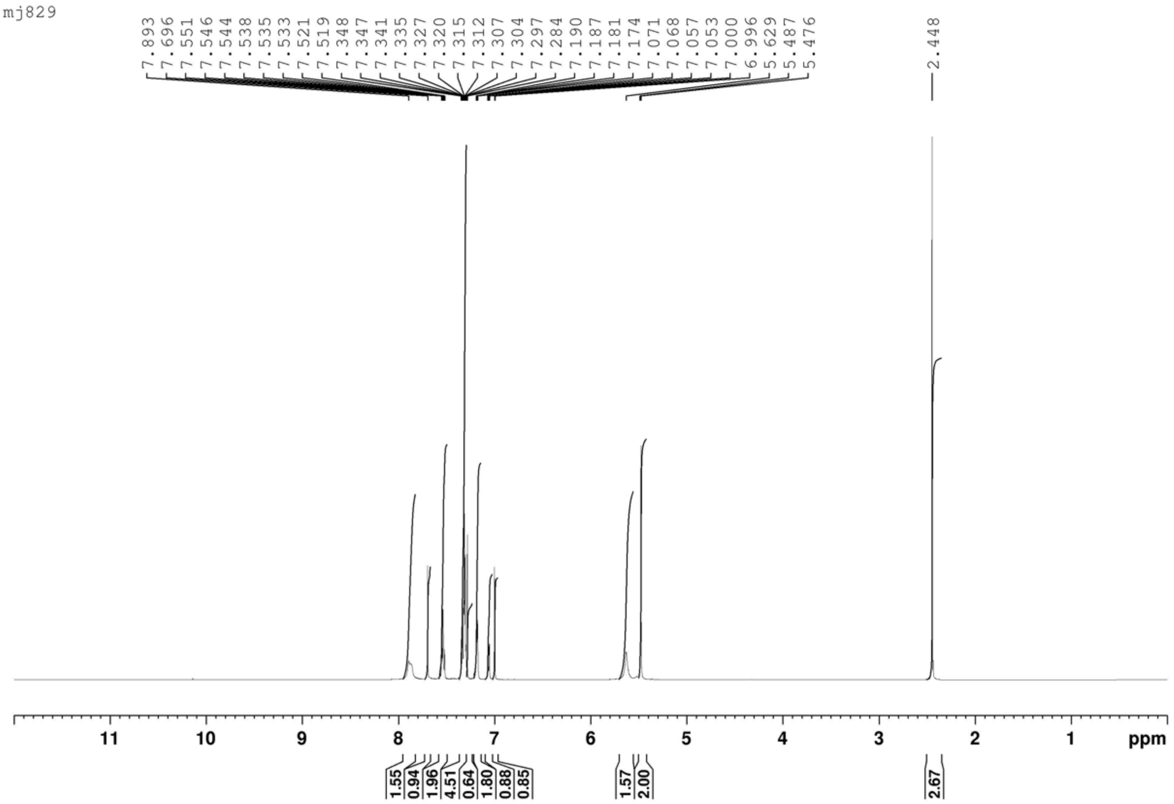
mj829
7.893
7.696
7.551
7.546
7.544
7.538
7.535
7.533
7.521
7.519
7.348
7.347
7.341
7.335
7.327
7.320
7.315
7.312
7.307
7.304
7.297
7.284
7.190
7.187
7.181
7.174
7.071
7.068
7.057
7.053
7.000
6.996
5.629
5.487
5.476
2.448
11 10 9 8 7 6 5 4 3 2 1 ppm
1.55 0.94 1.96 4.51 0.64 1.80 0.88 0.85
1.57 2.00
2.67

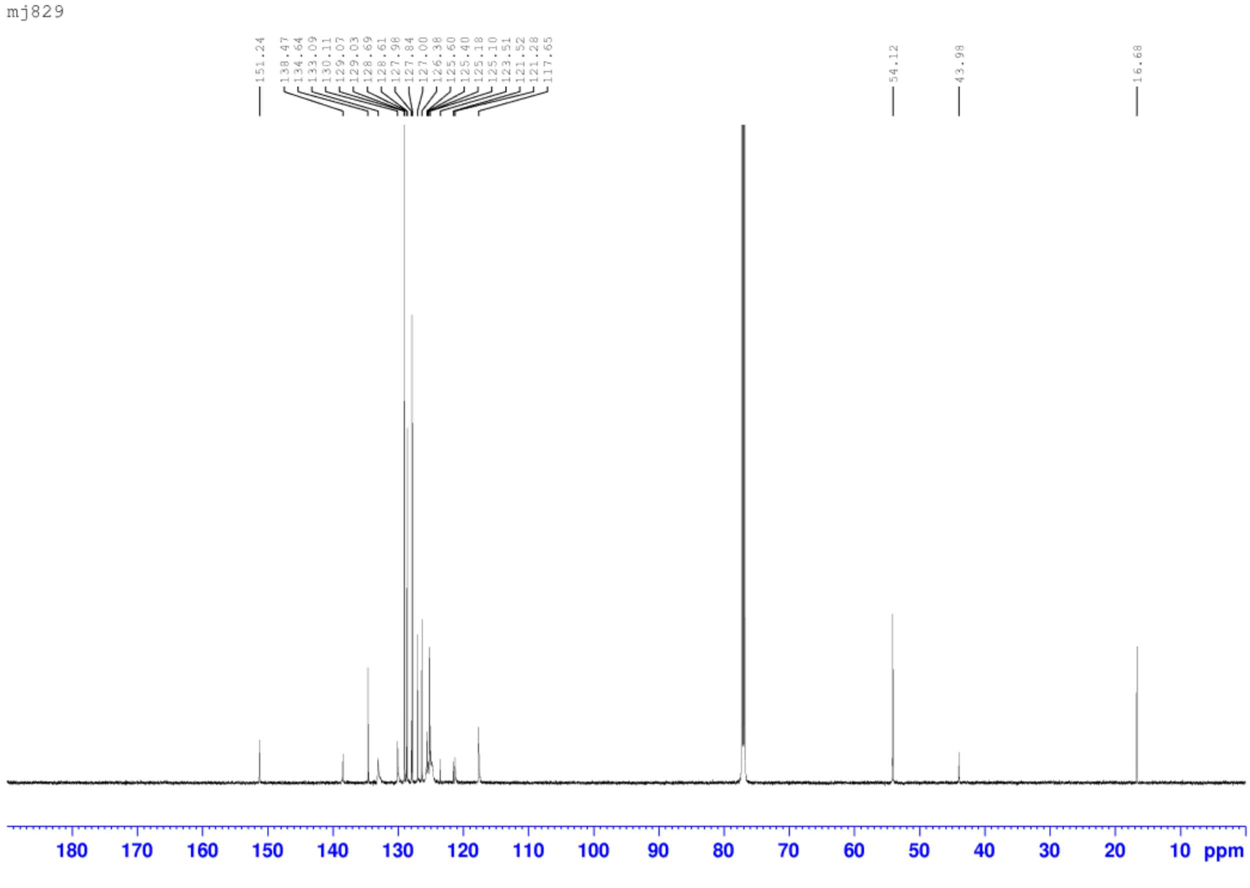
mj829
151.24
138.47
134.64
133.09
130.11
129.07
129.03
128.69
128.61
127.98
127.84
127.00
126.38
125.60
125.40
125.18
125.10
123.51
121.52
121.28
117.65
54.12
43.98
16.68
180 170 160 150 140 130 120 110 100 90 80 70 60 50 40 30 20 10 ppm

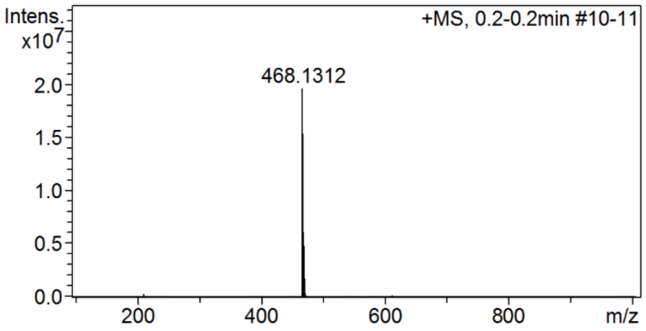

#	m/z	Res.	S/N	I	I %	FWHM
1	468.1312	34446	52679.5	19658396	100.0	0.0136

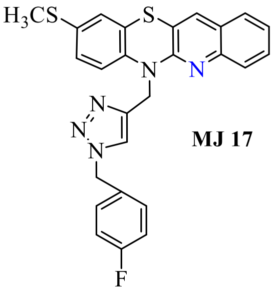

MJ 17

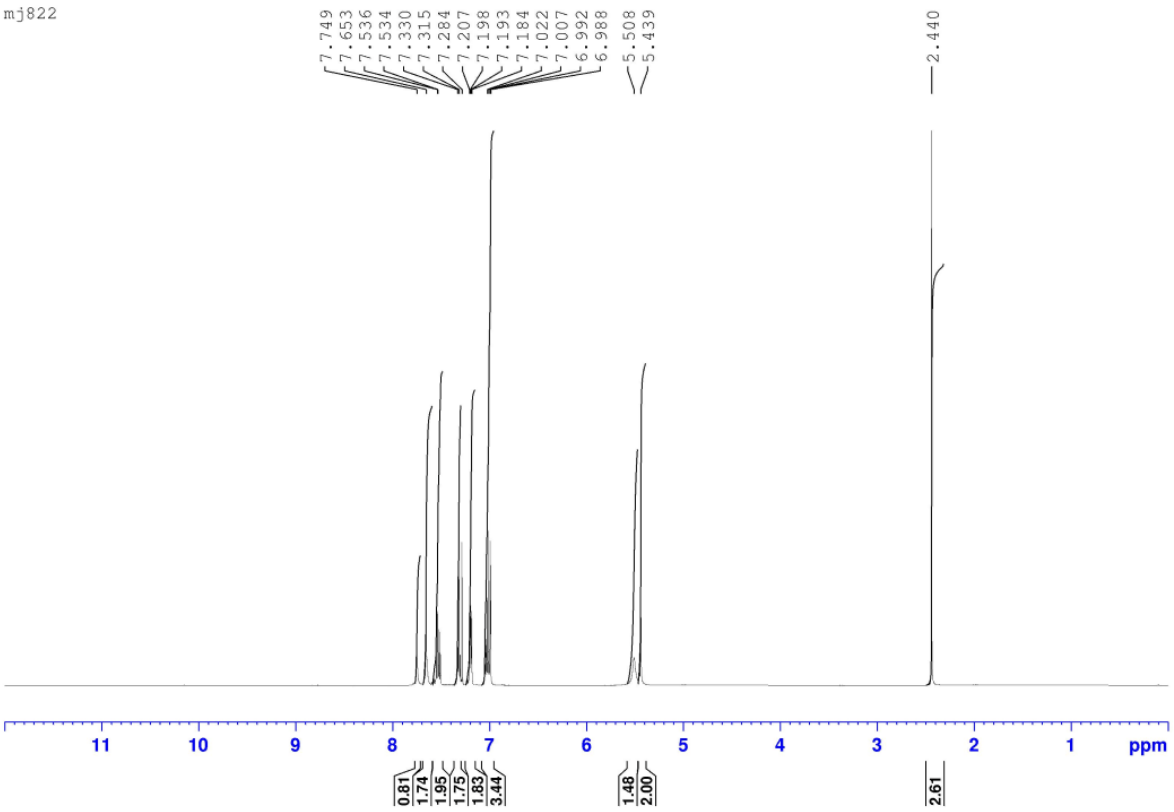

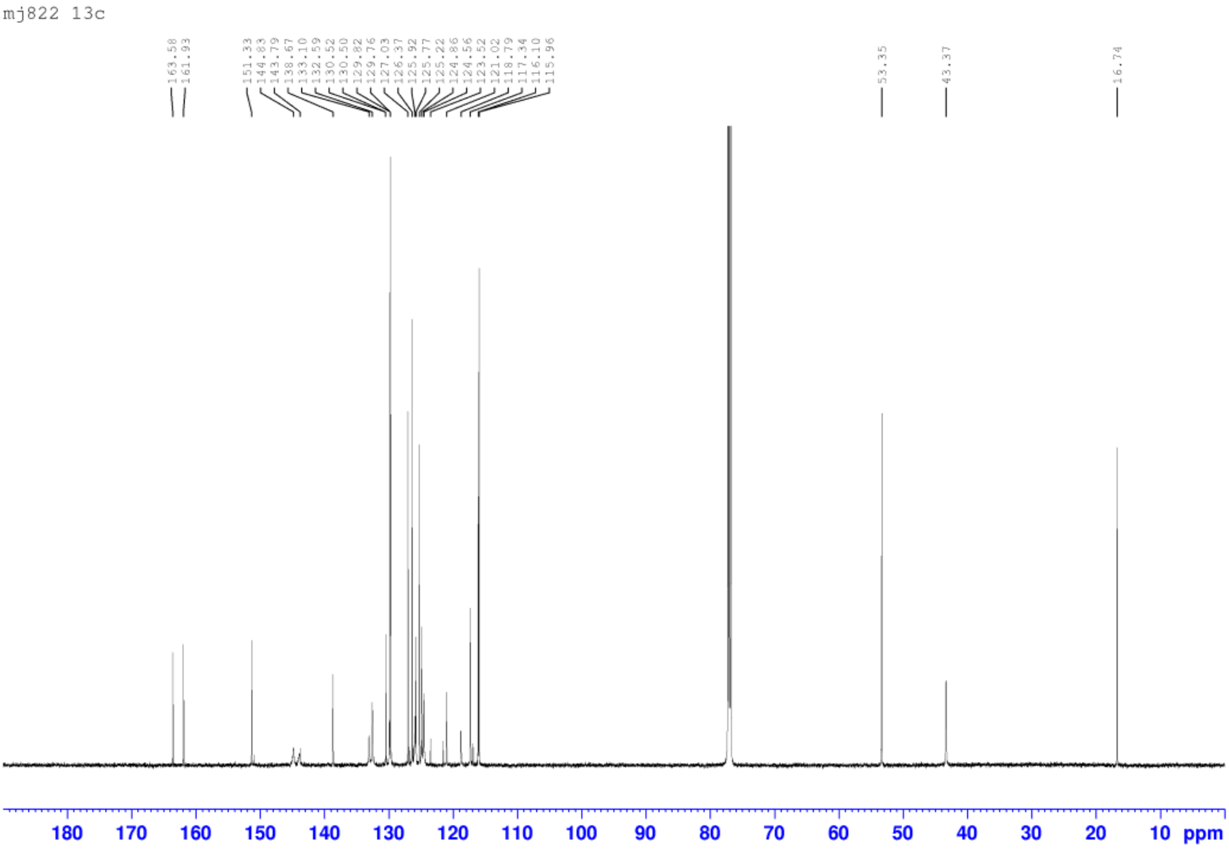

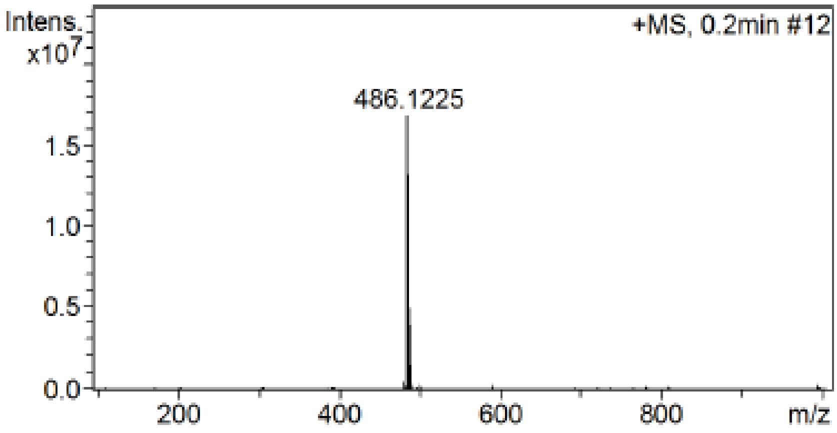

#	m/z	Res.	S/N	I	I %	FWHM
1	486.1225	45401	19213.2	16871836	100.0	0.0107
2	487.1254	44128	5625.4	4941276	29.3	0.0110

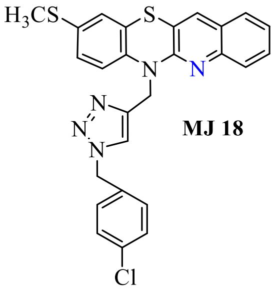

MJ 18

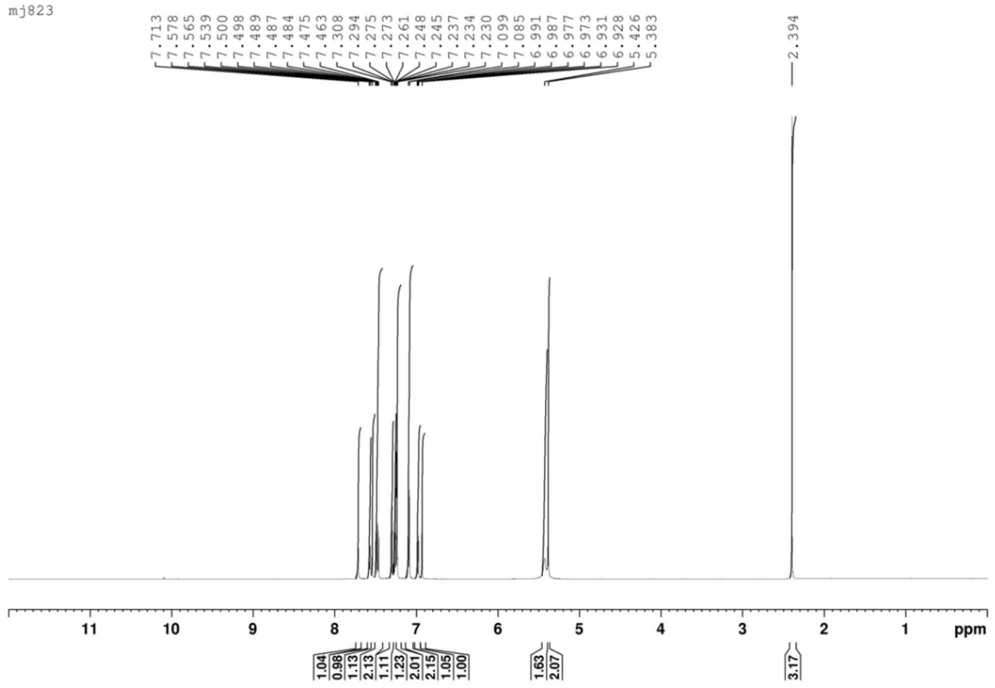
mj823
7.713
7.578
7.565
7.539
7.500
7.498
7.489
7.487
7.484
7.475
7.463
7.308
7.294
7.275
7.273
7.261
7.248
7.245
7.237
7.234
7.230
7.099
7.085
6.991
6.987
6.977
6.973
6.931
6.928
5.426
5.383
2.394
11 10 9 8 7 6 5 4 3 2 1 ppm
1.04
0.98
1.13
2.13
1.11
1.23
2.01
2.15
1.05
1.00
1.63
2.07
3.17

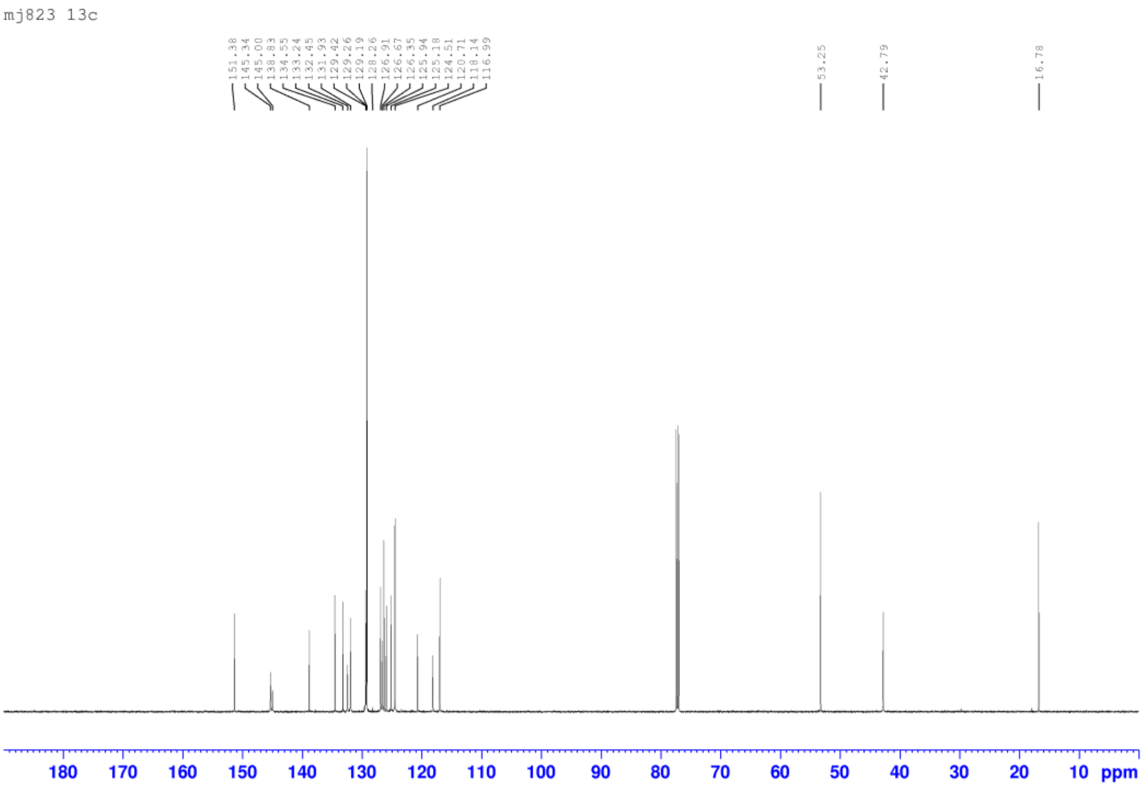

+MS, 0.1-0.2min #8-11

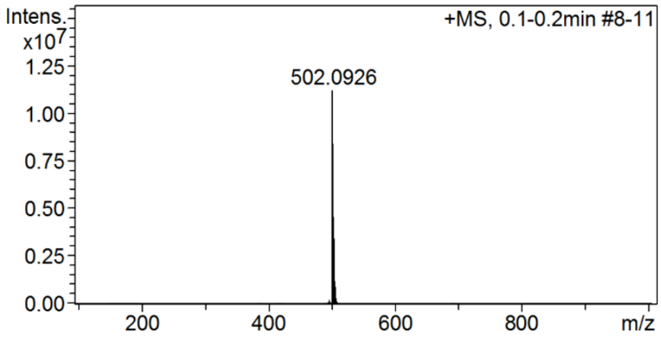

#	m/z	Res.	S/N	I	I %	FWHM
1	502.0926	46976	19702.2	11220688	100.0	0.0107
2	504.0901	42645	8037.4	4579407	40.8	0.0118

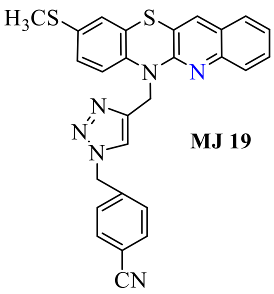

MJ 19

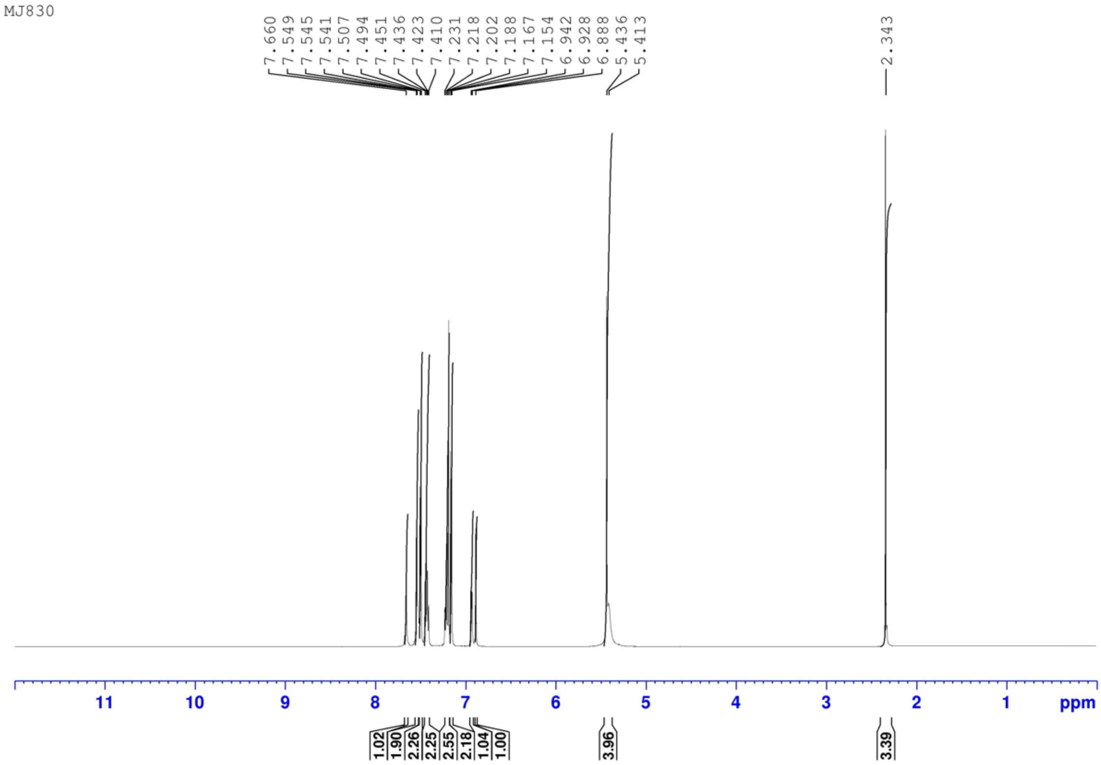
MJ830
7.660
7.549
7.545
7.541
7.507
7.494
7.451
7.436
7.423
7.410
7.231
7.218
7.202
7.188
7.167
7.154
6.942
6.928
6.888
5.436
5.413
2.343
11 10 9 8 7 6 5 4 3 2 1 ppm
1.02
1.90
2.26
2.25
2.55
2.18
1.04
1.00
3.96
3.39

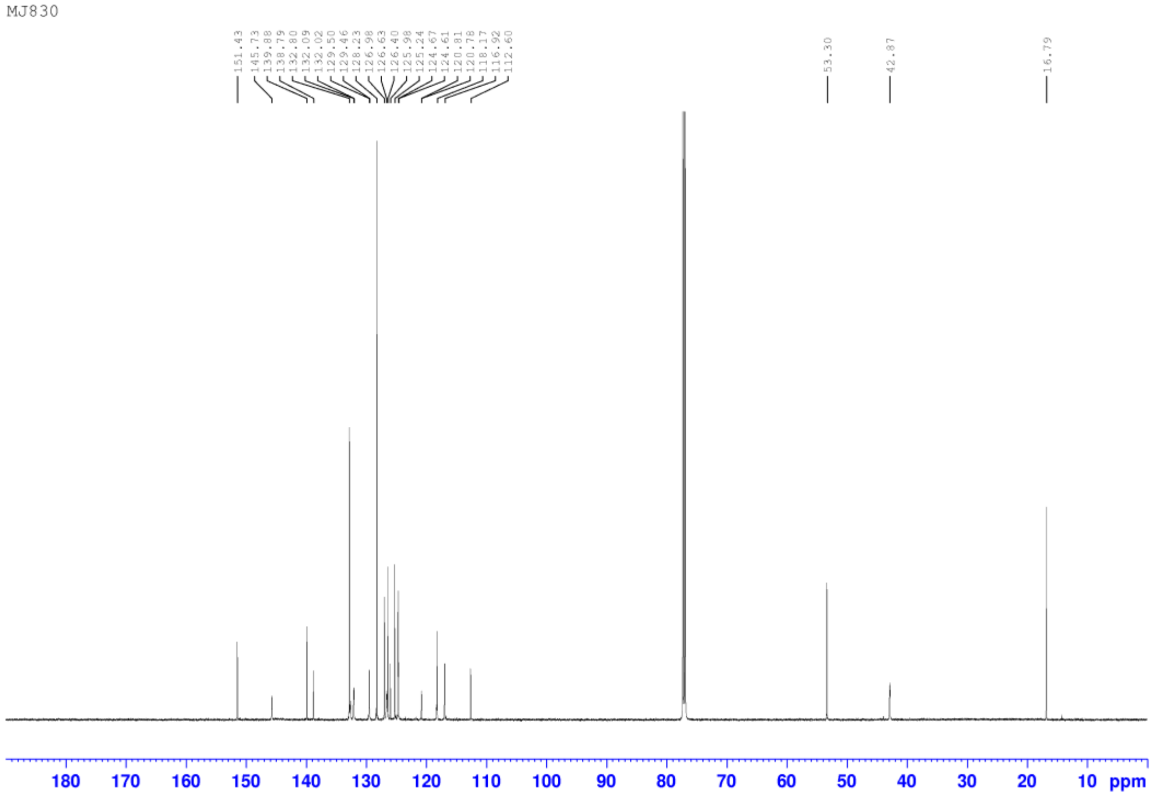
MJ830
151.43
145.73
139.88
138.79
132.80
132.09
132.02
129.50
129.46
128.23
126.98
126.63
126.40
125.98
125.24
124.67
124.61
120.81
120.78
118.17
116.92
112.60
53.30
42.87
16.79
180 170 160 150 140 130 120 110 100 90 80 70 60 50 40 30 20 10 ppm

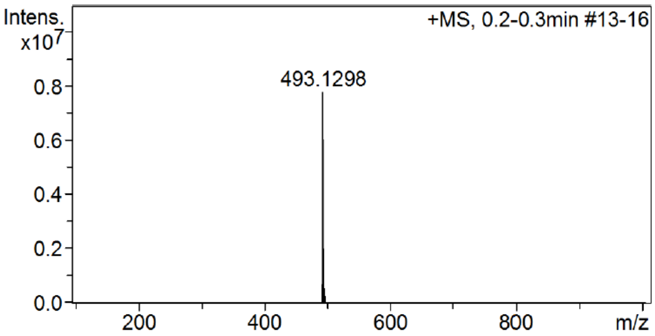

#	m/z	Res.	S/N	I	I %	FWHM
1	493.1298	46079	16943.8	7799905	100.0	0.0107

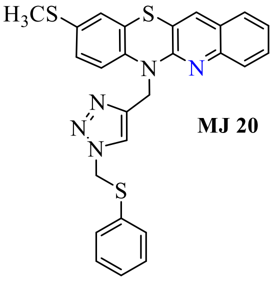

MJ 20

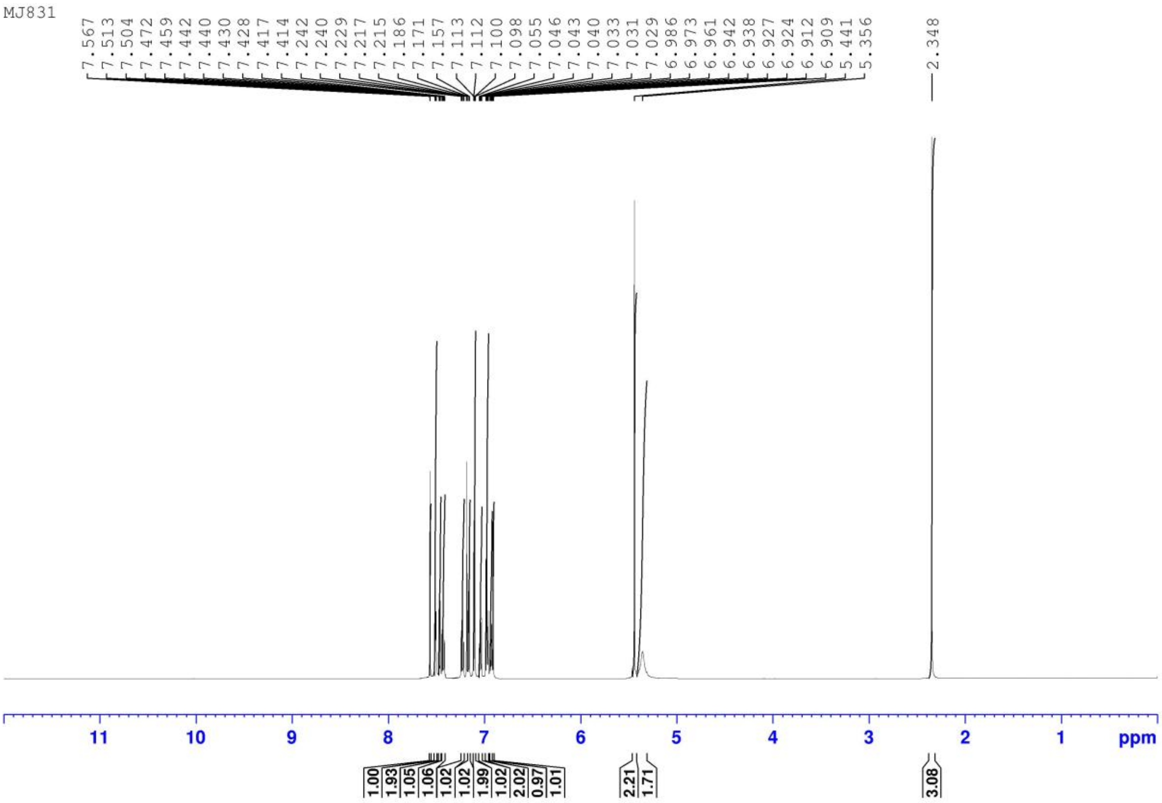

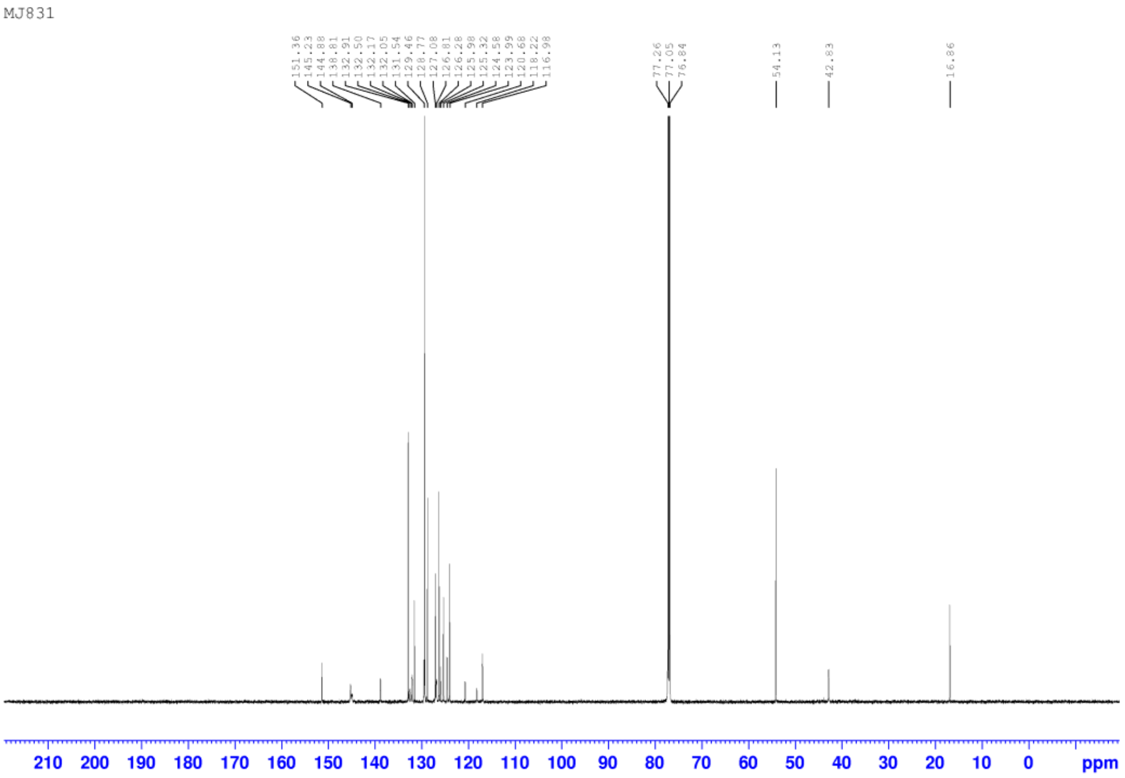

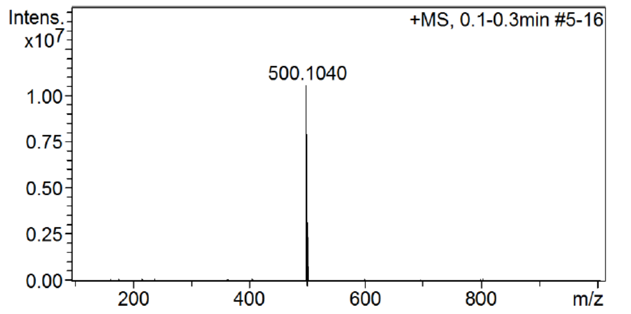

#	m/z	Res.	S/N	I	I %	FWHM
1	500.1040	46524	11010.2	10576527	100.0	0.0107
