# Supplementary material for: Design, Synthesis, and Testing of 1,2,3-Triazolo-Quinobenzothiazine Hybrids for Cytotoxic and Immunomodulatory Activity
Source: Int J Mol Sci. 2025 Jul 18;26(14):6920. doi: 10.3390/ijms26146920 (PMC12295927; doi:10.3390/ijms26146920)

Design, synthesis, and cytotoxic properties of 1,2,3-triazolo-azaphenothiazines hybrids.

Klaudia Giercuszkiewicz-Haśnik [1,2], Magdalena Skonieczna [1,2], Beata Morak-Młodawska [3], Małgorzata Jeleń [3*]

[1] Department of Systems Biology and Engineering, The Silesian University of Technology, Akademicka Street 16, 44–100 Gliwice, Poland; magdalena.skonieczna@polsl.pl (M.S.)

[2] Centre of Biotechnology, Silesian University of Technology, Krzywoustego Street 8, 44-100 Gliwice, Poland

[3] Department of Organic Chemistry, Faculty of Pharmaceutical Sciences in Sosnowiec, Medical University of Silesia in Katowice, Jagiellońska Street 4, 41-200 Sosnowiec, Poland; bmlodawska@sum.edu.pl (B.M.-M.)

Content:

Images of BEAS-2B and HCT116 cells after 24 h incubation with **MJ1-MJ20** compounds at concentrations of 6.25-100 μM, and images of NHDF, MCF7, SH-SY5Y, and A549 cells lines after 24 h incubation with **MJ1-MJ20** compounds at concentrations of 100 μM.

Images of BEAS-2B and HCT116 cell line nuclei after 24h of incubation with **MJ2**, **MJ8**, **MJ15**, **MJ19** compounds at concentration of 100μM after DAPI staining.

1. BEAS-2B MJ1-MJ20 (6.25-100μM)

Control BEAS-2B

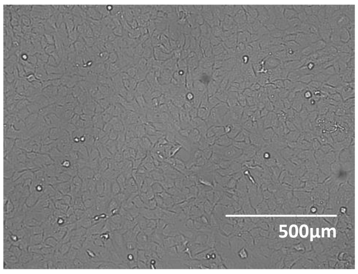

MJ1 6,25μM BEAS-2B

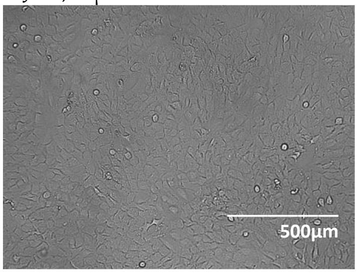

MJ1 12,5μM BEAS-2B

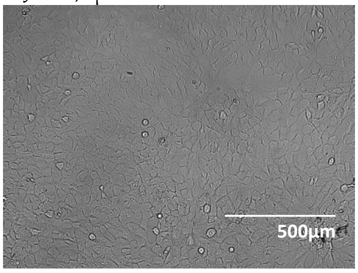

MJ1 25μM BEAS-2B

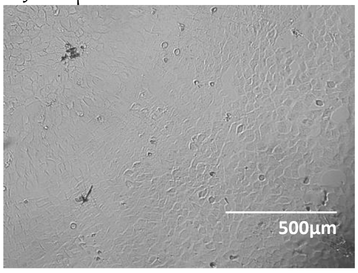

MJ1 50μM BEAS-2B

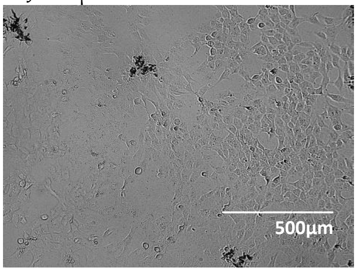

MJ1 100μM BEAS-2B

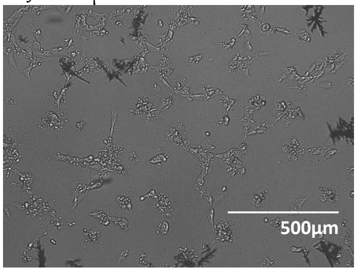

Control BEAS-2B

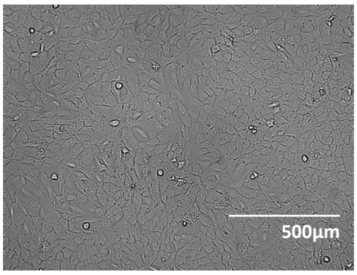

MJ2 6,25μM BEAS-2B

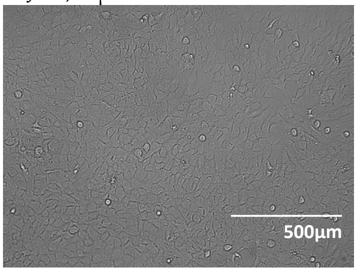

MJ2 12,5μM BEAS-2B

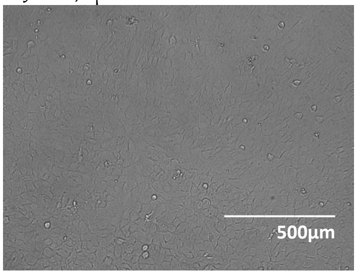

MJ2 25μM BEAS-2B

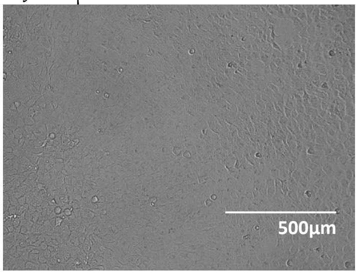

MJ2 50μM BEAS-2B

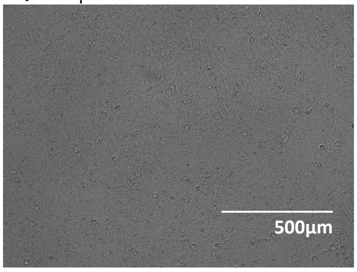

MJ2 100μM BEAS-2B

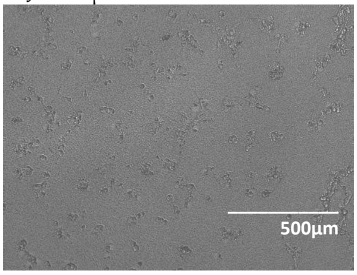

Control BEAS-2B

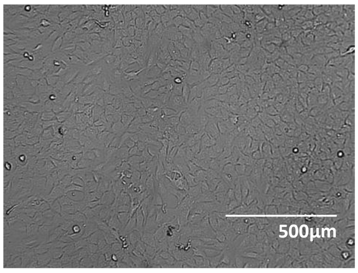

MJ3 6,25µM BEAS-2B

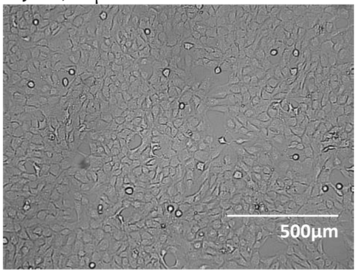

MJ3 12,5µM BEAS-2B

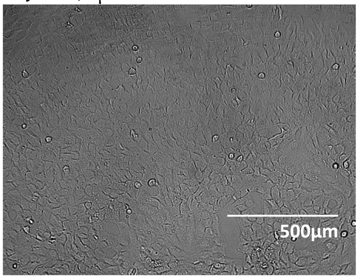

MJ3 25µM BEAS-2B

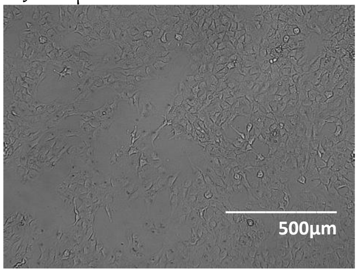

MJ3 50µM BEAS-2B

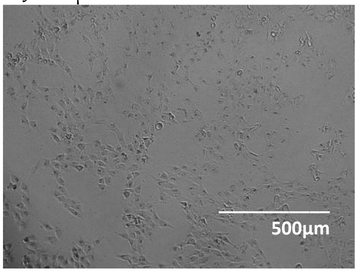

MJ3 100µM BEAS-2B

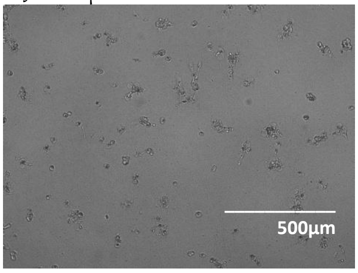

Control BEAS-2B

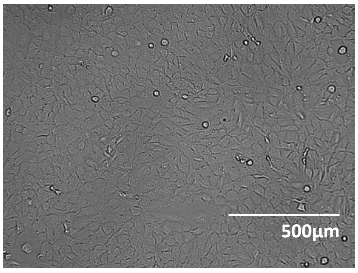

MJ4 6,25µM BEAS-2B

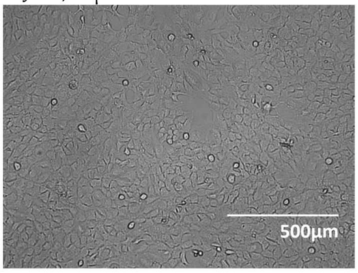

MJ4 12,5µM BEAS-2B

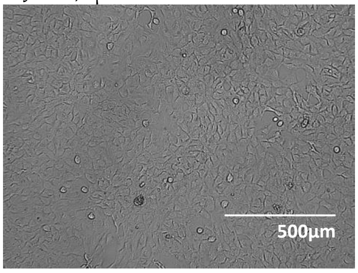

MJ4 25µM BEAS-2B

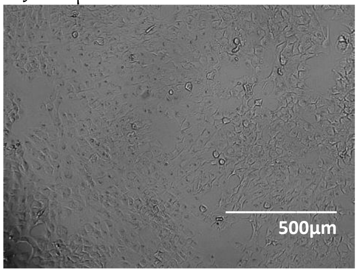

MJ4 50µM BEAS-2B

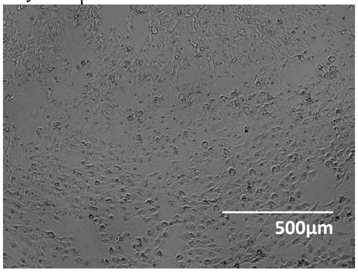

MJ4 100µM BEAS-2B

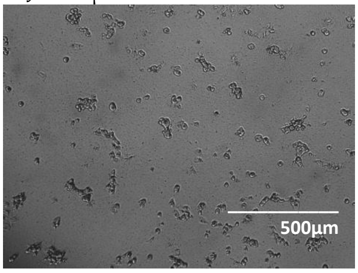

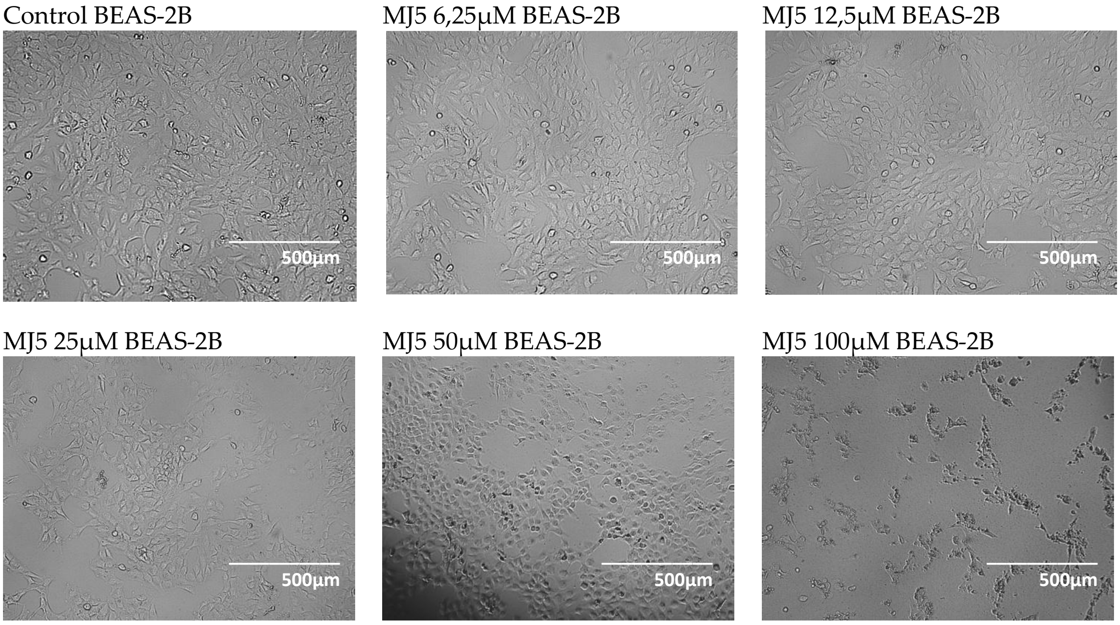
Control BEAS-2B
MJ5 6,25µM BEAS-2B
MJ5 12,5µM BEAS-2B
MJ5 25µM BEAS-2B
MJ5 50µM BEAS-2B
MJ5 100µM BEAS-2B
500µm

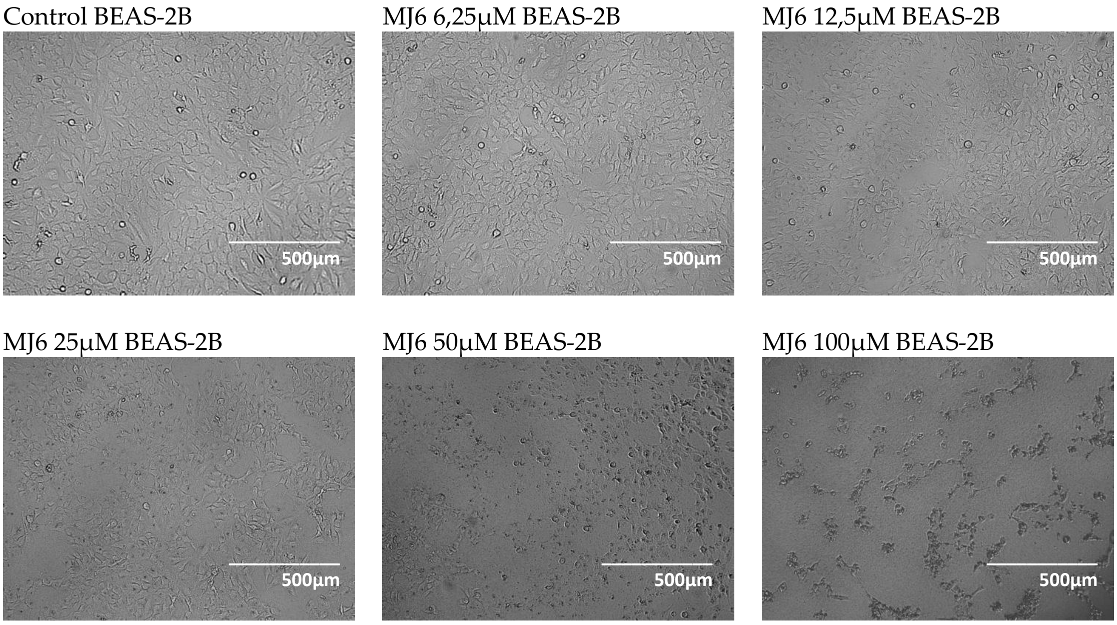
Control BEAS-2B
MJ6 6,25µM BEAS-2B
MJ6 12,5µM BEAS-2B
MJ6 25µM BEAS-2B
MJ6 50µM BEAS-2B
MJ6 100µM BEAS-2B
500µm

Control BEAS-2B

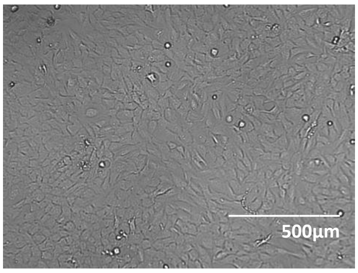

MJ7 6,25μM BEAS-2B

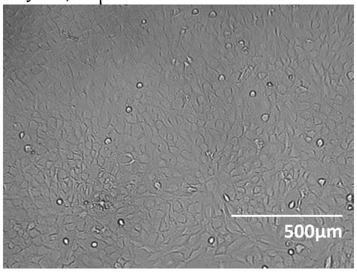

MJ7 12,5μM BEAS-2B

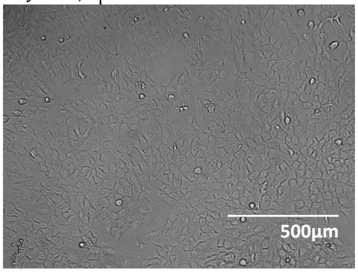

MJ7 25μM BEAS-2B

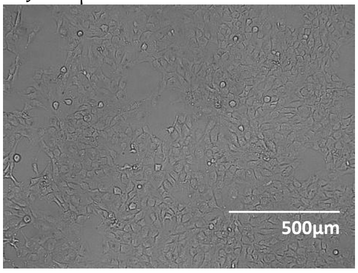

MJ7 50μM BEAS-2B

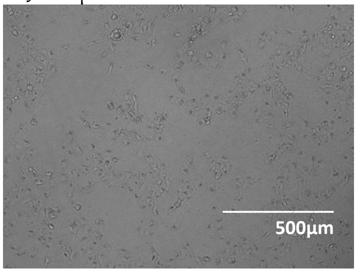

MJ7 100μM BEAS-2B

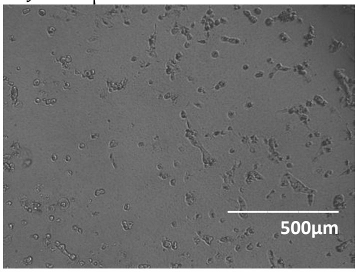

Control BEAS-2B

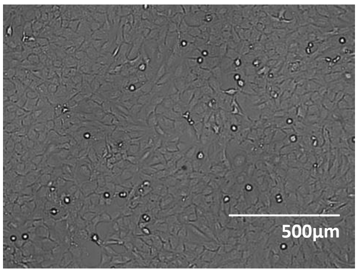

MJ8 6,25μM BEAS-2B

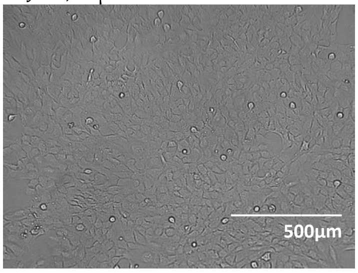

MJ8 12,5μM BEAS-2B

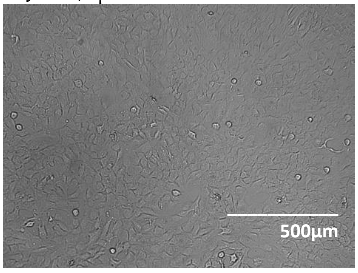

MJ8 25μM BEAS-2B

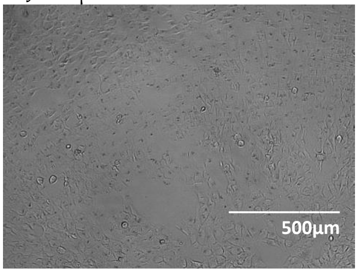

MJ8 50μM BEAS-2B

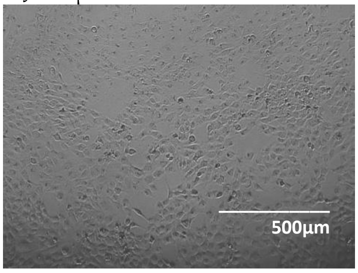

MJ8 100μM BEAS-2B

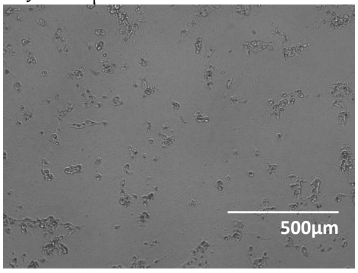

Control BEAS-2B

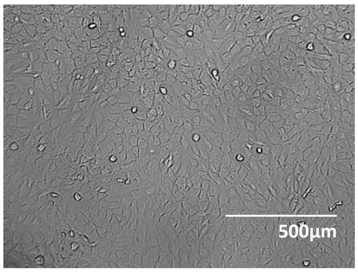

MJ9 6,25µM BEAS-2B

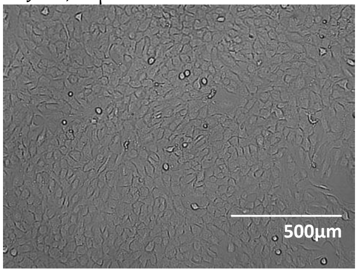

MJ9 12,5µM BEAS-2B

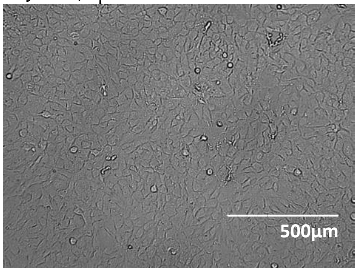

MJ9 25µM BEAS-2B

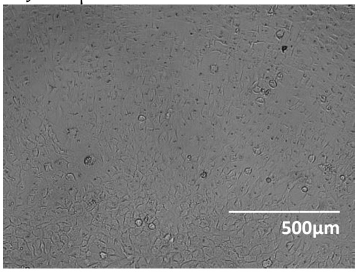

MJ9 50µM BEAS-2B

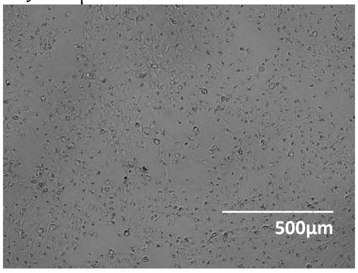

MJ9 100µM BEAS-2B

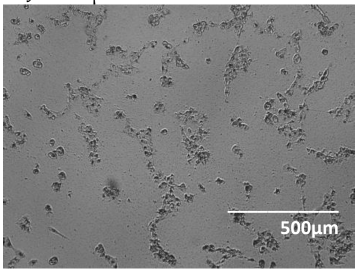

Control BEAS-2B

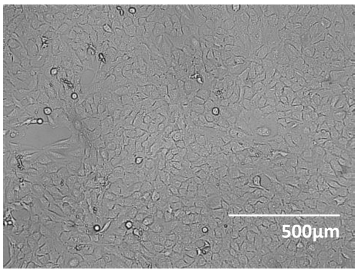

MJ10 6,25µM BEAS-2B

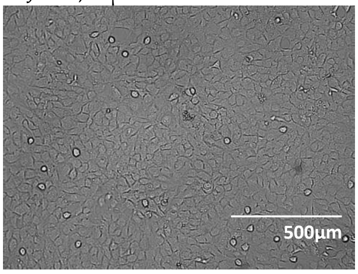

MJ10 12,5µM BEAS-2B

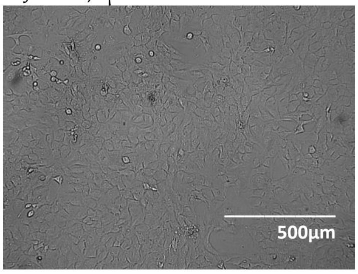

MJ10 25µM BEAS-2B

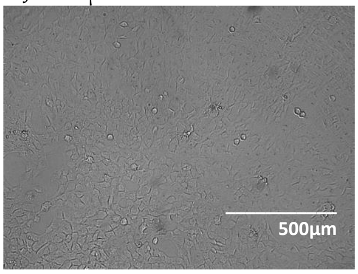

MJ10 50µM BEAS-2B

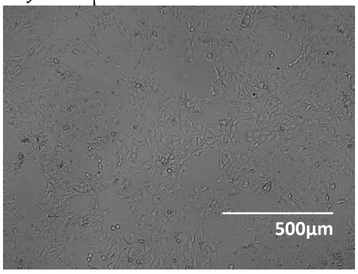

MJ10 100µM BEAS-2B

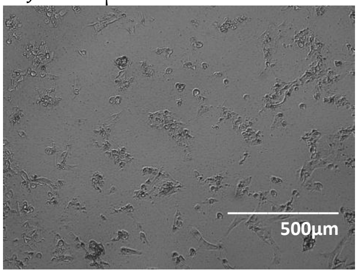

Control BEAS-2B

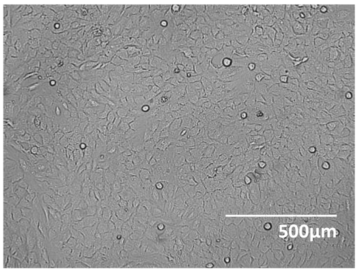

MJ11 6,25μM BEAS-2B

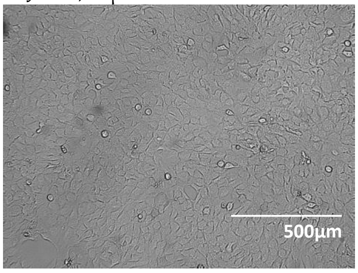

MJ11 12,5μM BEAS-2B

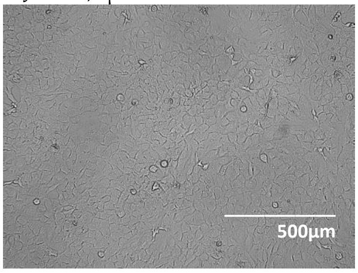

MJ11 25μM BEAS-2B

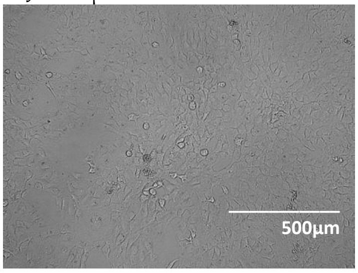

MJ11 50μM BEAS-2B

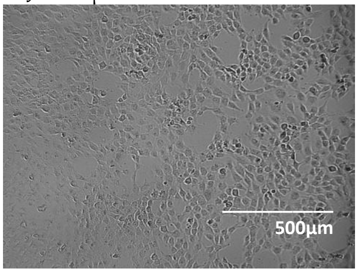

MJ11 100μM BEAS-2B

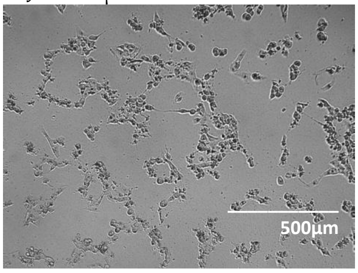

Control BEAS-2B

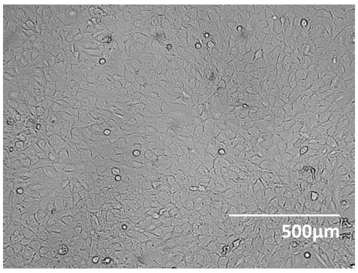

MJ12 6,25μM BEAS-2B

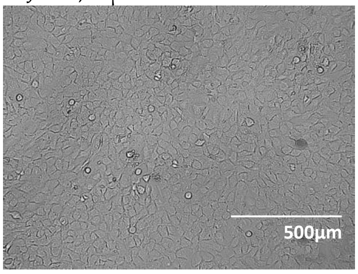

MJ12 12,5μM BEAS-2B

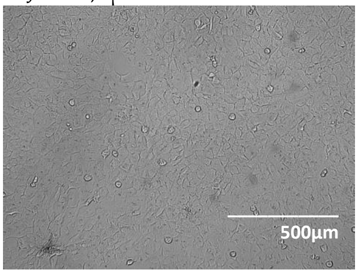

MJ12 25μM BEAS-2B

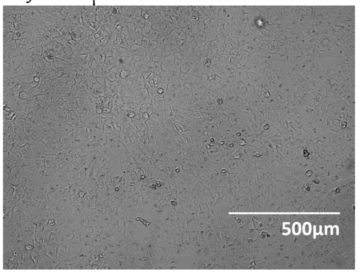

MJ12 50μM BEAS-2B

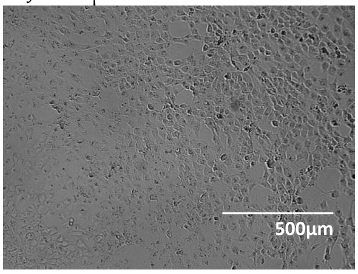

MJ12 100μM BEAS-2B

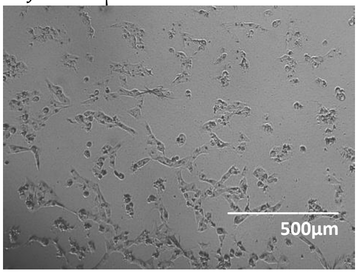

Control BEAS-2B

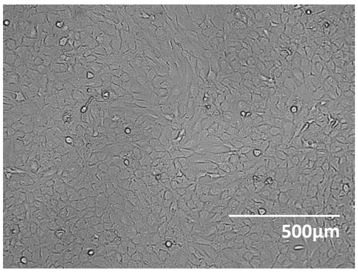

MJ13 6,25µM BEAS-2B

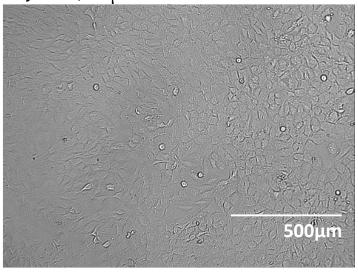

MJ13 12,5µM BEAS-2B

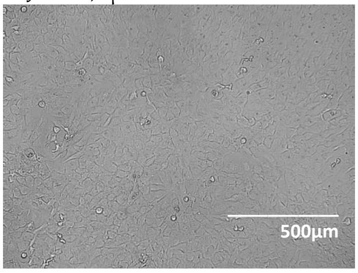

MJ13 25µM BEAS-2B

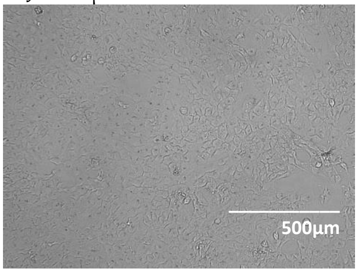

MJ13 50µM BEAS-2B

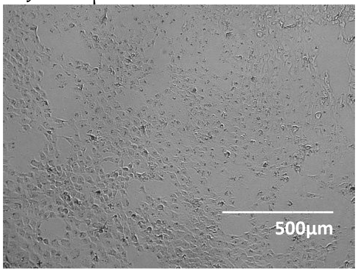

MJ13 100µM BEAS-2B

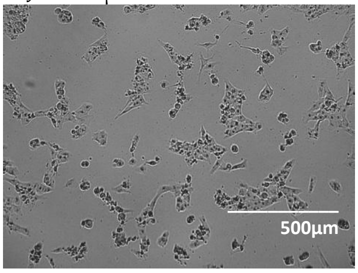

Control BEAS-2B

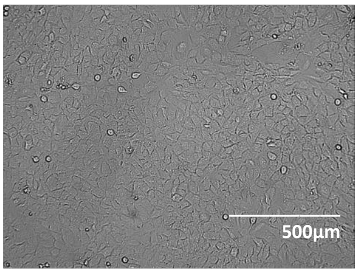

MJ14 6,25µM BEAS-2B

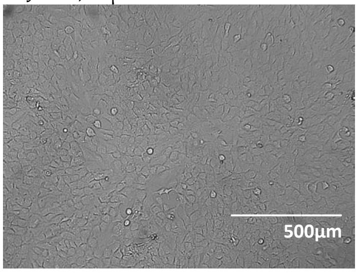

MJ14 12,5µM BEAS-2B

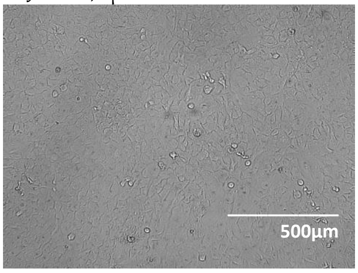

MJ14 25µM BEAS-2B

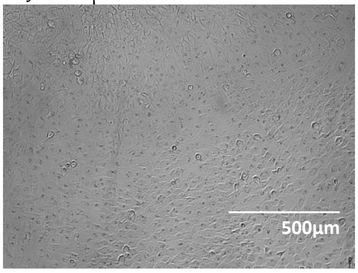

MJ14 50µM BEAS-2B

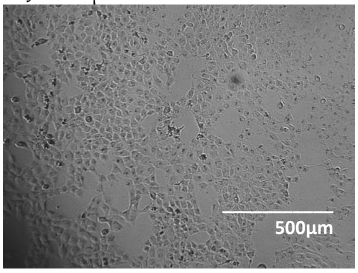

MJ14 100µM BEAS-2B

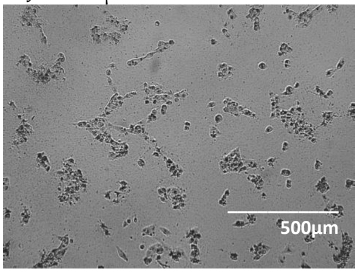

Control BEAS-2B

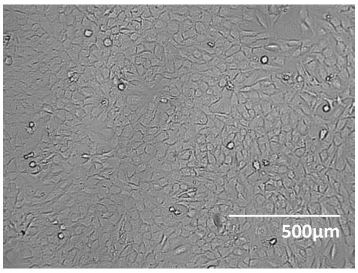

MJ15 6,25µM BEAS-2B

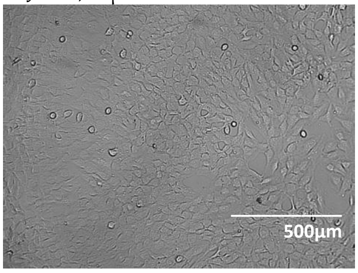

MJ15 12,5µM BEAS-2B

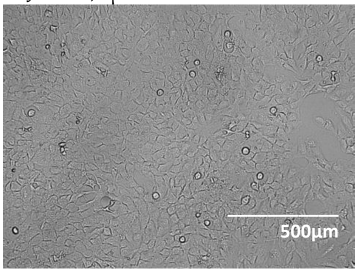

MJ15 25µM BEAS-2B

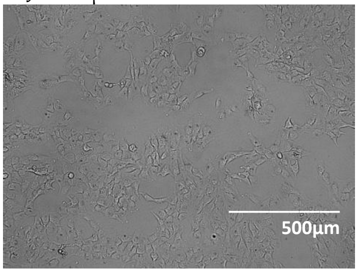

MJ15 50µM BEAS-2B

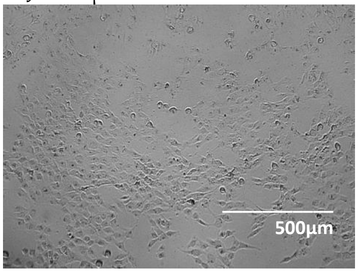

MJ15 100µM BEAS-2B

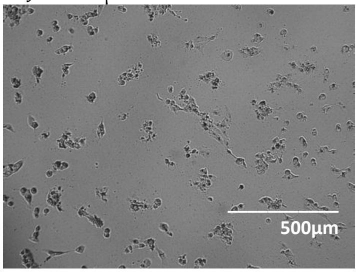

Control BEAS-2B

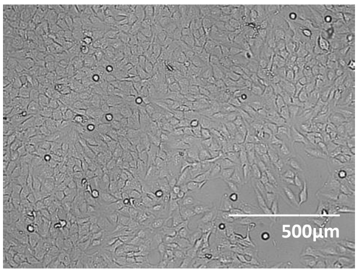

MJ16 6,25µM BEAS-2B

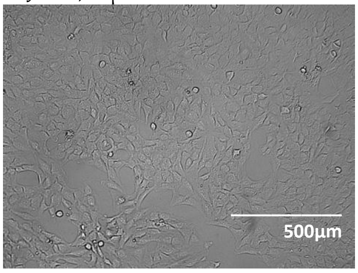

MJ16 12,5µM BEAS-2B

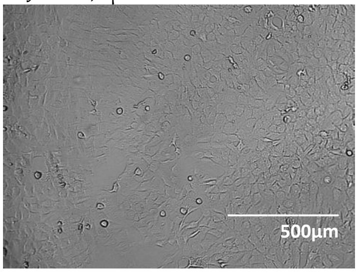

MJ16 25µM BEAS-2B

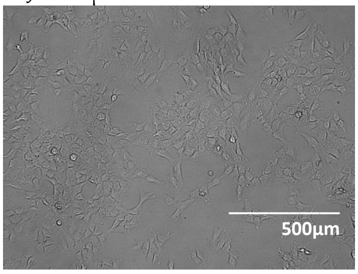

MJ16 50µM BEAS-2B

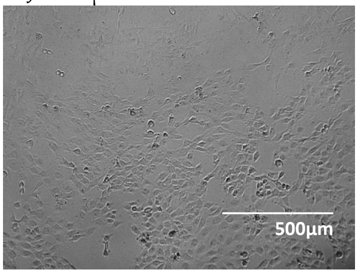

MJ16 100µM BEAS-2B

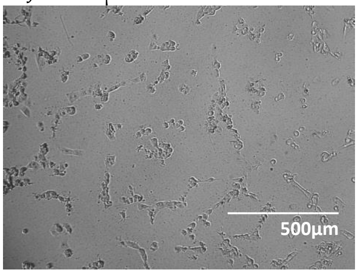

Control BEAS-2B

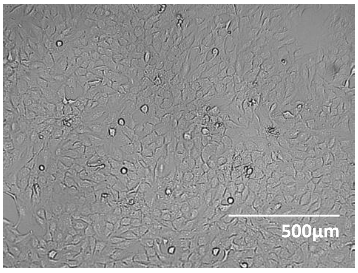

MJ17 6,25µM BEAS-2B

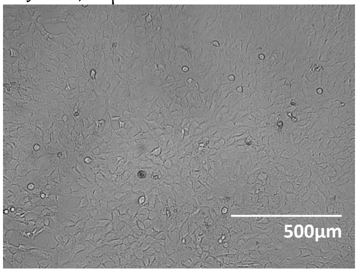

MJ17 12,5µM BEAS-2B

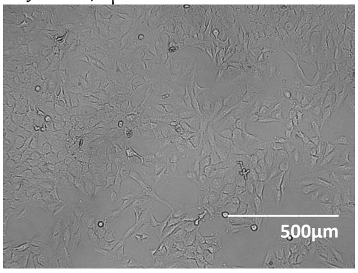

MJ17 25µM BEAS-2B

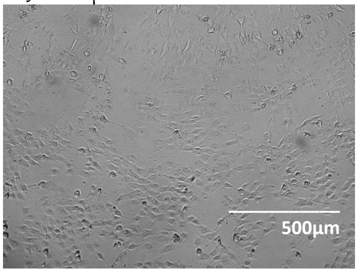

MJ17 50µM BEAS-2B

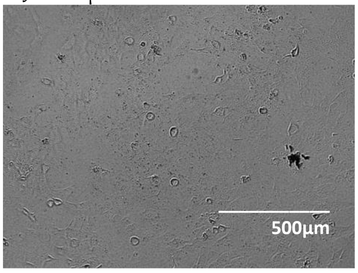

MJ17 100µM BEAS-2B

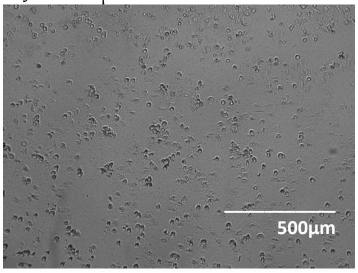

Control BEAS-2B

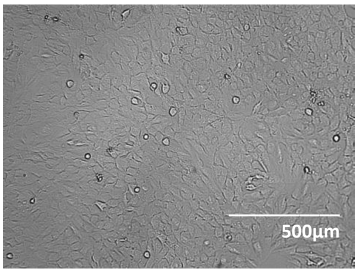

MJ18 6,25µM BEAS-2B

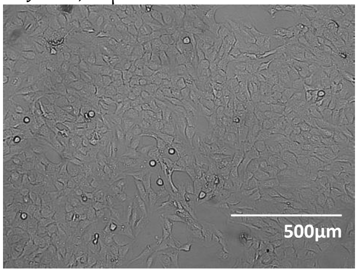

MJ18 12,5µM BEAS-2B

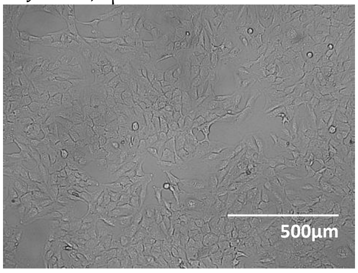

MJ18 25µM BEAS-2B

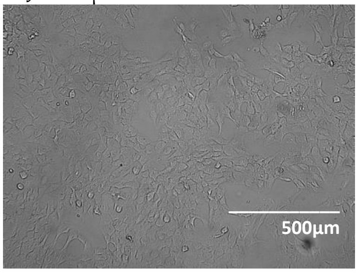

MJ18 50µM BEAS-2B

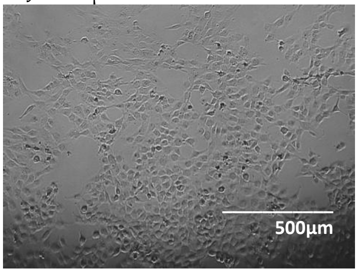

MJ18 100µM BEAS-2B

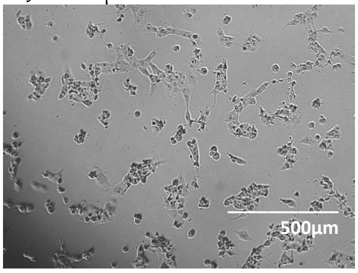

Control BEAS-2B

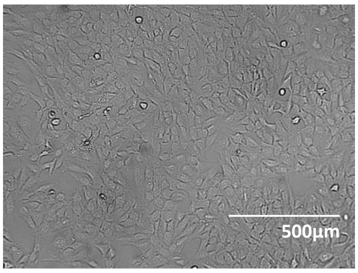

MJ19 6,25µM BEAS-2B

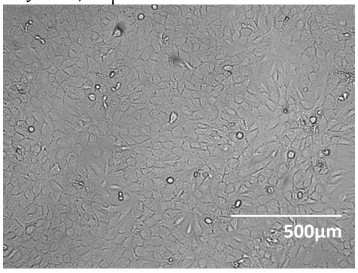

MJ19 12,5µM BEAS-2B

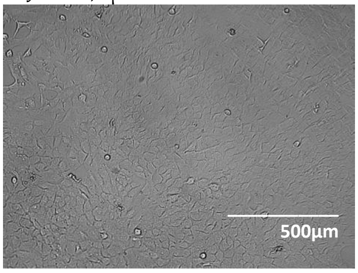

MJ19 25µM BEAS-2B

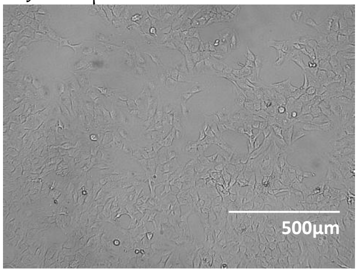

MJ19 50µM BEAS-2B

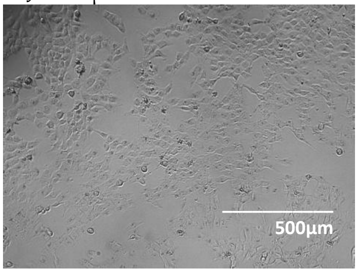

MJ19 100µM BEAS-2B

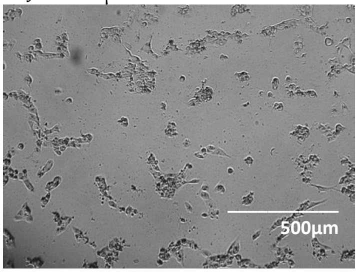

Control BEAS-2B

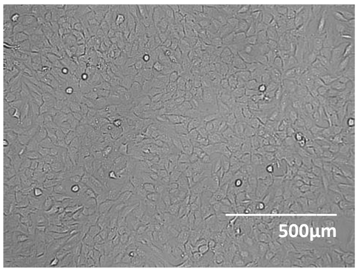

MJ20 6,25µM BEAS-2B

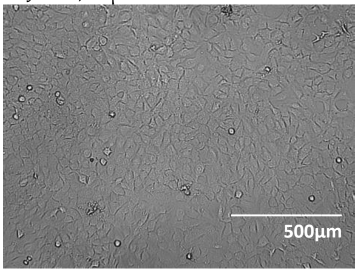

MJ20 12,5µM BEAS-2B

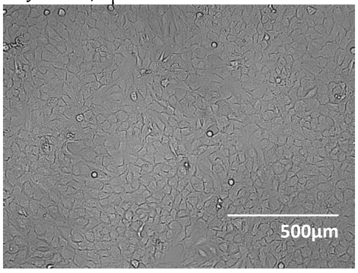

MJ20 25µM BEAS-2B

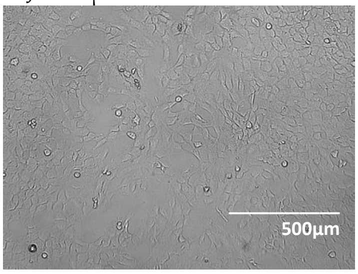

MJ20 50µM BEAS-2B

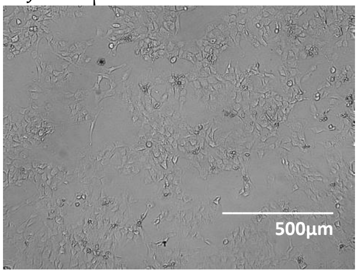

MJ20 100µM BEAS-2B

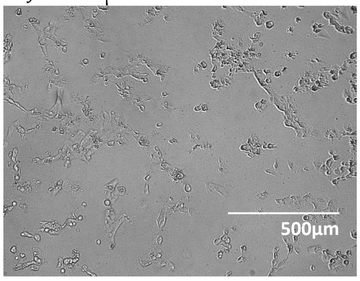

2. BEAS-2B MJ1-MJ20 (100μM)

Control BEAS-2B

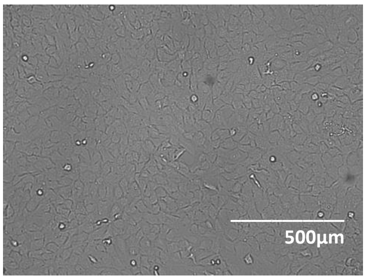

MJ1 100μM BEAS-2B

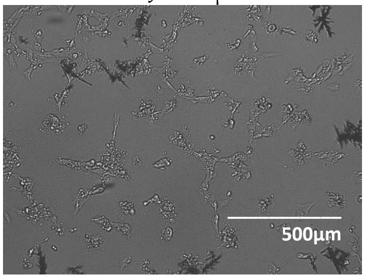

MJ2 100μM BEAS-2B

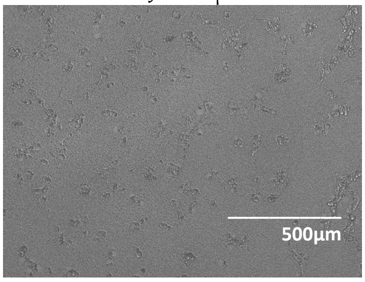

MJ3 100μM BEAS-2B

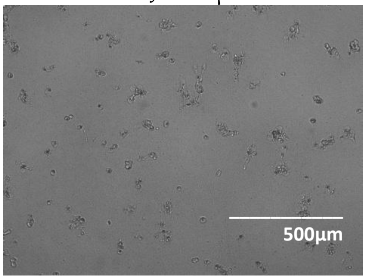

MJ4 100μM BEAS-2B

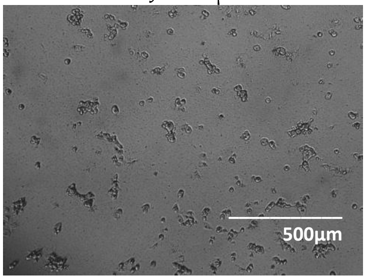

MJ5 100μM BEAS-2B

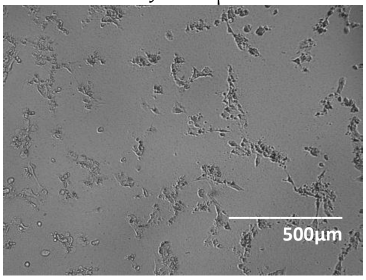

MJ6 100μM BEAS-2B

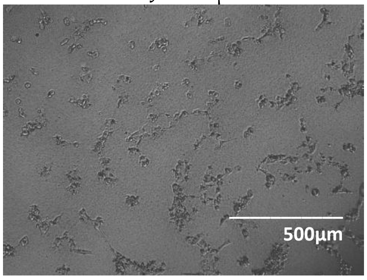

MJ7 100μM BEAS-2B

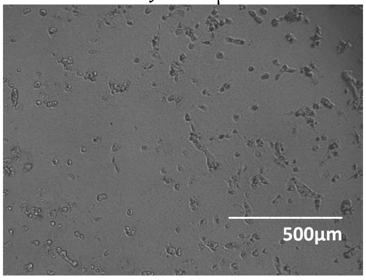

MJ8 100μM BEAS-2B

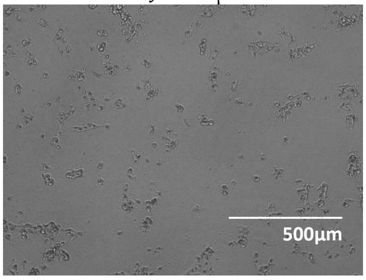

MJ9 100μM BEAS-2B

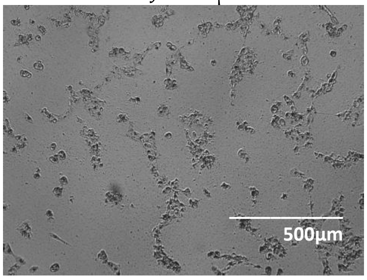

MJ10 100μM BEAS-2B

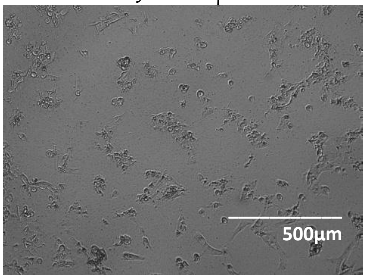

MJ11 100μM BEAS-2B

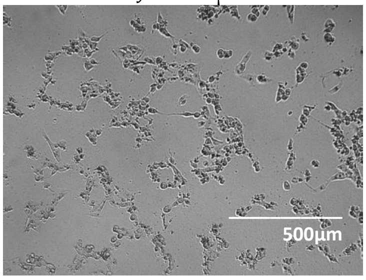

MJ12 100μM BEAS-2B

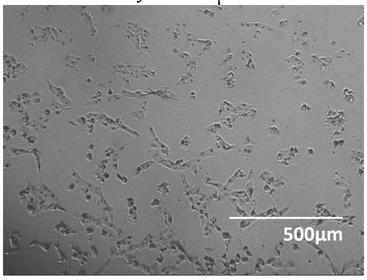

MJ13 100μM BEAS-2B

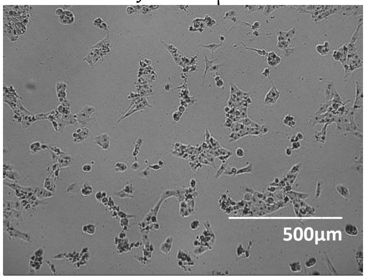

MJ14 100μM BEAS-2B

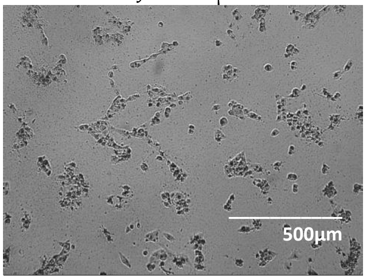

MJ15 100µM BEAS-2B

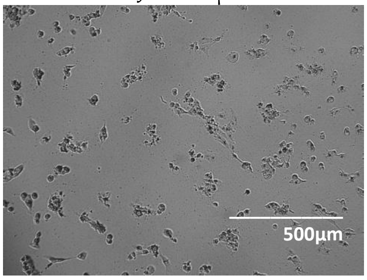

MJ16 100µM BEAS-2B

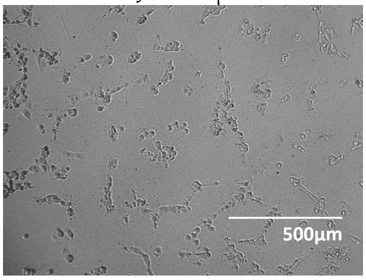

MJ17 100µM BEAS-2B

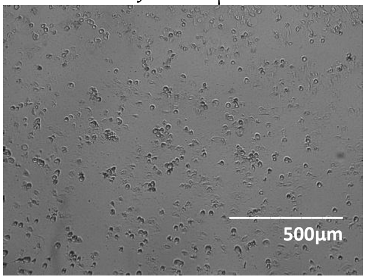

MJ18 100µM BEAS-2B

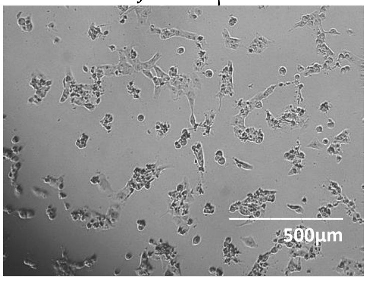

MJ19 100µM BEAS-2B

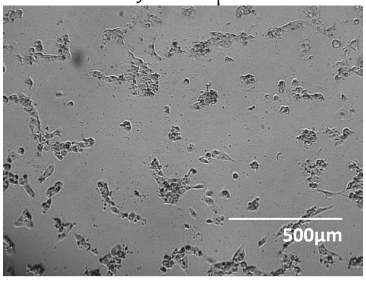

MJ20 100µM BEAS-2B

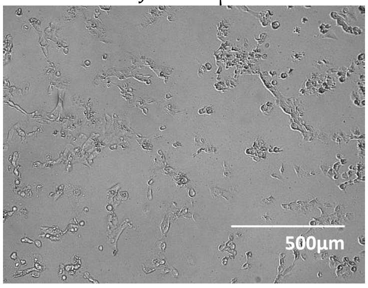

3. HCT116 MJ1-MJ20 (6.25-100µM)

Control HCT116

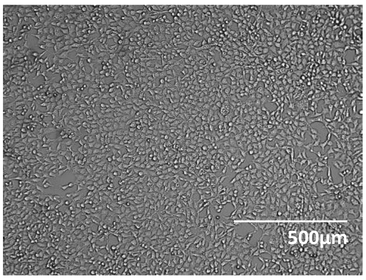

MJ1 6,25µM HCT116

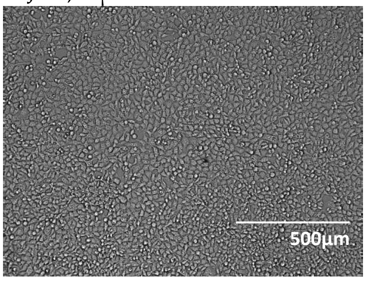

MJ1 12,5µM HCT116

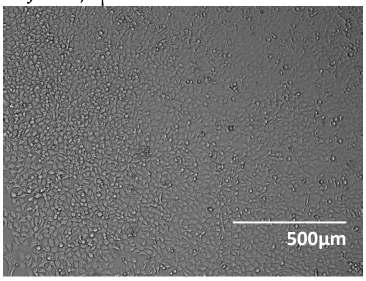

MJ1 25µM HCT116

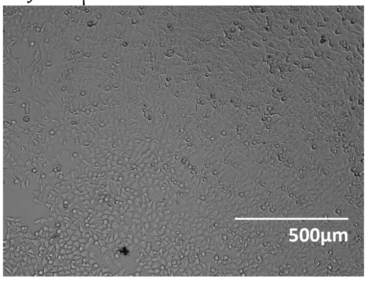

MJ1 50µM HCT116

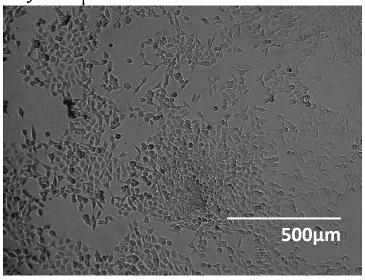

MJ1 100µM HCT116

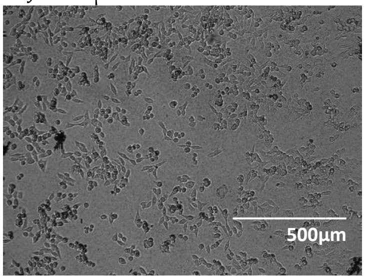

Control HCT116

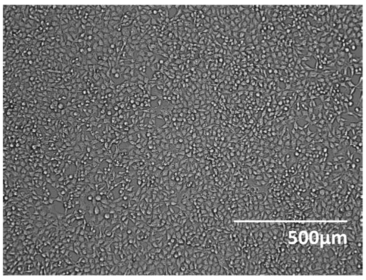

MJ2 6,25µM HCT116

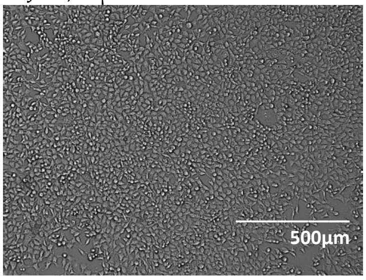

MJ2 12,5µM HCT116

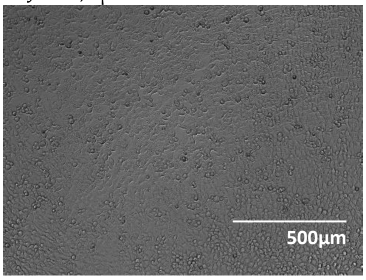

MJ2 25µM HCT116

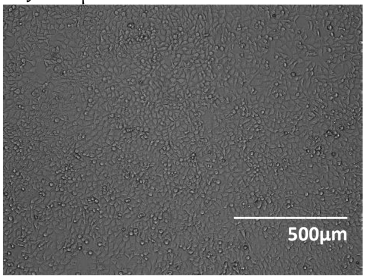

MJ2 50µM HCT116

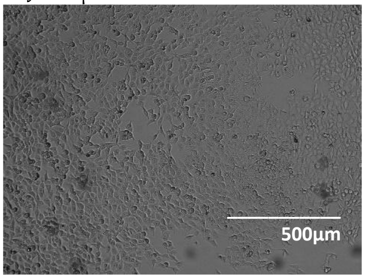

MJ2 100µM HCT116

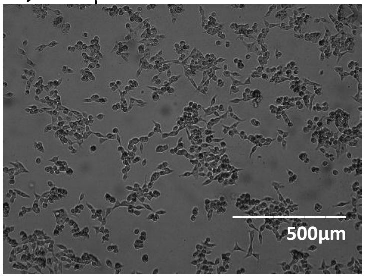

Control HCT116

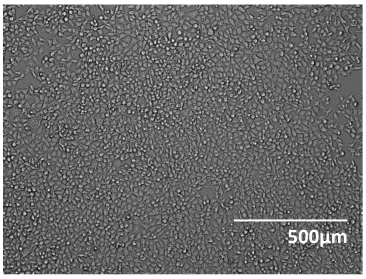

MJ4 6,25µM HCT116

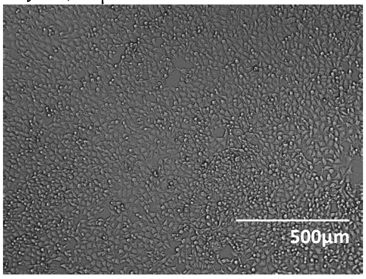

MJ4 12,5µM HCT116

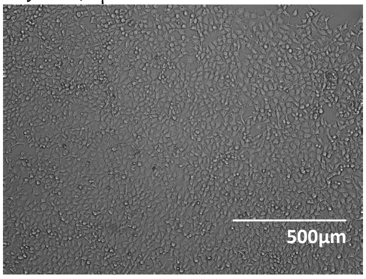

MJ4 25µM HCT116

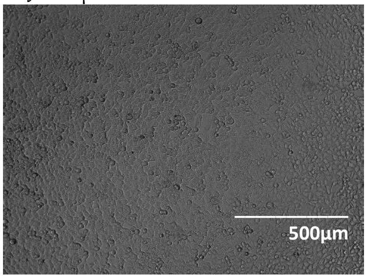

MJ4 50µM HCT116

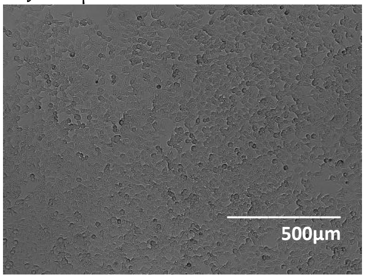

MJ4 100µM HCT116

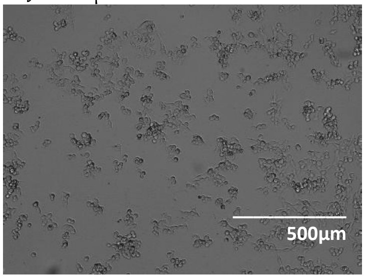

Control HCT116

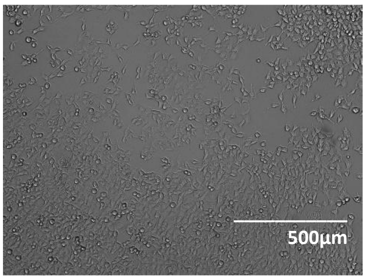

MJ3 6,25µM HCT116

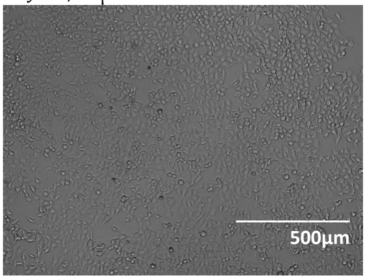

MJ3 12,5µM HCT116

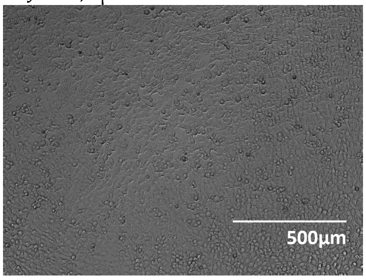

MJ3 25µM HCT116

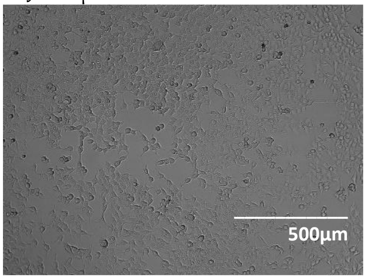

MJ3 50µM HCT116

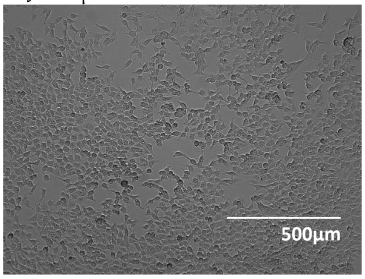

MJ3 100µM HCT116

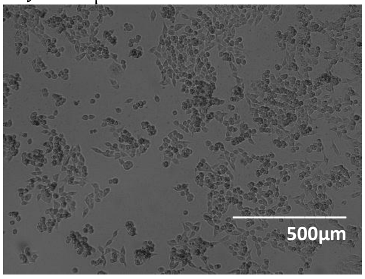

Control HCT116

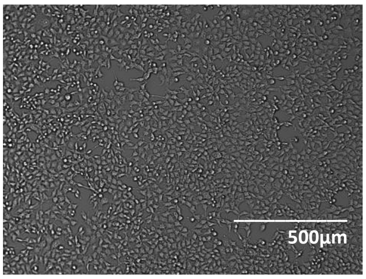

MJ5 6,25µM HCT116

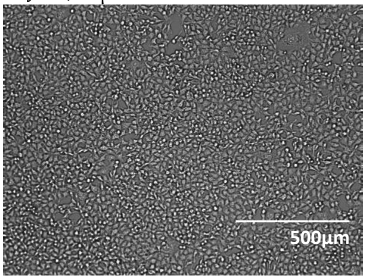

MJ5 12,5µM HCT116

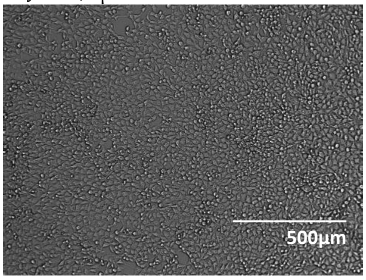

MJ5 25µM HCT116

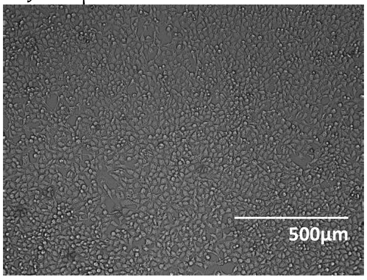

MJ5 50µM HCT116

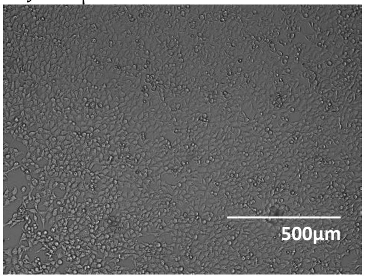

MJ5 100µM HCT116

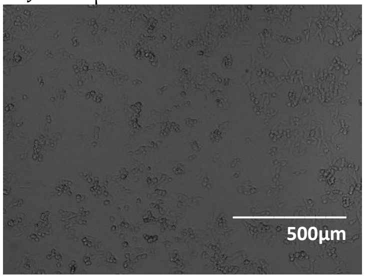

Control HCT116

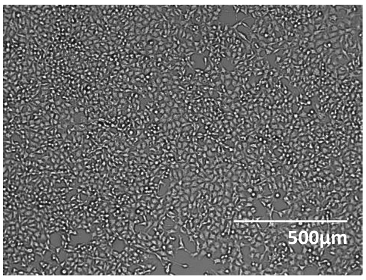

MJ6 6,25µM HCT116

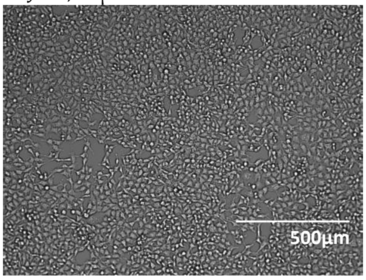

MJ6 12,5µM HCT116

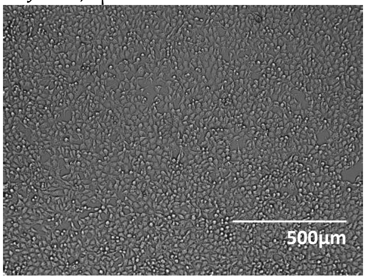

MJ6 25µM HCT116

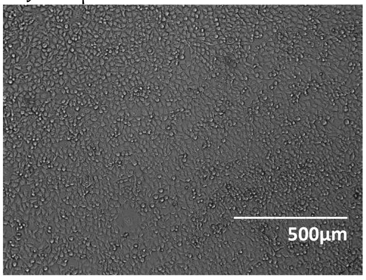

MJ6 50µM HCT116

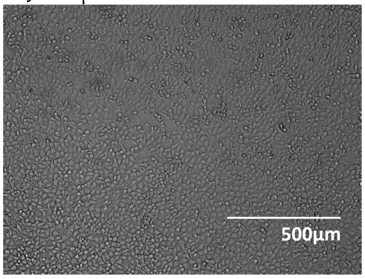

MJ6 100µM HCT116

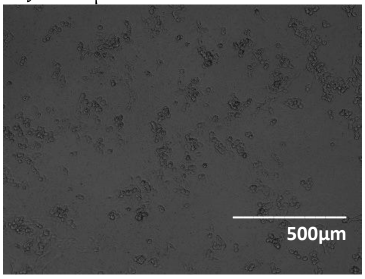

Control HCT116

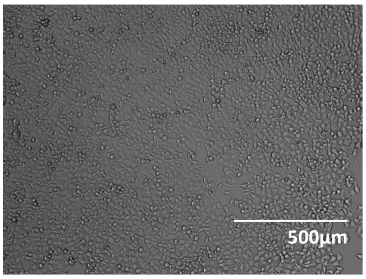

MJ7 6,25µM HCT116

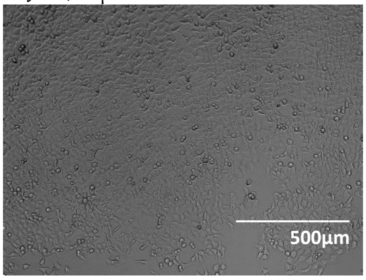

MJ7 12,5µM HCT116

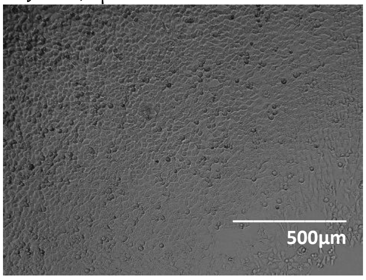

MJ7 25µM HCT116

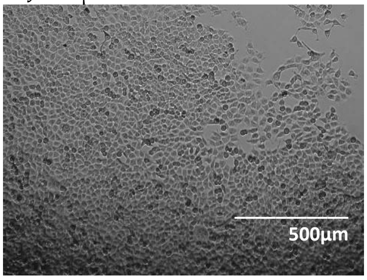

MJ7 50µM HCT116

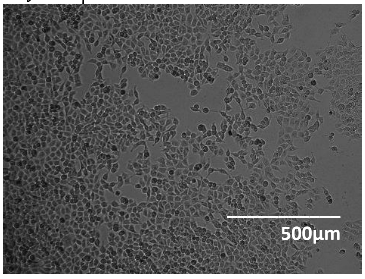

MJ7 100µM HCT116

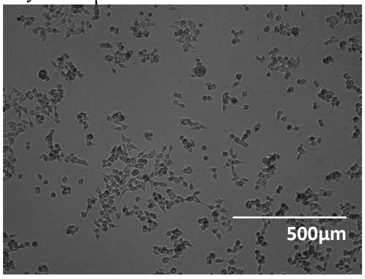

Control HCT116

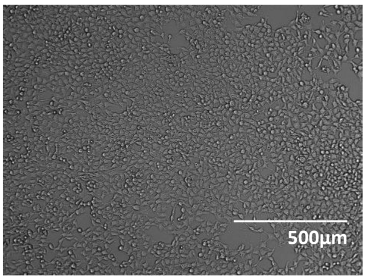

MJ8 6,25µM HCT116

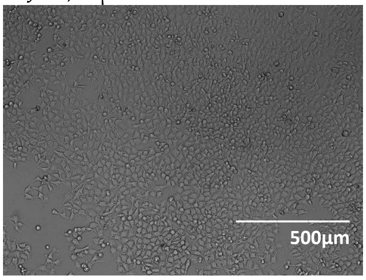

MJ8 12,5µM HCT116

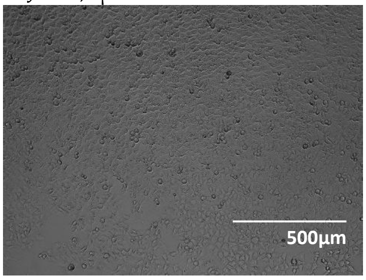

MJ8 25µM HCT116

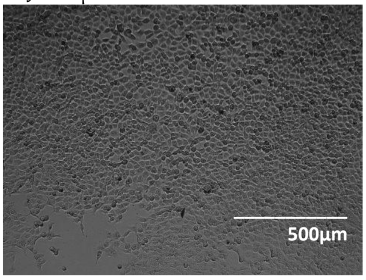

MJ8 50µM HCT116

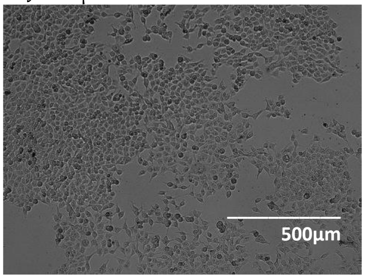

MJ8 100µM HCT116

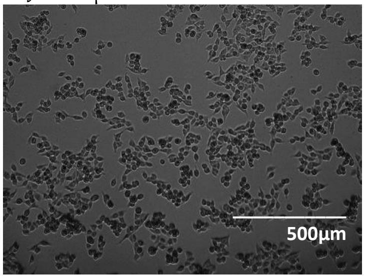

Control HCT116

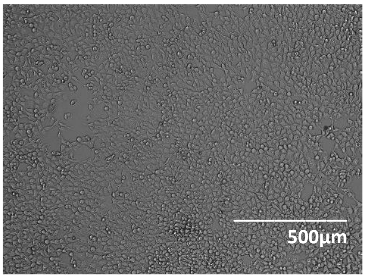

MJ9 6,25µM HCT116

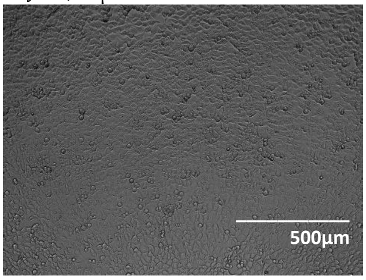

MJ9 12,5µM HCT116

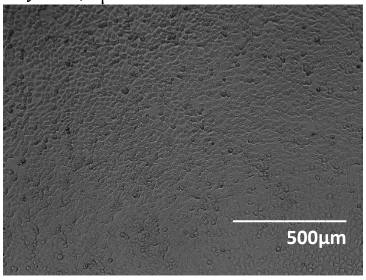

MJ9 25µM HCT116

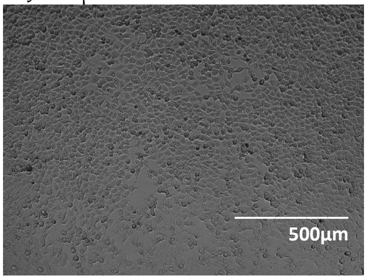

MJ9 50µM HCT116

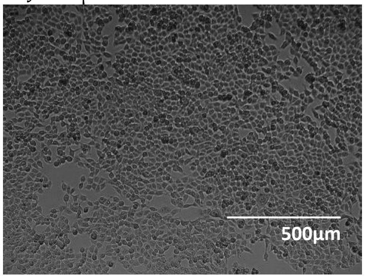

MJ9 100µM HCT116

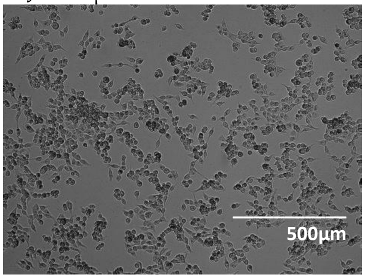

Control HCT116

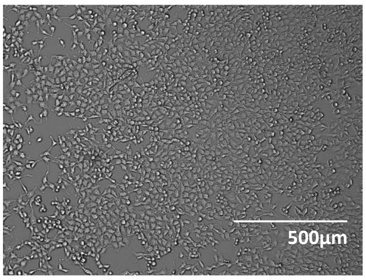

MJ10 6,25µM HCT116

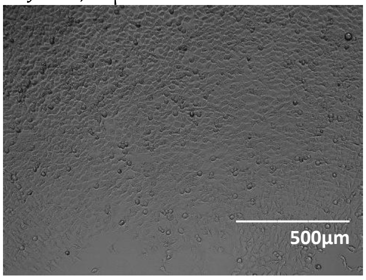

MJ10 12,5µM HCT116

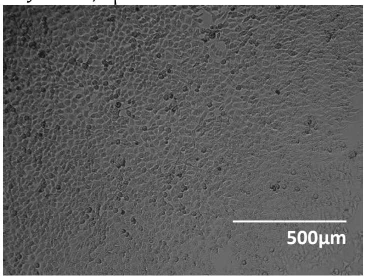

MJ10 25µM HCT116

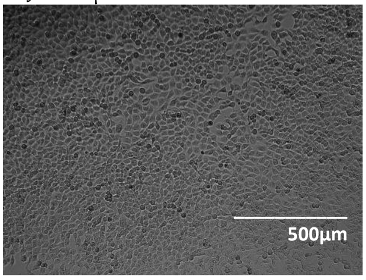

MJ10 50µM HCT116

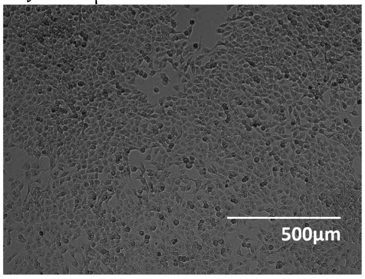

MJ10 100µM HCT116

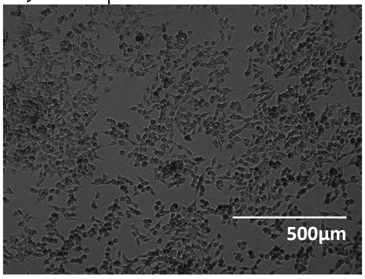

Control HCT116

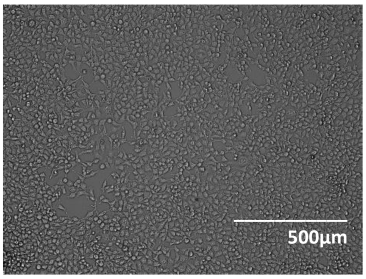

MJ11 6,25µM HCT116

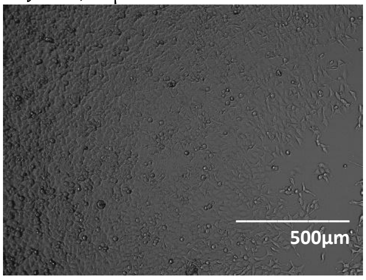

MJ11 12,5µM HCT116

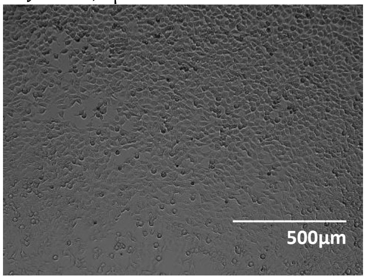

MJ11 25µM HCT116

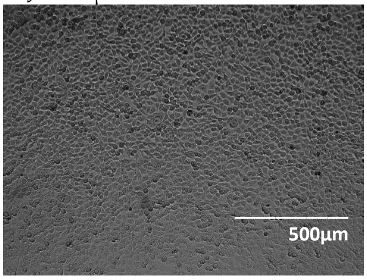

MJ11 50µM HCT116

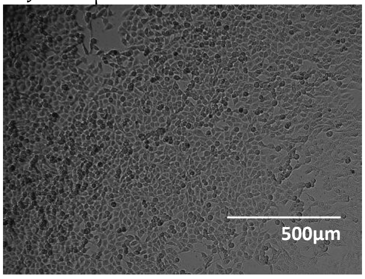

MJ11 100µM HCT116

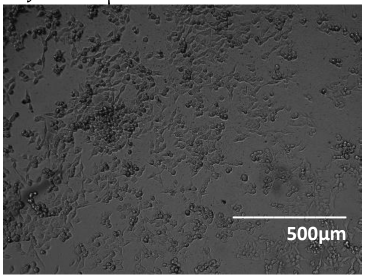

Control HCT116

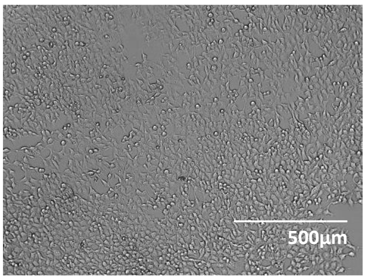

MJ12 6,25µM HCT116

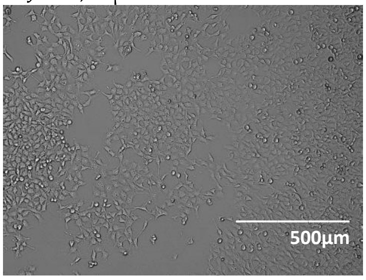

MJ12 12,5µM HCT116

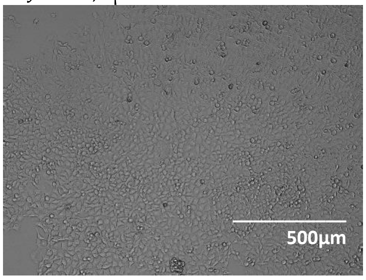

MJ12 25µM HCT116

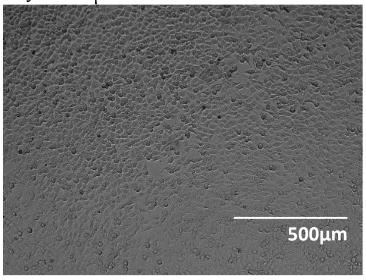

MJ12 50µM HCT116

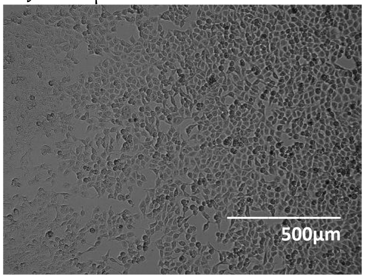

MJ12 100µM HCT116

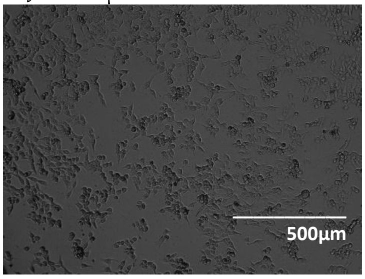

Control HCT116

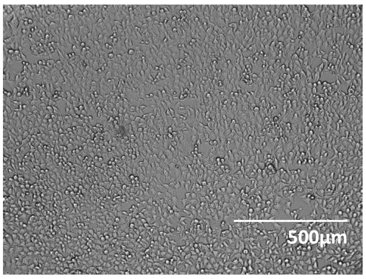

MJ13 6,25µM HCT116

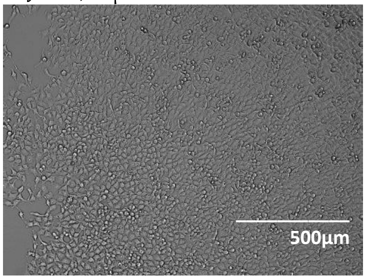

MJ13 12,5µM HCT116

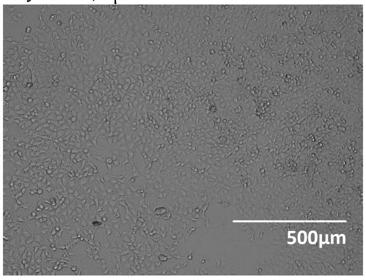

MJ13 25µM HCT116

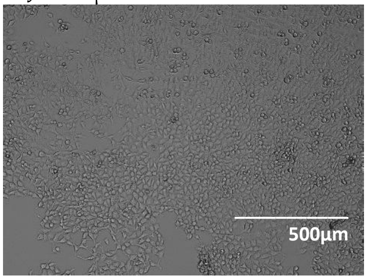

MJ13 50µM HCT116

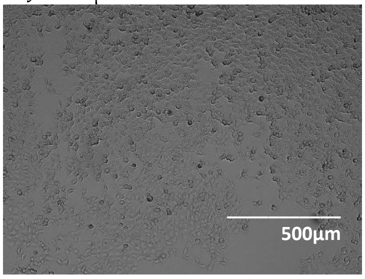

MJ13 100µM HCT116

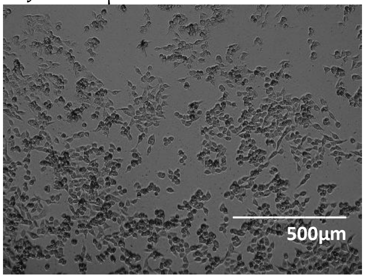

Control HCT116

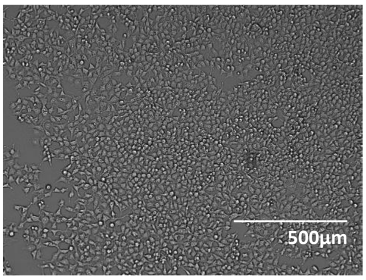

MJ14 6,25µM HCT116

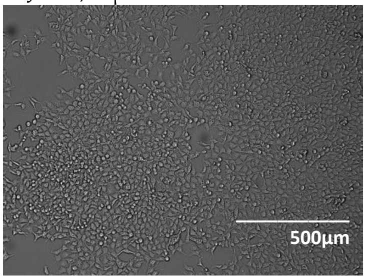

MJ14 12,5µM HCT116

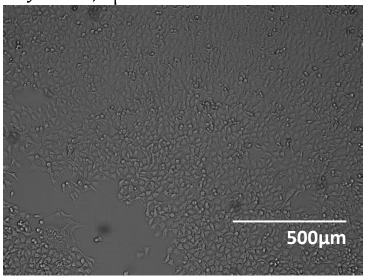

MJ14 25µM HCT116

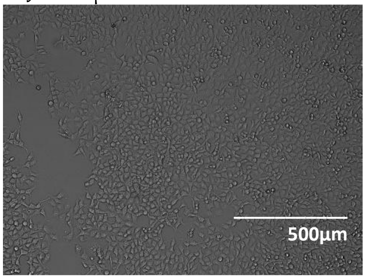

MJ14 50µM HCT116

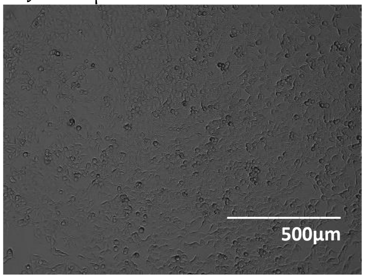

MJ14 100µM HCT116

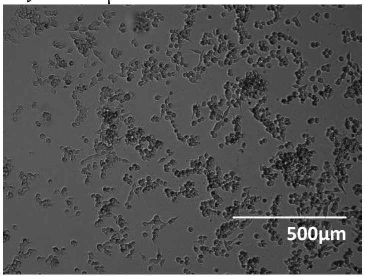

Control HCT116

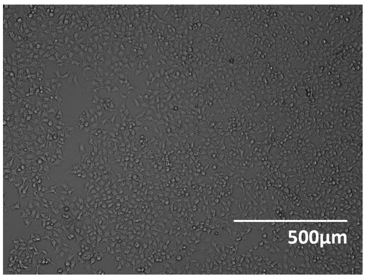

MJ15 6,25µM HCT116

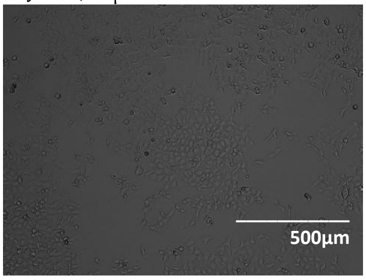

MJ15 12,5µM HCT116

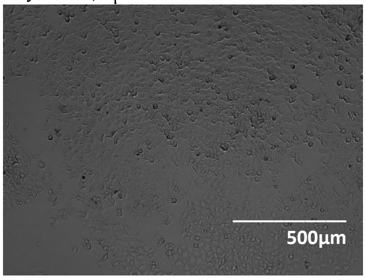

MJ15 25µM HCT116

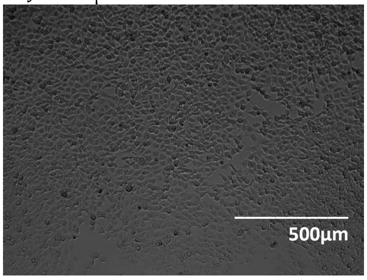

MJ15 50µM HCT116

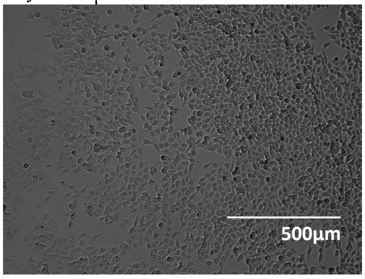

MJ15 100µM HCT116

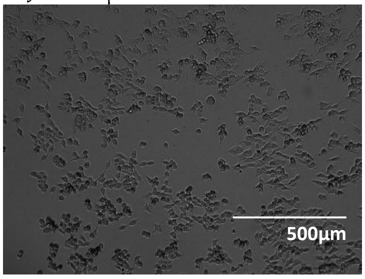

Control HCT116

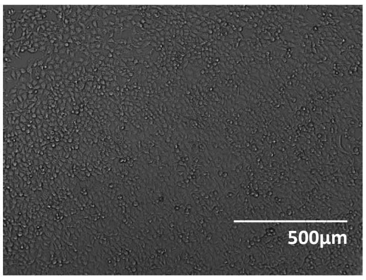

MJ16 6,25µM HCT116

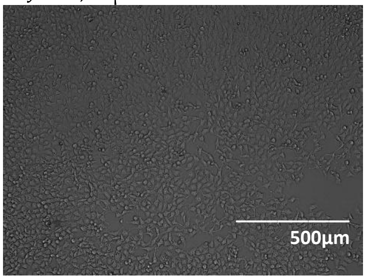

MJ16 12,5µM HCT116

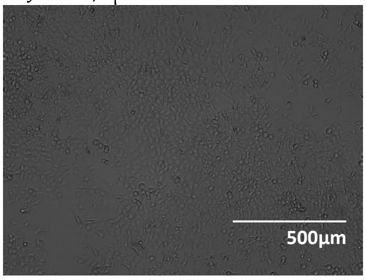

MJ16 25µM HCT116

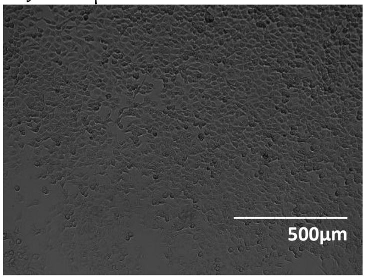

MJ16 50µM HCT116

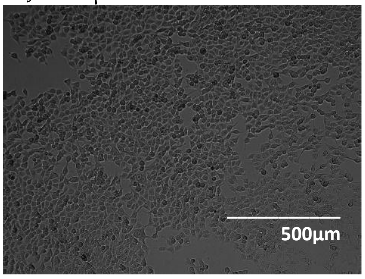

MJ16 100µM HCT116

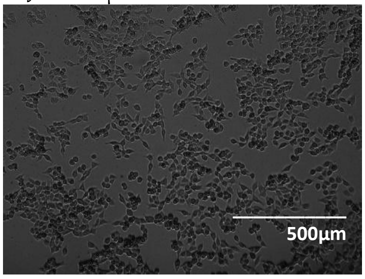

Control HCT116

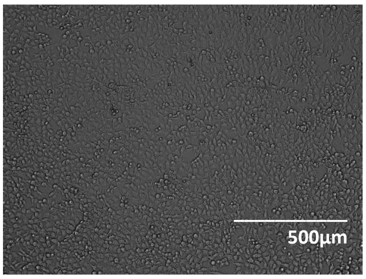

MJ17 6,25µM HCT116

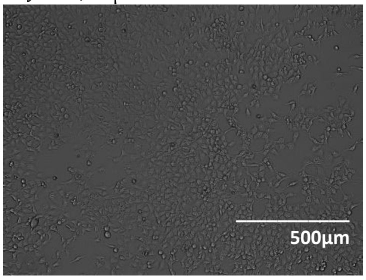

MJ17 12,5µM HCT116

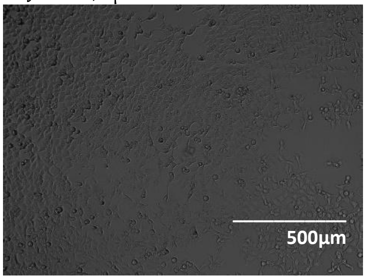

MJ17 25µM HCT116

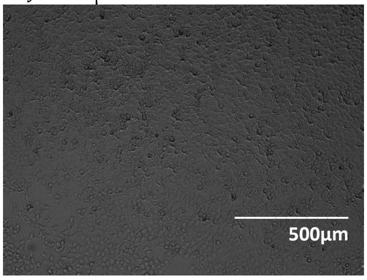

MJ17 50µM HCT116

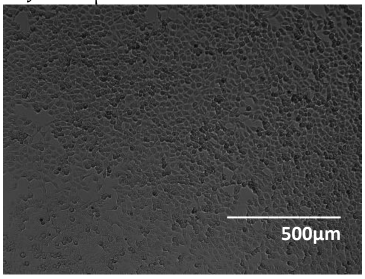

MJ17 100µM HCT116

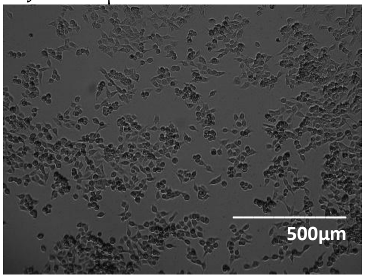

Control HCT116

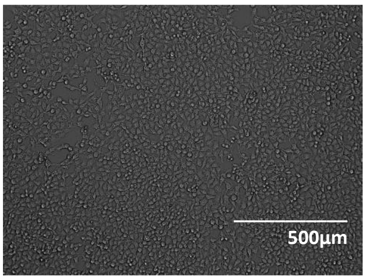

MJ18 6,25μM HCT116

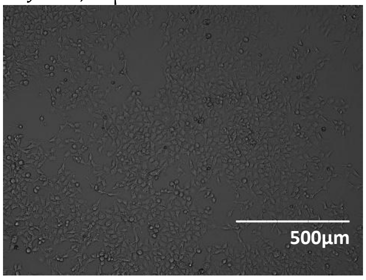

MJ18 12,5μM HCT116

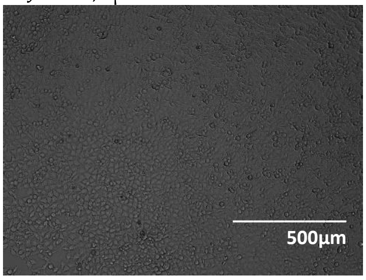

MJ18 25μM HCT116

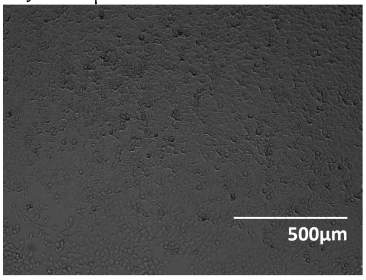

MJ18 50μM HCT116

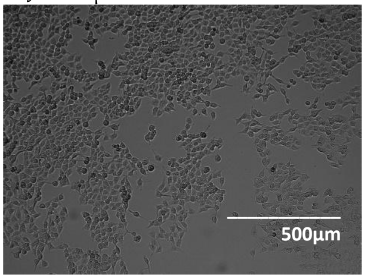

MJ18 100μM HCT116

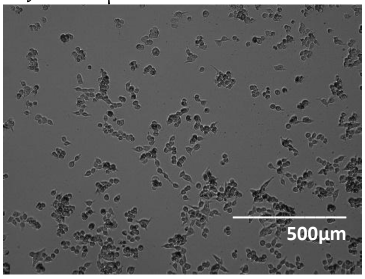

Control HCT116

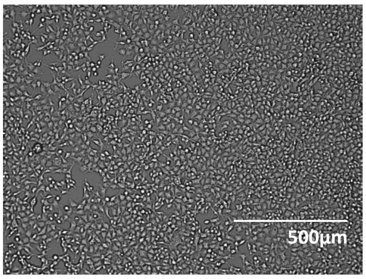

MJ19 6,25μM HCT116

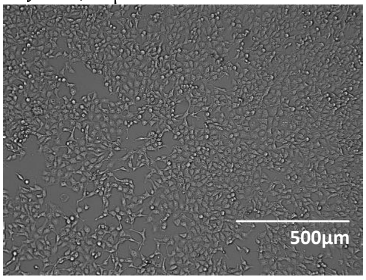

MJ1912,5μM HCT116

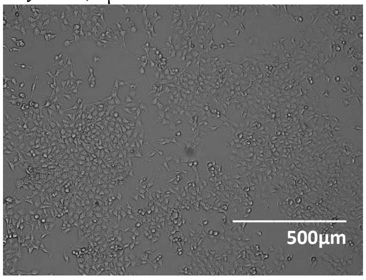

MJ19 25μM HCT116

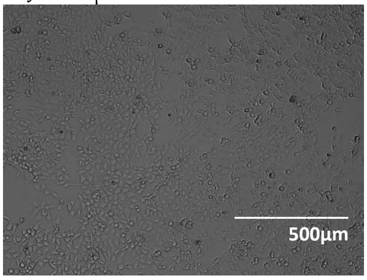

MJ19 50μM HCT116

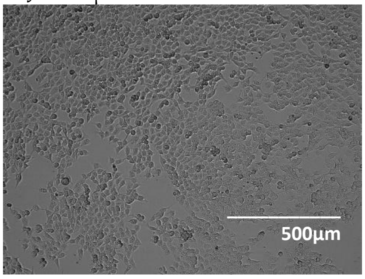

MJ19 100μM HCT116

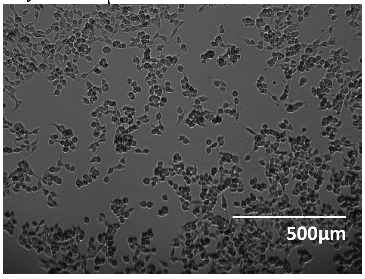

Control HCT116

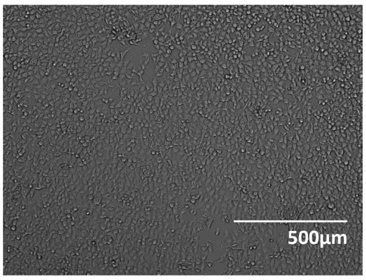

MJ20 6,25µM HCT116

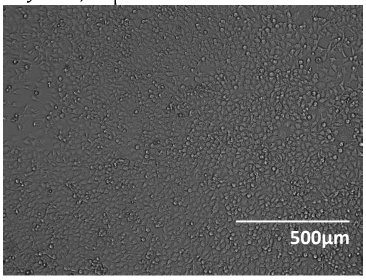

MJ20 12,5µM HCT116

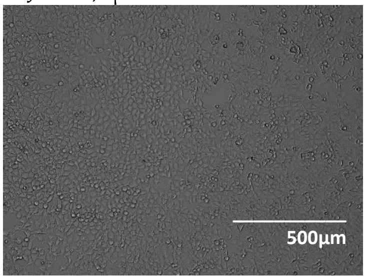

MJ20 25µM HCT116

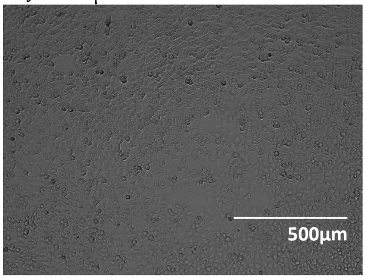

MJ20 50µM HCT116

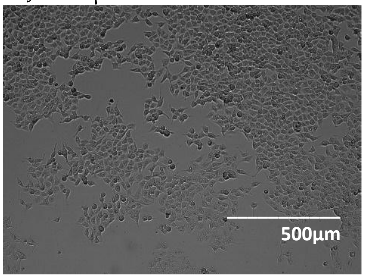

MJ20 100µM HCT116

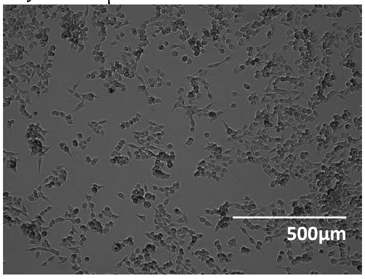

4. HCT116 MJ1-MJ20 (100μM)

Control HCT116

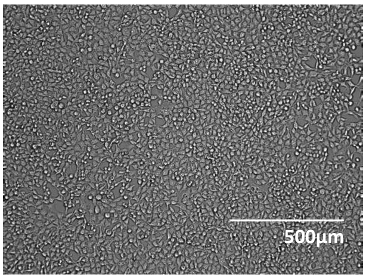

MJ1 100μM HCT116

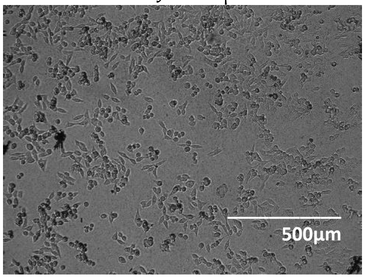

MJ2 100μM HCT116

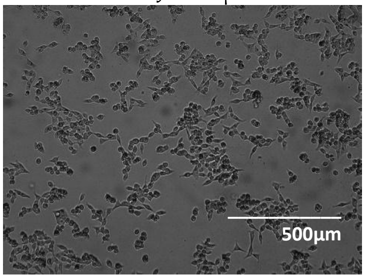

MJ3 100μM HCT116

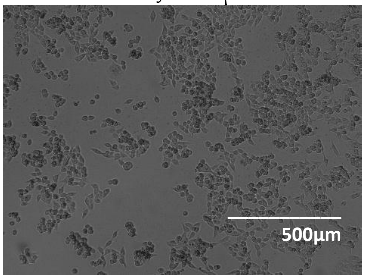

MJ4 100μM HCT116

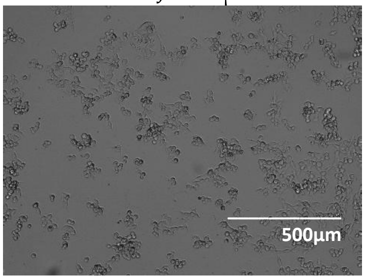

MJ5 100μM HCT116

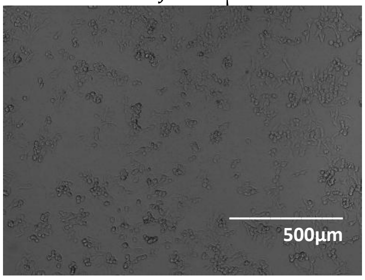

MJ6 100μM HCT116

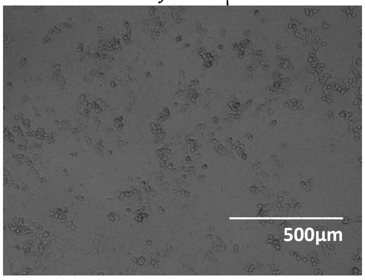

MJ7 100μM HCT116

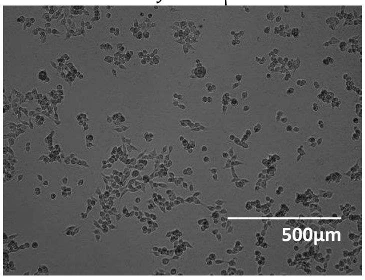

MJ8 100μM HCT116

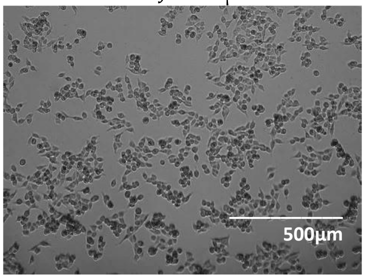

MJ9 100μM HCT116

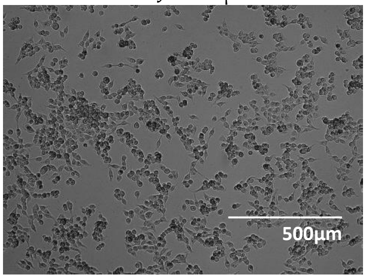

MJ10 100μM HCT116

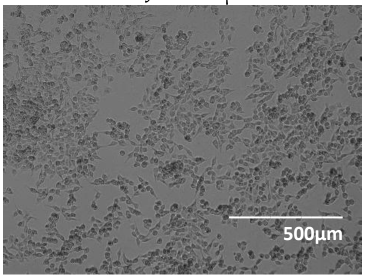

MJ11 100μM HCT116

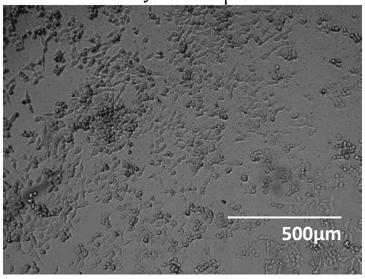

MJ12 100μM HCT116

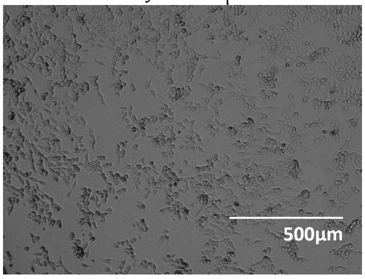

MJ13 100μM HCT116

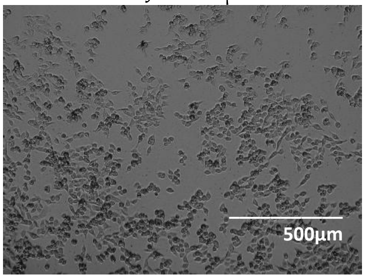

MJ14 100μM HCT116

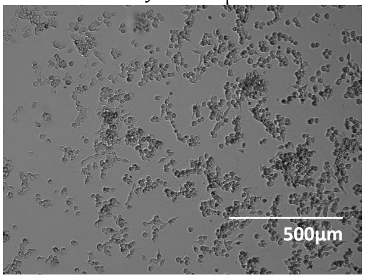

MJ15 100μM HCT116

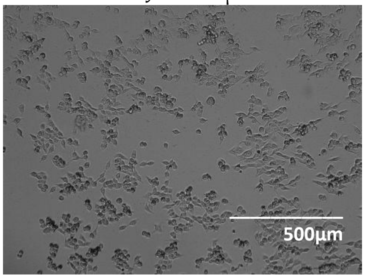

MJ16 100μM HCT116

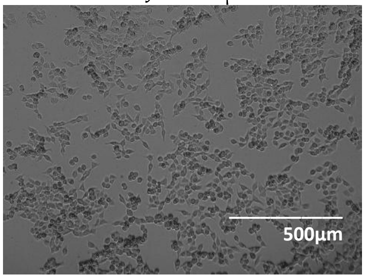

MJ17 100μM HCT116

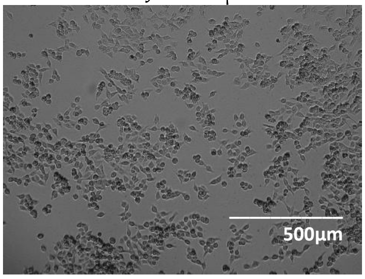

MJ18 100μM HCT116

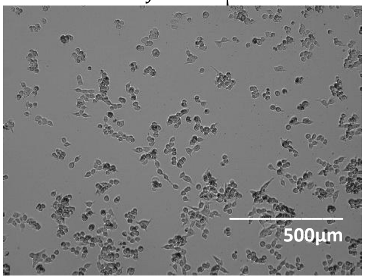

MJ19 100μM HCT116

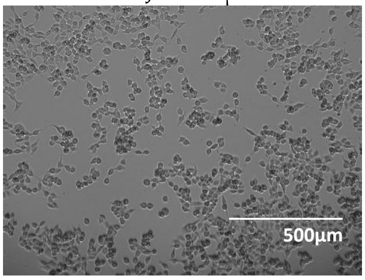

MJ20 100μM HCT116

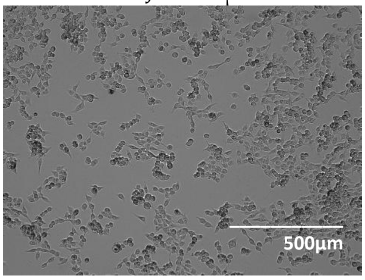

5. MCF7 MJ1-MJ20 (100μM)

Control MCF7

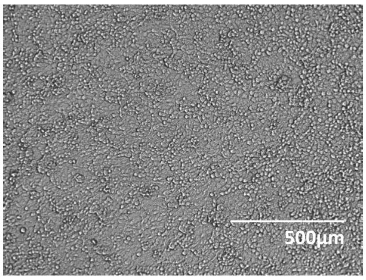

MJ1 100μM MCF7

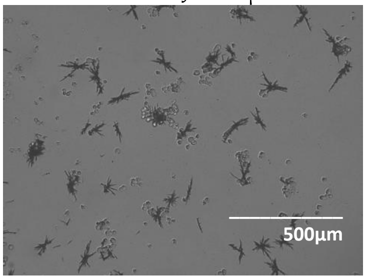

MJ2 100μM MCF7

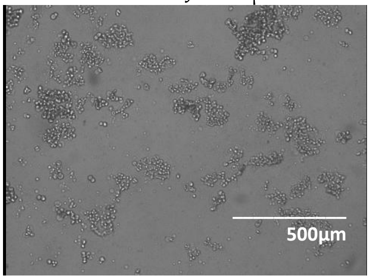

MJ3 100μM MCF7

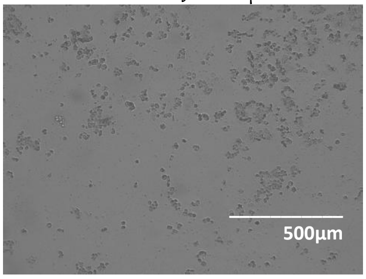

MJ4 100μM MCF7

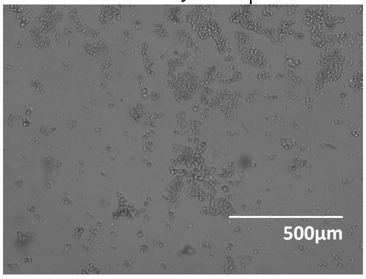

MJ5 100μM MCF7

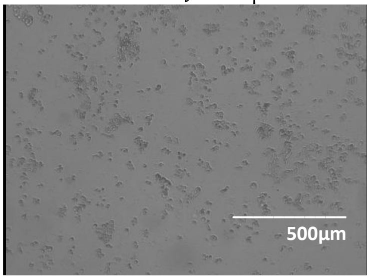

MJ6 100μM MCF7

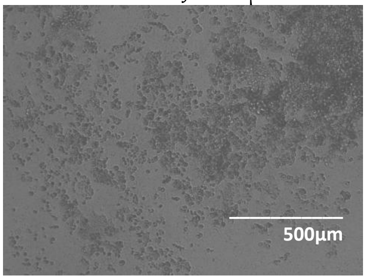

MJ7 100μM MCF7

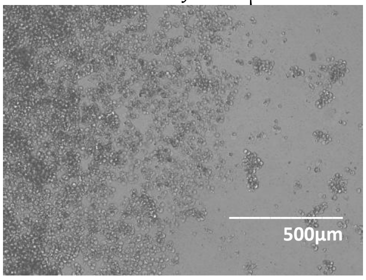

MJ8 100μM MCF7

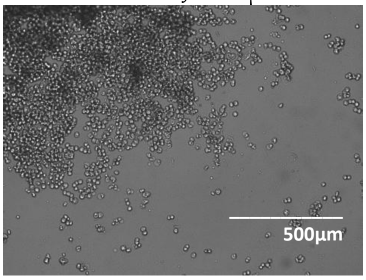

MJ9 100μM MCF7

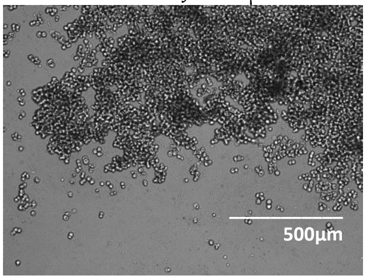

MJ10 100μM MCF7

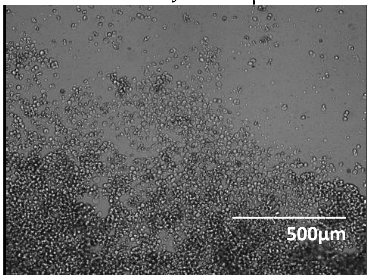

MJ11 100μM MCF7

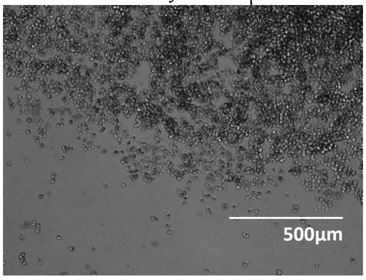

MJ12 100μM MCF7

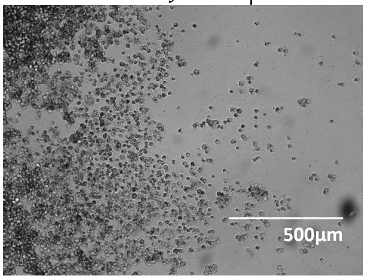

MJ13 100μM MCF7

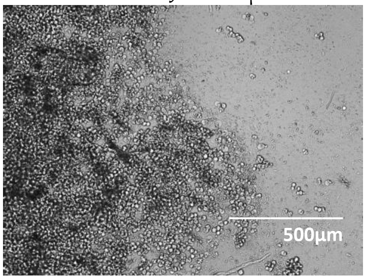

MJ14 100μM MCF7

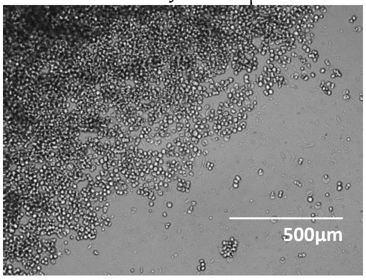

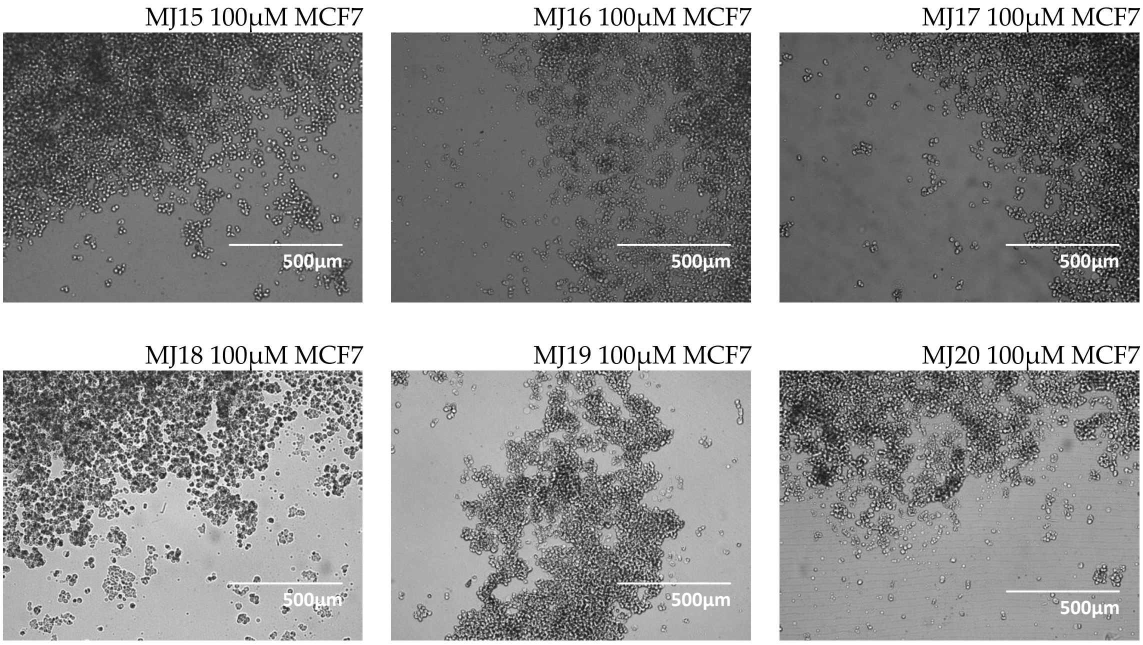
MJ15 100μM MCF7
500μm
MJ16 100μM MCF7
500μm
MJ17 100μM MCF7
500μm
MJ18 100μM MCF7
500μm
MJ19 100μM MCF7
500μm
MJ20 100μM MCF7
500μm

6. NHDF MJ1-MJ20 (100μM)

Control NHDF

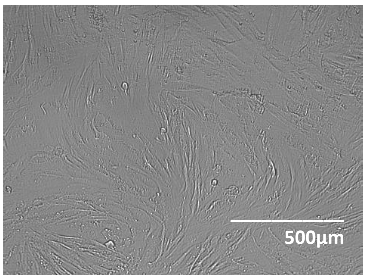

MJ1 100μM NHDF

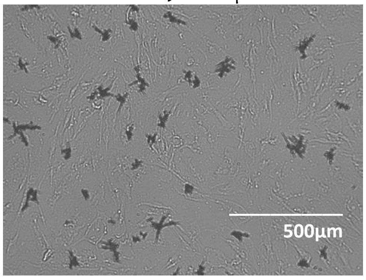

MJ2 100μM NHDF

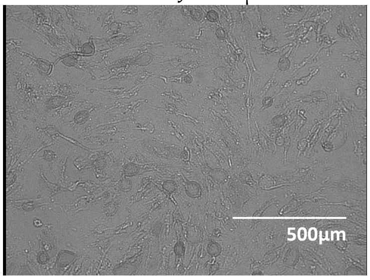

MJ3 100μM NHDF

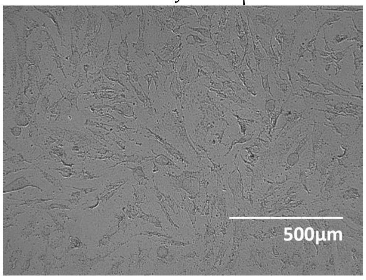

MJ4 100μM NHDF

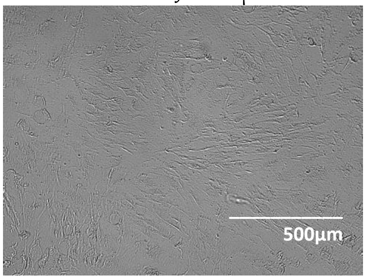

MJ5 100μM NHDF

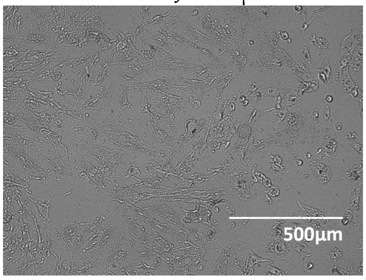

MJ6 100μM NHDF

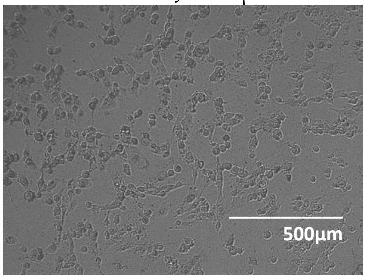

MJ7 100μM NHDF

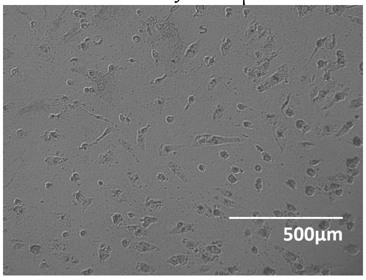

MJ8 100μM NHDF

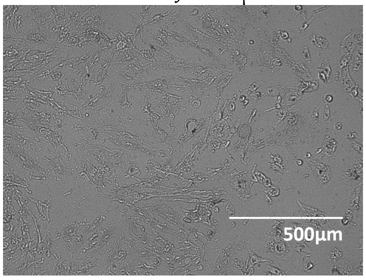

MJ9 100μM NHDF

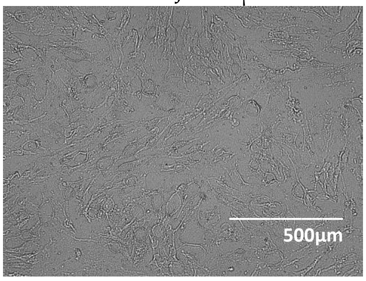

MJ10 100μM NHDF

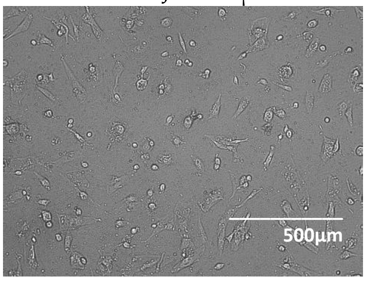

MJ11 100μM NHDF

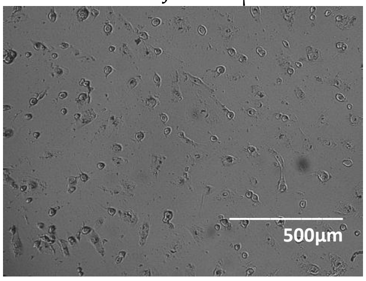

MJ12 100μM NHDF

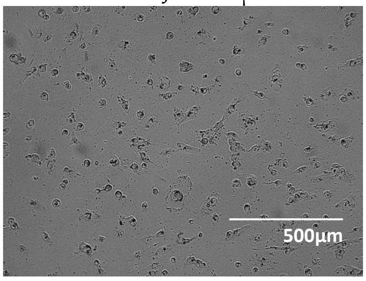

MJ13 100μM NHDF

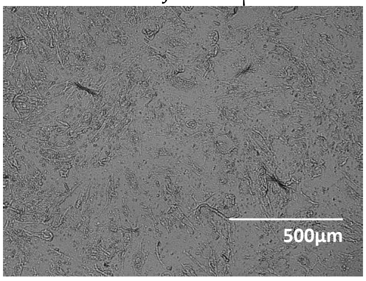

MJ14 100μM NHDF

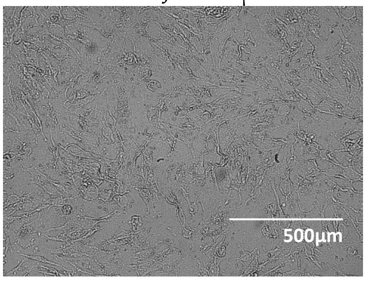

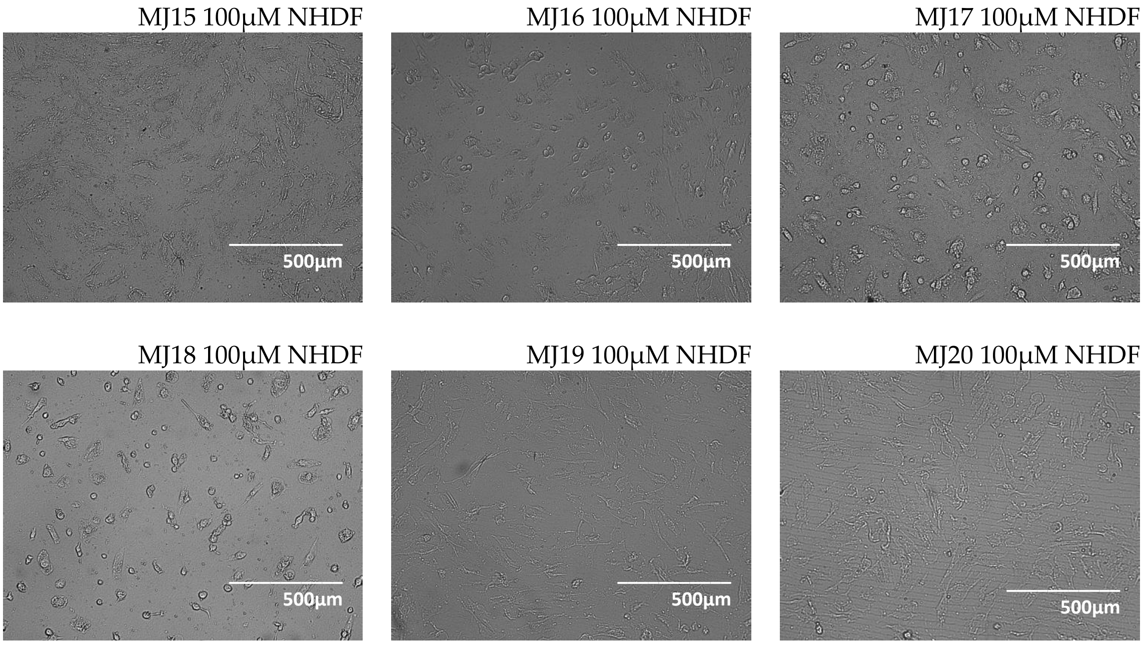
MJ15 100μM NHDF
500μm
MJ16 100μM NHDF
500μm
MJ17 100μM NHDF
500μm
MJ18 100μM NHDF
500μm
MJ19 100μM NHDF
500μm
MJ20 100μM NHDF
500μm

7. SH-SY5Y MJ1-MJ20 (100µM)

Control SH-SY5Y

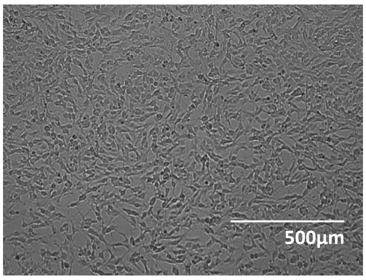

MJ1 100µM SH-SY5Y

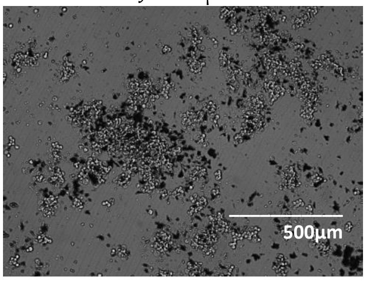

MJ2 100µM SH-SY5Y

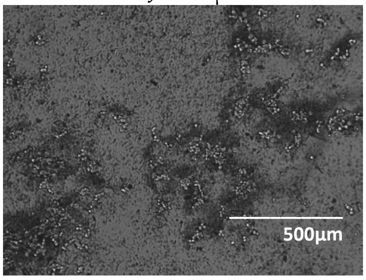

MJ3 100µM SH-SY5Y

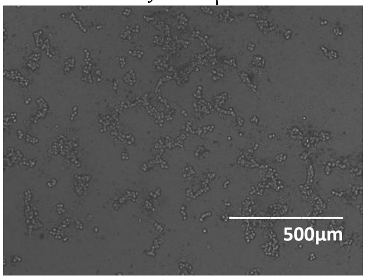

MJ4 100µM SH-SY5Y

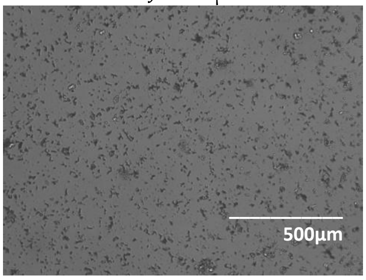

MJ5 100µM SH-SY5Y

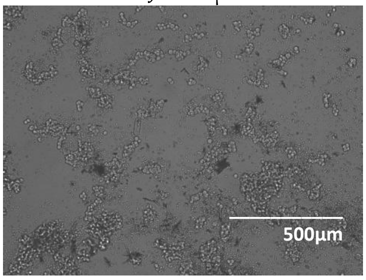

MJ6 100µM SH-SY5Y

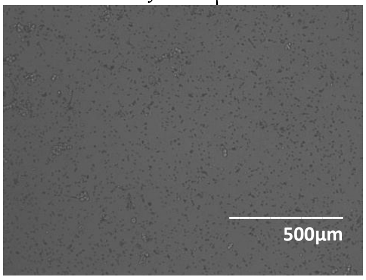

MJ7 100µM SH-SY5Y

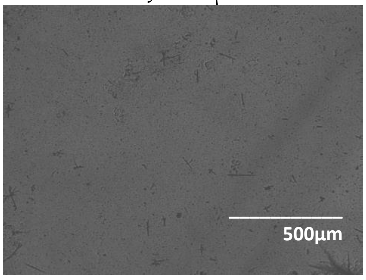

MJ8 100µM SH-SY5Y

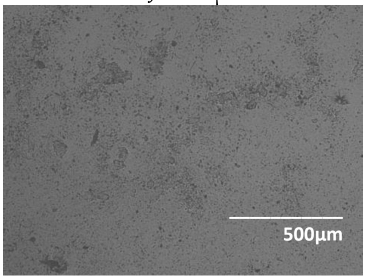

MJ9 100µM SH-SY5Y

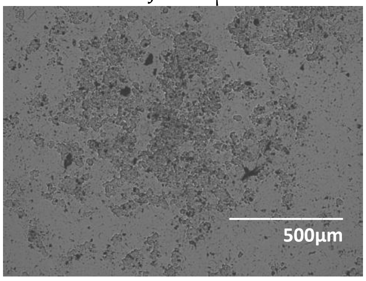

MJ10 100µM SH-SY5Y

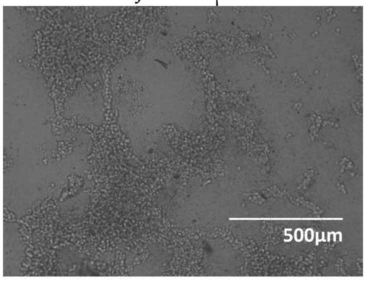

MJ11 100µM SH-SY5Y

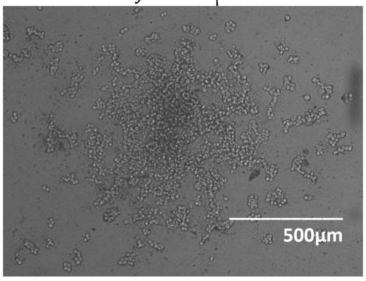

MJ12 100µM SH-SY5Y

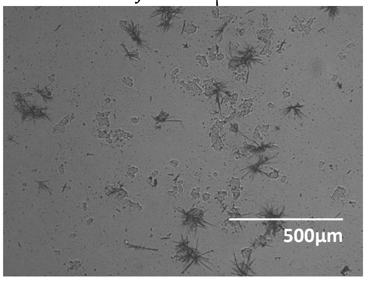

MJ13 100µM SH-SY5Y

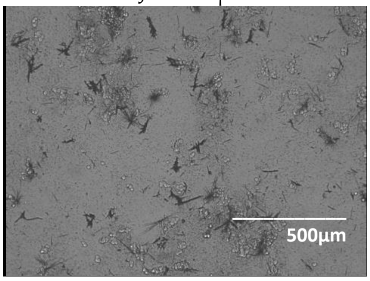

MJ14 100µM SH-SY5Y

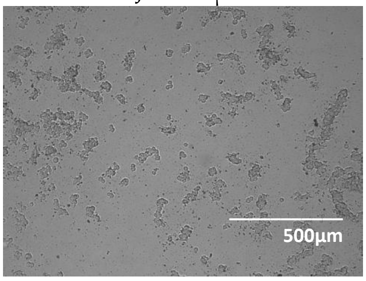

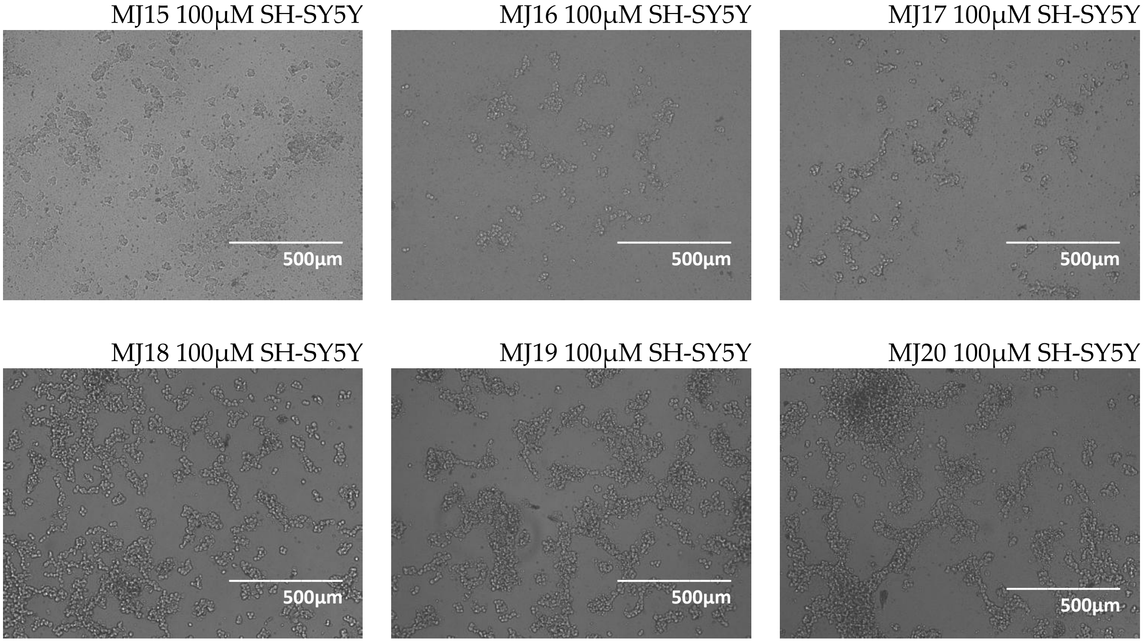
MJ15 100μM SH-SY5Y
500μm
MJ16 100μM SH-SY5Y
500μm
MJ17 100μM SH-SY5Y
500μm
MJ18 100μM SH-SY5Y
500μm
MJ19 100μM SH-SY5Y
500μm
MJ20 100μM SH-SY5Y
500μm

8. A549 MJ1-MJ20 (100μM)

Control A549

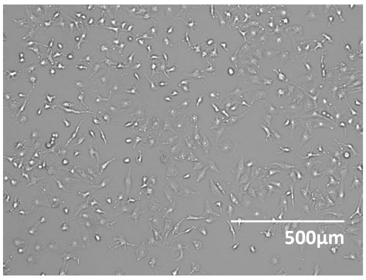

MJ1 100μM A549

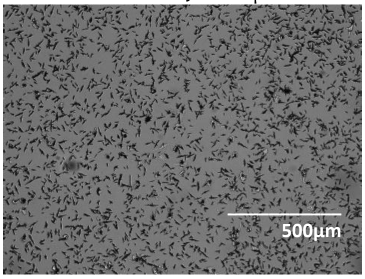

MJ2 100μM A549

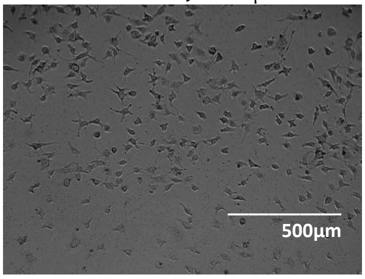

MJ3 100μM A549

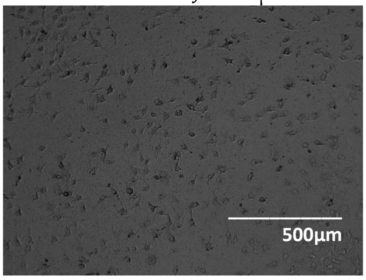

MJ4 100μM A549

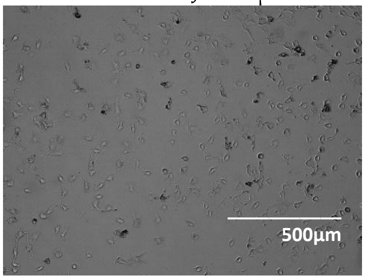

MJ5 100μM A549

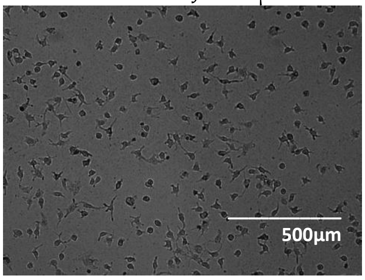

MJ6 100μM A549

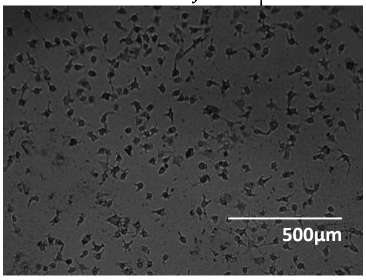

MJ7 100μM A549

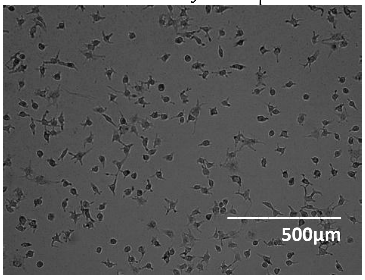

MJ8 100μM A549

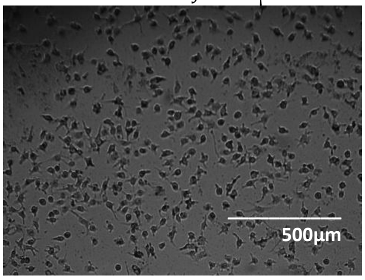

MJ9 100μM A549

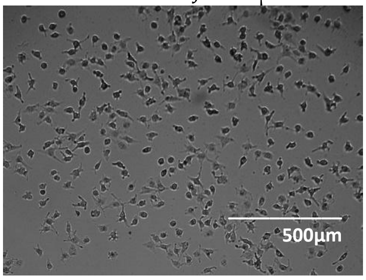

MJ10 100μM A549

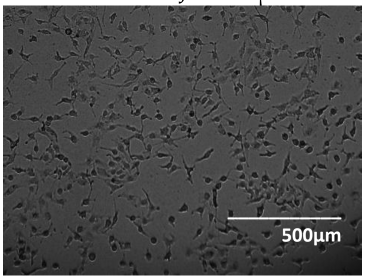

MJ11 100μM A549

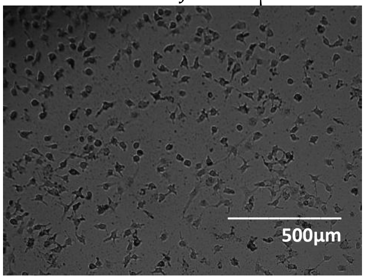

MJ12 100μM A549

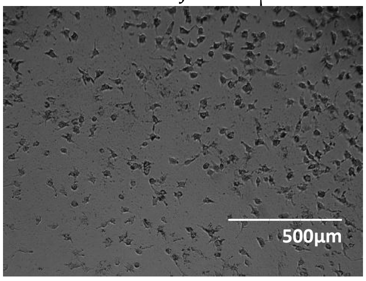

MJ13 100μM A549

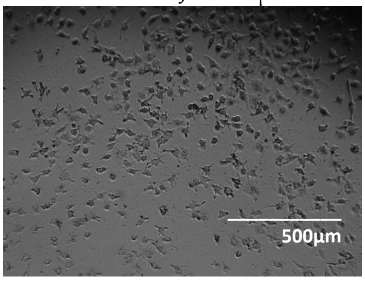

MJ14 100μM A549

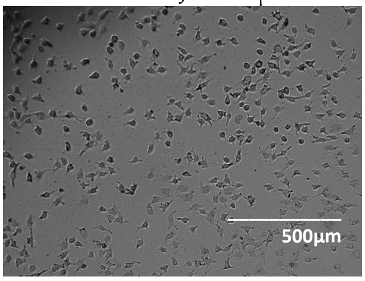

MJ15 100µM A549

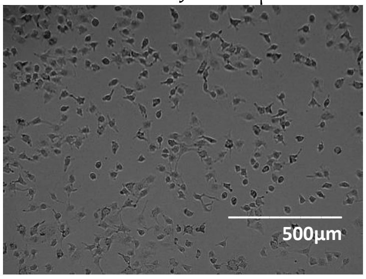

MJ16 100µM A549

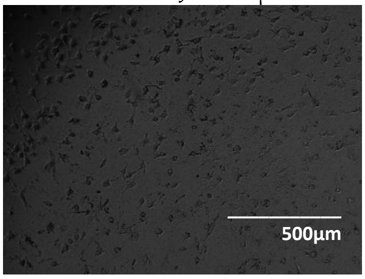

MJ17 100µM A549

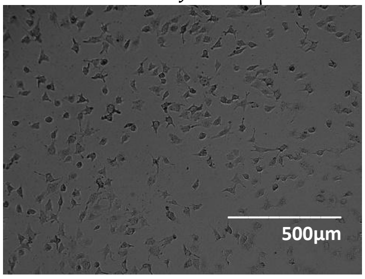

MJ18 100µM A549

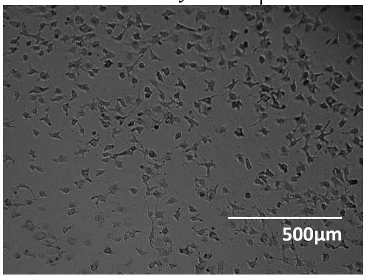

MJ19 100µM A549

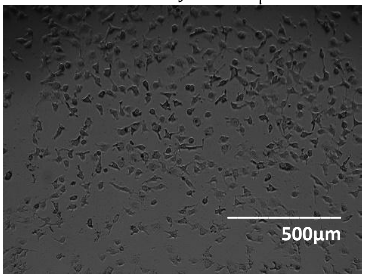

MJ20 100µM A549

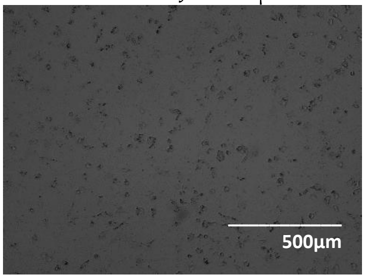

9. **Images of BEAS-2B cell line nuclei after 24h of incubation with MJ2, MJ8, MJ15, MJ19 compounds at concentration of 100µM after DAPI staining.**

BEAS-2B Control

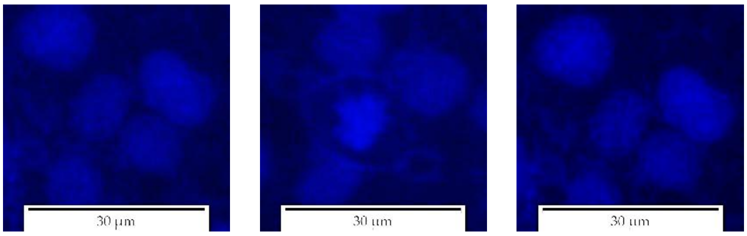

BEAS-2B MJ2

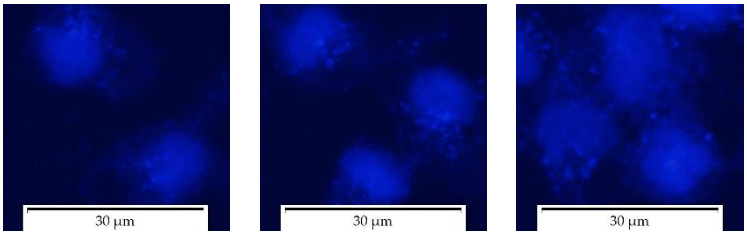

BEAS-2B MJ8

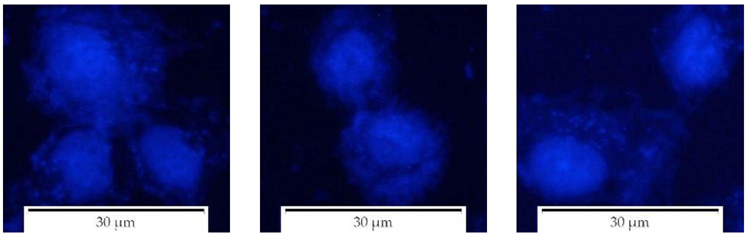

BEAS-2B MJ15

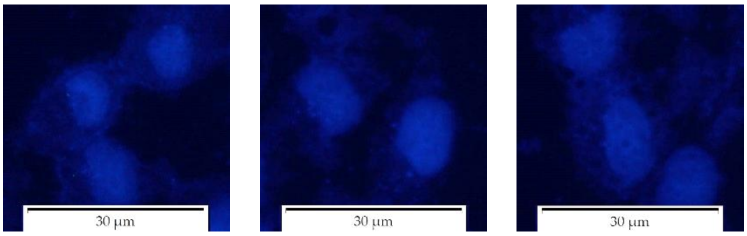

BEAS-2B MJ19

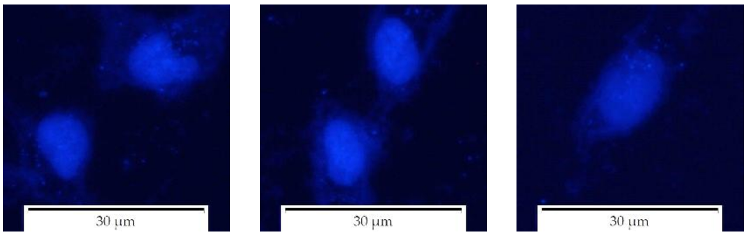

10. Images of HCT116 cell line nuclei after 24h of incubation with MJ2, MJ8, MJ15, MJ19 compounds at concentration of 100µM after DAPI staining.

HCT116 Control

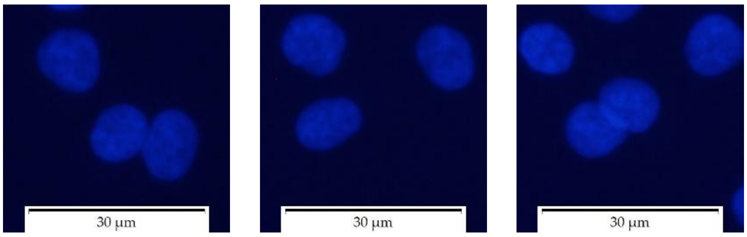

HCT116 MJ2

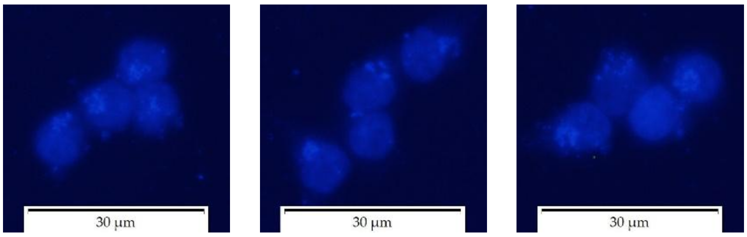

HCT116 MJ8

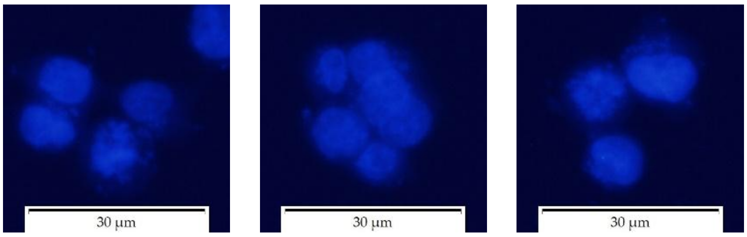

HCT116 MJ15

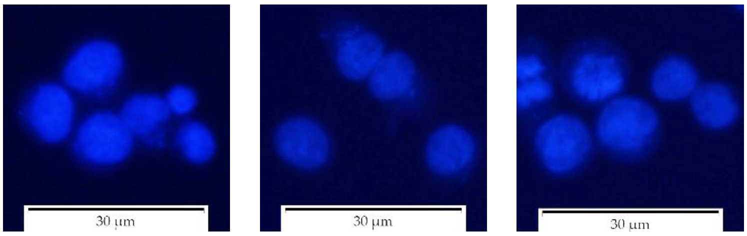

HCT116 MJ19

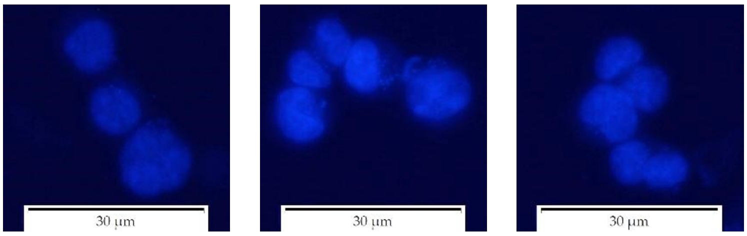

Supplement: Supplementary file 1 [file ijms-26-06920-s001.zip › Supplementary Materials 2.pdf]
